# Comparison of Physicochemical Properties, Antioxidants, and Aroma Profiles of Water- and Sodium-Hydroxide-Treated Natural Cocoa Powder

**DOI:** 10.1021/acsomega.4c04173

**Published:** 2024-08-05

**Authors:** Ertan Sahin, Fatma Duygu Ceylan, Aslı Barla Demirkoz, Aslı Can Karaca, Esra Capanoglu

**Affiliations:** † Department of Food Engineering, Faculty of Chemical and Metallurgical Engineering, Istanbul Technical University, Maslak, 344469 Istanbul, Türkiye; ‡ Department of Research and Development Center, Aromsa Flavours and Food Additives Industry and Trade Inc. Co., Gebze, 41480 Kocaeli, Türkiye; § Department of Analytical Chemistry, Faculty of Pharmacy, Istanbul Okan University, Tuzla, 34959 Istanbul, Türkiye

## Abstract

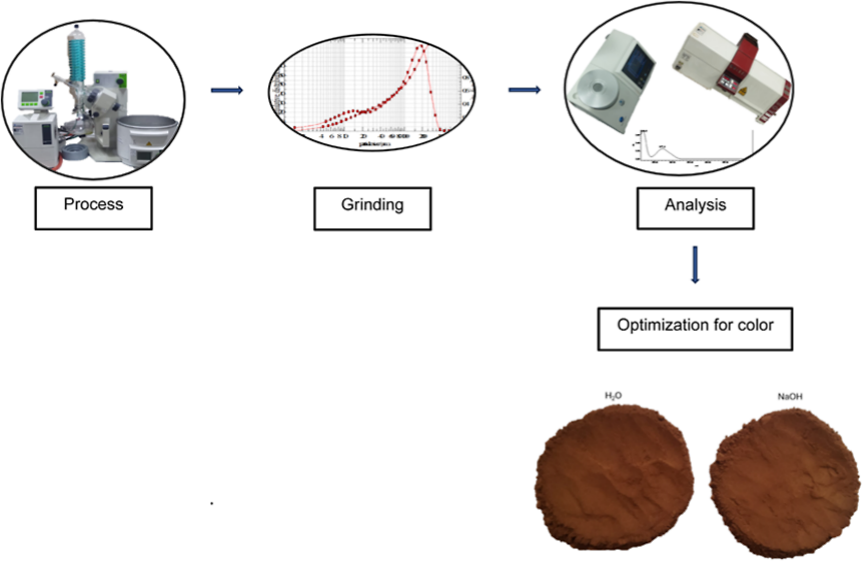

Cocoa powder alkalization is an essential process in chocolate manufacturing, and traditionally, this process involves the use of alkaline agents, such as sodium hydroxide (NaOH), potassium hydroxide (KOH), and potassium carbonate (K_2_CO_3_). However, these methods involve harsh chemicals and energy-intensive procedures, raising significant environmental concerns. Water (H_2_O) has emerged as a promising alternative due to its safety, minimally harmful byproducts, and accessibility. Green chemistry principles have gained importance across industries, especially in food production, where sustainable practices are highly valued. This study aimed to develop a greener process by investigating the alkalization potential of H_2_O and comparing the results with those of NaOH. The particle size distribution, pH, color, antioxidant capacity, phenolic composition, and aroma profile of cocoa powders treated with H_2_O and NaOH were evaluated. The alkalization temperature significantly affected the color of the cocoa powders, and the alkali solution ratio influenced the L* values of H_2_O-treated powders. In industrial and commercial specifications, an Δ*E* value below 3 is considered standard for color measurements. Both H_2_O-treated and NaOH-treated natural cocoa powders had Δ*E* values exceeding 3 compared to the untreated powder, indicating that H_2_O treatment darkens the color in a similar way to that of traditional methods. While NaOH produced a darker color, process optimization allowed both H_2_O and NaOH treatments to achieve similar color attributes (Δ*E* < 3). Significant differences were observed in the antioxidant capacity and total phenolic content (TPC) between the H_2_O-treated and NaOH-treated cocoa powders. H_2_O treatment positively impacted the antioxidative properties of the cocoa powder. The antioxidant capacity, measured by the DPPH and CUPRAC methods, was significantly higher in H_2_O-treated samples (295.5–317.7 TEAC mg/100 g and 835–1542 TEAC mg/100 g, respectively) compared to NaOH-treated samples (256.6–306.2 TEAC mg/100 g and 171–849 TEAC mg/100 g, respectively). Additionally, the TPC of H_2_O-treated cocoa powder [281.3–321.6 gallic acid equivalent (GAE) mg/100 g] was significantly higher than that of NaOH-treated powder (100.0–298.6 GAE mg/100 g). The significant differences in the phenolic profiles suggested that the alkalization process affects individual phenolic compounds differently. Moreover, H_2_O-treated cocoa powders had significantly higher trimethylpyrazine/tetramethylpyrazine (TrMP/TMP) ratios than those of NaOH-treated samples, indicating notable differences in aroma profiles. This study suggests that H_2_O can replace NaOH in the alkalization process of the cocoa industry, particularly for lightly treated alkalized cocoa powders that maintain high antioxidant activity and TrMP/TMP ratios. This offers a more environmentally friendly, easily manageable, and sustainable process for cocoa powder alkalization.

## Introduction

Green chemistry has become a driving force for sustainable development and has gained significant prominence in recent years. The increasing global population and elevated living standards have created an awareness of the environmental impacts of hazardous materials and resource degradation.^1^ The industry faces the formidable challenge of transitioning to greener manufacturing processes, optimizing raw material utilization, and minimizing waste generation.^2^ Recent developments in green chemistry research include proposing green chemistry metrics and innovative business models.^3^ The Sustainable Development Goals, introduced by the United Nations in 2015, aim to enhance global welfare by addressing social, environmental, and economic sustainability.^4^ Sustainable chemistry, encompassing water, energy, food, climate, and population, significantly contributes to human health and the well-being of living species.^5^ During the alkalization process, the excess alkali solution added to natural cocoa is removed through the application of vacuum and heat. This removal leads to the release of the solution, resulting in undesired odors at the factory location. In the production of black cocoa powder, ammonium bicarbonate, used as an alkaline agent, releases ammonia, which can be toxic. This has led to complaints from residents living around the relevant factory in The Netherlands, resulting in media coverage and prompting the company to change the process. Consequently, there is a need to explore alternative methods to replace the alkaline agents. For example, a global alkalized cocoa powder producer’s patent introduces a novel alkalization process for cocoa beans, aiming to avoid environmentally harmful iron salts by utilizing a combination of ammonium carbonate, sodium hydroxide, and potassium hydroxide, aligning with green chemistry principles.

Sodium hydroxide (NaOH), commonly used for various purposes in the manufacturing sector, is particularly favored for alkalization processes. However, its production involves high electricity consumption, which contributes to the increased global warming potential. The production of 1 kg of sodium hydroxide results in 0.6329 kg of CO_2_ equivalents, which contributes to its global warming potential.^6^ Additionally, NaOH has toxic effects on aquatic ecosystems, human health, and acidification. To mitigate these negative environmental impacts, substituting water for NaOH in alkalization processes can be an effective solution. Given the urgency of addressing climate change effects, green processes can prevent pollution and minimize adverse environmental impacts.

The alkalization process applied to cocoa cake can be briefly described as introducing an alkali solution to natural cocoa powder within a sealed mixing vessel, followed by a subsequent application of vacuum to eliminate excess moisture through heat treatment.^7^ The resultant products of the alkalization process are characterized as cocoa solids, exhibiting a darker color and high pH levels compared to natural cocoa powder. The alkalization process is affected by factors such as the potency of the alkali solution, the type of alkali, the duration of the reaction phase, and the temperature of the process.^8^ Cocoa has a complex composition comprising various compounds, including carbohydrates, proteins, fats, phenolics, methylxanthines, minerals, vitamins, and amino acids.^9^ Simultaneously, heat treatment during the alkalization process induces the generation of volatile compounds, including aldehydes, alkenes, esters, pyrazines, ketones, and phenols.^10^ The alkalization process encourages multiple chemical transformations capable of altering not only the cocoa composition but also its nutritional, sensory, and microbiological attributes. Under such conditions, phenolic oxidation tends to progress into quinones, concluding with the formation of polymerized or high-molecular-weight brown–red–dark compounds.^7^ The occurrence of these compounds is not attributable to phenolic oxidation; Maillard reactions also play a significant role, leading to the formation of brown compounds and α-dicarbonyl compounds through processes including Strecker degradation.^11^ The desirability of a darker, reddish cocoa powder is emphasized by elevated cocoa–chocolate flavor intensity, resulting in final products with enhanced cocoa flavor preferences.^12^

This study aims to compare the alkalizing properties of H_2_O with those of NaOH to present an alternative alkalization method in terms of green chemistry principles and to investigate the interactions and correlations between the alkalization process parameters and the antioxidant capacity, total phenolic content (TPC), phenolic profile, aroma profile, color, and pH of cocoa powders. A review of the literature in the area reveals that the aroma profile of cocoa powder obtained after the process is typically analyzed. In the present study, not only the aroma profile of the processed cocoa powder but also the aroma profile of the solution removed during the process was investigated. This approach enables the examination of odor and environmentally impactful emissions generated during the process. Additionally, a review of existing research indicates that particle size analysis, which is crucial for color comparison, is often overlooked in many studies. Therefore, standardization of the particle size was the first step of the study. Response surface methodology (RSM) was used with three factors: temperature, alkalization time, and alkali solution ratios in the final mix. Aroma compounds’ profile, pH, particle size distribution (PSD), color (L*, a*, and b*), phenolic compounds’ profile, TPC, and antioxidant capacity were determined. Optimization was performed to demonstrate whether the color parameters obtained for the NaOH-treated cocoa powder samples can also be achieved with H_2_O alkalization.

## Materials and Methods

### Reagents/Chemicals

A low-fat (10–12%, w/w) natural cocoa powder, originating from the Forastero variety from the Ivory Coast (Ulker, Istanbul, Turkey), was used in alkalization studies along with NaOH (Merck KGaA, Darmstadt, Germany) with a purity level of 99.9% as the food-grade alkalizing agent. Water was processed with Milli-Q water purification. For the determination of TPC and antioxidant capacities, gallic acid (≥98%), ethanol (≥99.8%), Folin–Ciocalteu phenol reagent, 1,1-diphenyl-2-picrylhydrazyl (DPPH), and neocuproine (Nc) from Sigma-Aldrich Chemie GmbH (Steinheim, Germany); methanol (≥99.9%), (≥98%) sodium carbonate (Na_2_CO_3_), copper(II) chloride (CuCl_2_), and ammonium acetate (NH_4_Ac) from Merck KGaA (Darmstadt, Germany); and 6-hydroxy-2,5,7,8-tetramethylchroman-2-carboxylic acid (Trolox) from Fluka Chemie (Buchs, Switzerland) were purchased.

### Study Design and Cocoa Powder

A number of variables were monitored during alkalization, including temperature, time, and the ratio of alkali solution to the final mixture. A study plan with 20 points was determined using a Central Composite Design. As a preliminary study, alkalization conditions in previous studies were reviewed.^7−16^ In a study where process times of 10, 15, 20, 25, and 30 min were considered, the relationship between the process time and cocoa powder was explored. However, the changes caused by the process time parameter in the cocoa powder were not fully elaborated, although it was noted that process time had a darkening effect on color.^7^ In our study, to reveal the changes occurring after 30 min in more detail, the process durations were chosen as 30, 50, and 70 min. Another study examined the temperature parameters at 60, 70, 80, 90, and 100 °C; however, it did not investigate antioxidant capacity and phenolic components. In our study, to explore the effects on antioxidant capacity and phenolic components, the temperature parameters were set at 40, 60, and 80 °C.^14^ Articles addressing the changes in cocoa powder due to alkalization used varying amounts of solution. In our study, based on industrial applications, the solution amount was considered a parameter while maintaining a constant molarity. A 1 M NaOH solution was used with natural cocoa powder at 30, 40, and 50% (v/w). The literature review revealed that previous studies have investigated various alkali solution quantities, temperatures, and pressures by analyzing parameters such as color, antioxidant capacity, and aroma profile. These studies primarily used cocoa liquor, cocoa nibs, and cocoa powder as the materials. The cocoa powders used were either commercially purchased or alkalized in the laboratory. In studies in which cocoa powder was alkalized in the laboratory, it was observed that one of the most critical steps in industrial production, the removal of excess moisture by vacuum, was not fully mimicked. This study aims to conduct the most accurate simulation of industrial production by using a rotary vacuum evaporator.^14−18^

In order to mimic the conventional cocoa powder alkalization process, a rotary vacuum evaporator (Buchi AG, Flawil, Switzerland) was employed in a laboratory setting. The rotary vacuum evaporator facilitated a constant volume, fixed pressure, and constant temperature with a water bath with stirring during the alkalization process. The operation of the rotary vacuum evaporator involved conducting the alkalization process at a constant temperature without activating the vacuum initially. Following alkalization, the vacuum was then activated to remove excessive moisture, resulting in an alkalized cocoa powder with a maximum moisture content of 5% at the end of the experiment.

### Sample Preparation

Following alkalization, cocoa powder particles tend to agglomerate, leading to an increase in the particle size. Given that particle size significantly influences the outcomes of color, milling and sieving (250 μm) processes were conducted on the samples to ensure that 90% of the particle size was reduced to 250 μm. A PSD analysis was conducted to determine the particle size profiles of the alkalized cocoa powder samples.

The extraction of phenolics was carried out according to the method described by Gültekin-Özgüven et al. and Wollgast.^19,20^ For defatting the samples, 1 g of the alkalized cocoa powder sample was weighed, and 10 mL of hexane was added. After 5 min of ultrasonic bath mixing at 30 °C, solutions were centrifuged at 3000 rpm for 10 min. This step was repeated twice. After defatting and centrifugation, 10 mL of methanol (80%) was added for extraction, an ultrasonic bath was applied at 30 °C for 10 min, and then the solutions were centrifuged at 3000 rpm for 10 min.

### Determination of pH, Color, and PSD

The color of the cocoa powder samples was measured with Konica Minolta, model CM-5 (Tokyo, Japan), equipped with a standard-area 3.5 in. diameter viewing port. Fifteen grams of cocoa powder were placed in a glass sample cup for color measurements. Color was measured on three scales: the L* scale measures the degree of lightness (100 = light to 0 = black), the a* scale measures red to green with true red equal to +100 and true green equal to −100, and the hunter b* scale measures yellow to blue with true yellow equal to +100 and true blue equal to −100. The main purpose of the alkalization process in terms of color is indicated as a decrease in L* and b* values and an increase in the a* value. While the L*, a*, and b* color system provides uniformity in color measurement and human perception, the color difference (Δ*E*) value is useful for determining the color difference before and after the alkalization process in the industry. Therefore, a comparison of the Δ*E* value along with the L*, a*, and b* values provides more useful information on the final color differences between the cocoa powder samples. The Δ*E* value stated in the product specifications of industrially produced alkalized cocoa powder is Δ*E* < 3. Δ*E* equation
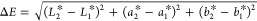


The extractable pH of the cocoa powders was determined by suspending 1 part of powder in 9 parts of deionized water at room temperature and measuring with a Mettler Toledo, model seven compact pH meter S220 (Ohio, USA), calibrated at pH 4 and 10 on the day of use. The PSD was measured using a Sympatec model quixel helos (Clausthal-Zellerfeld, Germany) that works on the laser diffraction principle. One gram of the material was dispersed in sunflower oil. The obtained mixture was then poured into a measuring cell and measured.

### Determination of Antioxidant Capacity and TPC

The total antioxidant capacity of the samples was determined using CUPRAC and DPPH assays. In both methods, the results were expressed as milligrams of Trolox equivalents (TEAC) per 100 g of dry weight (dw) sample. The copper reducing antioxidant capacity (CUPRAC) was determined^21^ as follows; 7 μL of alkalized cocoa powder extract was placed in a tube and mixed with 70 μL of 0.01 mM CuCl_2_, 70 μL of 7.5 mM neocuproine, and 70 μL of 1 M NH_4_Ac (pH 7). Immediately, 70 μL of distilled water was added to the mixture. After 30 min of incubation at room temperature in the dark, the absorbance was read at 450 nm against a reagent blank.

According to the principle of reduction of the DPPH free-radical assay, the antioxidants react with the stable DPPH radical and convert it into 1,1-diphenyl-2-picryl hydrazine.^22^ The ability to scavenge the stable DPPH radical is measured by a decrease in the absorbance. In this experimental procedure, 10 μL of the sample extract was added and was mixed with 200 μL of a 0.1 mM DPPH solution. The resultant solution was then stored in the dark at room temperature for 30 min. The absorbance of the solution was measured at a wavelength of 517 nm.

TPC was analyzed using the Folin–Ciocalteu reagent.^23^ In brief, 15 μL of the sample was carefully dispensed. Subsequently, 112.5 μL of a 0.2 N Folin reagent was introduced into the prepared sample, initiating a brief 5 min incubation period to facilitate chemical reactions. Following this interval, 112.5 μL of a 6% sodium carbonate (Na_2_CO_3_) solution was added, followed by a more extended 60 min incubation period. The absorbance of the resulting solution was subsequently measured at 765 nm, and the results were expressed in terms of milligrams of gallic acid equivalent (GAE) per 100 g of dw sample.

### Determination of Phenolic Compounds by HPLC

The phenolic compounds in the samples were evaluated by using a high-performance liquid chromatography (HPLC) system. Filtered sample extracts were analyzed by using a Waters e2695 HPLC system with a PDA detector (Waters 2998). The analysis was carried out using a Supelcosil LC-18 (25 cm × 4.60 mm, 5 m column, Sigma-Aldrich, Steinheim, Germany). In the mobile phase, Milli-Q water with 0.1% (v/v) trifluoroacetic acid (TFA) was used as solvent A, and acetonitrile with 0.1% (v/v) TFA served as solvent B. The gradient was linear as follows: At 0 min, 95% solvent A and 5% solvent B; at 45 min, 65% solvent A and 35% solvent B; at 47 min, 25% solvent A and 75% solvent B; and at 50 min, returning to the initial conditions. The flow rate was 1 mL/min. The detection wavelengths were 280, 312, and 360 nm. A characteristic UV spectrum and retention times were used to identify the specimens. External standards were used for the quantification.^24^

### Semiquantitative Analysis of Volatile Aroma Compounds with Stir-Bar Sorptive Extraction Coupled to Gas Chromatography–Mass Spectrometry (SBSE–GC–MS)

#### Sample Preparation for SBSE

In brief, 4 g of the liquid sample was weighed, and veratrole (internal standard) was added to achieve a final concentration of 5 ppm. A 4% solution was prepared and filtered for powdered samples, and 10 g of the filtrate was obtained. Veratrole was added to the filtrate to achieve a final concentration of 5 ppm. The mixture with the internal standard was stirred for 1 h using a stir bar. A PDMS-coated stir bar was placed in the mixture, and the speed was set to 600 rpm for stirring over a period of 60 min. Then the stirrer was removed from the solution using a regular magnetic fish and immersed in a beaker filled with pure water to clean the surface of the sample. Additionally, the bar was dried with a lint-free cloth to ensure no residue. The stir bar was transferred to a glass tube suitable for TDU injection, the metal cap of the device was closed, and injection into the device was performed.

#### Instrumental Analysis

Cocoa powder and cocoa distillate samples were subjected to analysis using thermal desorption unit/gas chromatography–mass spectrometry (TDU/GC–MS). The ion chromatogram for the samples was obtained through the utilization of a Gerstel TDU System (Germany) connected to an Agilent 7890A GC and Agilent 5975C MS (Triple-Axis Detector Inert MSD, USA). The SCAN method was employed in the chromatograms for the detection of volatiles present in both cocoa powders. The system incorporated a TDU system for the desorption of volatiles and a programmable temperature vaporizing injector for cryofocusing analytes before GC injection.^25^ A stir bar was placed in a TDU tray for the SBSE procedure. The desorbed compounds were automatically injected into a DB-WAX UI column (60 m × 0.25 μm film thickness × 0.25 mm inner diameter). The carrier gas, helium, flowed at a rate of 1.2 mL/min. The GC oven temperature was programmed from 40 to 240 °C at a heating rate of 5 °C/min.

#### Statistical Analysis

All analyses were performed in triplicate. The results were expressed as mean ± standard deviation, and statistical significance at *p* < 0.05 was determined by one-way ANOVA. In addition, principal component analysis (PCA), homoscedasticity tests, and main effect plot and correlation analyses were performed to elaborate the interactions related to changes in the final product due to the changes in process parameters with Minitab (State College, PA, USA) software. Trial design and optimization studies were conducted via the RSM central composite design model. Tukey’s test at a 95% significance level was performed to evaluate differences between samples.

## Results and Discussion

### Physical and Chemical Characteristics

Particle size is an important parameter for cocoa powder, affecting the color and application performance in chocolate and cocoa-related products. The milling step is required after the alkalization and vacuum process in order to achieve a homogeneous powder with a proper size distribution.^26^ The alkalization process resulted in agglomeration of cocoa powder, leading to an increase in particle size and color change between the surface and the core of the particles. The final size reduction was performed by five roller cylinders between 20 and 30 μm in chocolate production. The reduction of the particle size of the cocoa powder resulted in a color change compared to the original powder. In order to ensure a precise comparison, alkalized cocoa powder samples were ground and passed through a 250 μm sieve prior to color and PSD analyses. The particle size and pH values of H_2_O- and NaOH-treated cocoa powder samples are presented in Table 1. It was observed that the D90 values of all samples were below 250 μm.

**Table 1:** Particle Size (D90) and pH Values of H_2_O- and NaOH-Alkalized Cocoa Powder Samples^a^

				H_2_O		NaOH	
run order	temperature (°C)	alkali solution (%)	time (min)	D90	pH	D90	pH
1	60	40	50	192 ± 2^gh^	5.61 ± 0.01^bcdefB^	235 ± 3^a^	8.36 ± 0.05^eA^
2	80	50	70	209 ± 6^bc^	5.66 ± 0.02^aB^	204 ± 1^i^	9.12 ± 0.02^bA^
3	80	30	70	241 ± 5^a^	5.54 ± 0.02^iB^	185 ± 3^j^	7.60 ± 0.04^gA^
4	60	40	30	195 ± 4^efgh^	5.62 ± 0.01^bcdeB^	228 ± 2 ^bcd^	8.70 ± 0.02^cA^
5	60	40	50	207 ± 1 ^bcd^	5.58 ± 0.01^fghB^	223 ± 0^de^	8.46 ± 0.01^deA^
6	40	30	70	203 ± 3^cdef^	5.56 ± 0.02^ghiB^	190 ± 5^j^	7.82 ± 0.03^fA^
7	60	40	50	204 ± 2^bcde^	5.63 ± 0.02^bcB^	234 ± 1^ab^	8.39 ± 0.03^eA^
8	60	40	50	206 ± 3^bcd^	5.61 ± 0.01^cdefB^	230 ± 2^abcd^	8.42 ± 0.03^eA^
9	60	40	70	211 ± 2^bc^	5.59 ± 0.01^defgB^	204 ± 2^hi^	8.47 ± 0.03^deA^
10	80	40	50	196 ± 2^efgh^	5.60 ± 0.01^cdefB^	216 ± 1^fg^	8.33 ± 0.02^eA^
11	80	30	30	207 ± 1^bcd^	5.55 ± 0.01^hiB^	204 ± 2^hi^	7.37 ± 0.02^hA^
12	60	50	50	198 ± 4^defg^	5.63 ± 0.01^abcB^	211 ± 2^gh^	9.25 ± 0.23^bA^
13	40	50	70	206 ± 1^bcd^	5.63 ± 0.01^abcB^	223 ± 1^cde^	9.50 ± 0.02^aA^
14	40	50	30	187 ± 5^h^	5.65 ± 0.01^abB^	228 ± 2^bcd^	9.62 ± 0.03^aA^
15	40	30	30	204 ± 1^bcde^	5.56 ± 0.02^ghiB^	203 ± 2^i^	7.63 ± 0.03^gA^
16	60	40	50	194 ± 1^fgh^	5.62 ± 0.01^bcdB^	231 ± 2^ab^	8.42 ± 0.01^eA^
17	60	40	50	213 ± 1^b^	5.59 ± 0.02^efgB^	230 ± 3^abc^	8.38 ± 0.01^eA^
18	80	50	30	167 ± 2^i^	5.64 ± 0.01^abB^	221 ± 2^ef^	9.29 ± 0.02^bA^
19	40	40	50	206 ± 0^bcd^	5.58 ± 0.01^fghB^	204 ± 1^i^	8.62 ± 0.02^cdA^
20	60	30	50	212 ± 5^bc^	5.53 ± 0.02^iB^	204 ± 2^hi^	7.50 ± 0.05^ghA^

a Values in the same column followed by different lowercase letters are significantly different (*p* < 0.05). Values in the same row followed by different uppercase letters are significantly different (*p* < 0.05).

pH is another significant parameter defining the quality of alkalized cocoa powders and has an important impact on the end product in which the cocoa is used. The pH value of natural cocoa powder used in alkalization was measured as 5.48 ± 0.01, which has been reported to be between 5.2 and 5.6 for natural cocoa powder.^27^ The ratio of H_2_O or NaOH solution used in alkalization was observed to have a significant effect on the pH of alkalized cocoa samples (*p* < 0.05). The pH values of H_2_O-treated cocoa powder samples were found to be between 5.55 and 5.66, which was within the pH range of natural cocoa powder. NaOH has the highest neutralization potential within the permissible alkalis; accordingly, the pH values of NaOH-treated cocoa powder samples were measured to be between 7.37 and 9.60. The end product of the alkalization process is cocoa powder with a higher pH and a darker color compared to natural cocoa powder. The color properties are significant parameters used for the definition and classification of cocoa powders.

The color parameters of H_2_O- and NaOH-treated cocoa powder samples are provided in Table 2. The color parameters of natural cocoa powder samples were measured (L* = 49.99 ± 0.01; a* = 11.94 ± 0.01; b* = 22.99 ± 0.03) as a reference. Using H_2_O in alkalization resulted in Δ*E* values between 3.36 and 11.89, indicating a significant color change in the cocoa powder after the alkalization process. Besides, a significant decrease in the L* value was observed after the alkalization process compared to the natural cocoa powder in H_2_O-treated samples (*p* < 0.05).

**Table 2:** Color Measurement Results of H_2_O- and NaOH-Treated Cocoa Powder Samples in Terms of L*, a*, b*, and Δ*E* Values^a^

	H_2_O				NaOH			
run order	L*	a*	b*	Δ*E*	L*	a*	b*	Δ*E*
1	43.33 ± 0.01^fA^	12.09 ± 0.02^ghB^	21.27 ± 0.02^fA^	6.88 ± 0.01^gB^	35.16 ± 0.02^gB^	12.83 ± 0.02^dA^	18.77 ± 0.02^jB^	15.4 ± 0.02^jA^
2	41.86 ± 0.01^iA^	12.47 ± 0.02^eA^	18.62 ± 0.05^iA^	9.25 ± 0.02^dB^	28.46 ± 0.03^qB^	12.01 ± 0.03^gB^	13.60 ± 0.03^rB^	23.5 ± 0.02^rA^
3	39.12 ± 0.01^lA^	11.29 ± 0.01^lB^	18.23 ± 0.03^lA^	11.89 ± 0.01^bB^	31.24 ± 0.04^mB^	13.28 ± 0.04^bA^	16.41 ± 0.04^oB^	19.9 ± 0.03^oA^
4	45.56 ± 0.01^cA^	12.61 ± 0.01^dB^	22.29 ± 0.01^cA^	4.54 ± 0.01^jB^	36.67 ± 0.02^dB^	13.20 ± 0.02^bA^	19.02 ± 0.02^gB^	15.0 ± 0.02^gA^
5	43.43 ± 0.01^fA^	12.14 ± 0.01^fgB^	21.29 ± 0.05^fA^	6.78 ± 0.01^gB^	35.49 ± 0.02^eB^	12.85 ± 0.02^dA^	18.42 ± 0.02^lB^	15.2 ± 0.01^lA^
6	45.11 ± 0.04^dA^	12.65 ± 0.02^dB^	21.92 ± 0.01^dB^	5.04 ± 0.04^iB^	40.86 ± 0.02^bB^	12.89 ± 0.02^dA^	22.72 ± 0.02^aA^	9.2 ± 0.02^aA^
7	43.32 ± 0.01^fA^	12.06 ± 0.01^hiB^	21.24 ± 0.01^fA^	6.90 ± 0.01^gB^	35.11 ± 0.02^gB^	12.86 ± 0.02^dA^	18.56 ± 0.02^jkB^	15.6 ± 0.01^jklA^
8	43.42 ± 0.01^fA^	11.98 ± 0.01^jB^	21.22 ± 0.01^fA^	6.81 ± 0.01^gB^	34.93 ± 0.02^iB^	12.84 ± 0.02^dA^	18.62 ± 0.02^ijB^	15.7 ± 0.01^ijA^
9	45.15 ± 0.20^dA^	12.13 ± 0.04^fgB^	21.26 ± 0.18^dA^	5.15 ± 0.25^iB^	33.40 ± 0.03^lB^	12.39 ± 0.03^eA^	18.40 ± 0.03^lB^	17.2 ± 0.03^lA^
10	38.95 ± 0.01^mA^	11.17 ± 0.03^mB^	17.82 ± 0.02^mA^	12.21 ± 0.01^aB^	34.05 ± 0.02^kB^	12.24 ± 0.02^fA^	18.18 ± 0.02^fB^	16.4 ± 0.02^fA^
11	39.91 ± 0.01^kA^	11.69 ± 0.02^kB^	19.37 ± 0.03^kB^	10.72 ± 0.01^cB^	34.74 ± 0.01^jB^	13.06 ± 0.01^cA^	19.47 ± 0.01^eA^	15.7 ± 0.01^eA^
12	45.22 ± 0.02^dA^	12.03 ± 0.01^ijA^	19.07 ± 0.02^dA^	6.18 ± 0.00^hB^	27.31 ± 0.01^rB^	11.76 ± 0.01^hB^	14.44 ± 0.01^pB^	24.2 ± 0.02^pA^
13	46.00 ± 0.01^bA^	13.44 ± 0.02^aA^	24.31 ± 0.02^bA^	4.47 ± 0.01^jB^	31.01 ± 0.01^oB^	12.48 ± 0.01^eB^	16.96 ± 0.01^nB^	19.9 ± 0.02^nA^
14	46.71 ± 0.01^aA^	12.63 ± 0.01^dA^	22.77 ± 0.01^aA^	3.36 ± 0.01^kB^	31.14 ± 0.03^nB^	11.45 ± 0.03^iB^	17.13 ± 0.03^mB^	19.7 ± 0.03^mA^
15	43.78 ± 0.01^eB^	13.17 ± 0.02^bA^	22.94 ± 0.01^eA^	6.34 ± 0.01^hB^	44.06 ± 0.01^aA^	11.86 ± 0.01^hB^	21.80 ± 0.01^bB^	6.1 ± 0.00^bA^
16	43.38 ± 0.02^fA^	12.03 ± 0.01^iB^	21.17 ± 0.02^fA^	6.86 ± 0.02^gB^	35.03 ± 0.03^hB^	12.86 ± 0.03^dA^	18.48 ± 0.03^klB^	15.7 ± 0.03^klA^
17	43.40 ± 0.05^fA^	12.12 ± 0.01^fgB^	21.19 ± 0.04^fA^	6.84 ± 0.05^gB^	34.96 ± 0.01^iB^	12.81 ± 0.01^dA^	18.69 ± 0.01^hiB^	15.7 ± 0.01^hiA^
18	42.04 ± 0.01^hA^	11.28 ± 0.01^lA^	18.96 ± 0.02^hA^	8.94 ± 0.01^eB^	28.63 ± 0.03^pB^	11.08 ± 0.03^jB^	13.94 ± 0.03^qB^	23.2 ± 0.02^qA^
19	42.77 ± 0.02^gA^	13.01 ± 0.01^cA^	22.43 ± 0.02^gA^	7.33 ± 0.02^fB^	35.32 ± 0.01^fB^	12.42 ± 0.01^eB^	19.74 ± 0.01^dB^	15.0 ± 0.01^dA^
20	41.44 ± 0.01^jA^	12.17 ± 0.03^fB^	19.82 ± 0.02^jB^	9.12 ± 0.01^dB^	37.01 ± 0.01^cB^	13.40 ± 0.01^aA^	20.97 ± 0.01^cB^	13.2 ± 0.01^cA^

a Values in the same column followed by different lowercase letters are significantly different (*p* < 0.05). Values in the same row followed by different uppercase letters are significantly different (*p* < 0.05).

**Figure 1:**
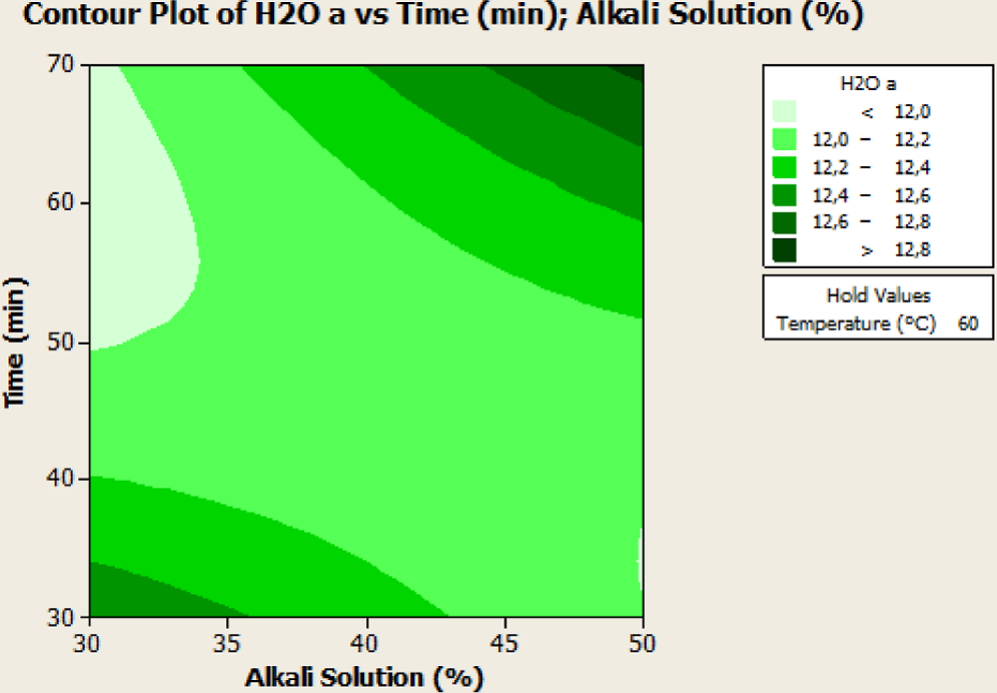
Contour plots indicating the effects of time and alkali solution interaction on the a* values of H_2_O-treated cocoa powder samples.

In H_2_O-treated samples, the L* values were significantly affected by the temperature and alkali solution ratio (*p* < 0.05), whereas the a* and b* values were affected only by temperature (*p* < 0.05). When the interactions of process parameters for H_2_O-treated samples were examined, it was observed that the alkali solution ratio and time interaction were significant (*p* < 0.05) (Figure 1). The a*/b* ratio is considered to be a significant parameter for indicating a reddish hue in alkalized cocoa powder. Therefore, a high a* value postprocessing is crucial for color evaluation. Examining the interaction between the solution amount and processing time revealed that an increase in both parameters leads to a higher a* value. It can be concluded that the increase in processing time and the amount of free water in the environment positively impact the reactions that form compounds, supporting the a* value. The Δ*E* values were observed to be linearly affected by the temperature and alkali solution ratio. This interaction can be explained by the main change in color caused by the L* value. Correlation tests with color and process parameters indicated that temperature had a significant negative correlation with the L*, a*, and b* values (*p* < 0.05).

**Figure 2:**
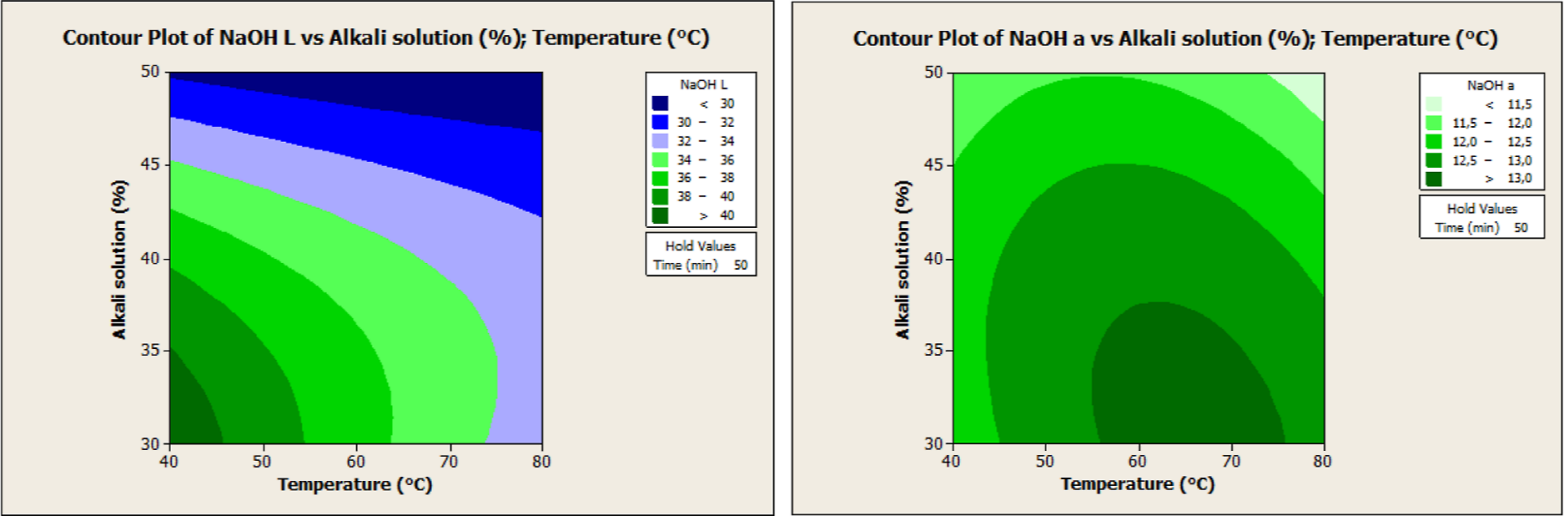
Contour plots indicate the effects of process parameters on the L* and a* values of NaOH-treated samples.

The alkali solution ratio and temperature are critical parameters for color improvement during alkalization. Higher alkali solution ratios and higher temperatures accelerate the oxidation of phenolics into quinones, which results in the formation of high-molecular-weight dark compounds.^7^L* values of NaOH-alkalized samples were significantly affected by all process parameters, whereas a* values were affected by the alkali solution ratio and time (*p* < 0.05). It was also observed that the temperature and alkali solution ratio significantly changed b* values (*p* < 0.05). Due to the changes in L*, a*, and b* values, the Δ*E* values of NaOH-alkalized samples were significantly affected by all process parameters (*p* < 0.05). When the interactions between process parameters were examined, the temperature and alkali solution ratio interaction was found to be significant for L* and a* values for NaOH-treated cocoa powder samples. This interaction can be explained by the increase in Maillard reactions, which is facilitated by the presence of more free water and an increase in the environmental pH, enhancing the efficiency of the reaction mechanism. The effects of the process parameters on the color of NaOH-treated cocoa powder samples are shown as contour plots in Figure 2. A significant positive correlation between the pH and Δ*E* values of NaOH-treated samples was found (*p* < 0.05). This correlation provides a clear link between the pH and color parameters. A correlation test with color and process parameters was conducted, and the results revealed that temperature had a significant negative correlation with the L*, and b* values, whereas the alkali solution ratio had a significant negative correlation on all color parameters (*p* < 0.05).

**Figure 3:**
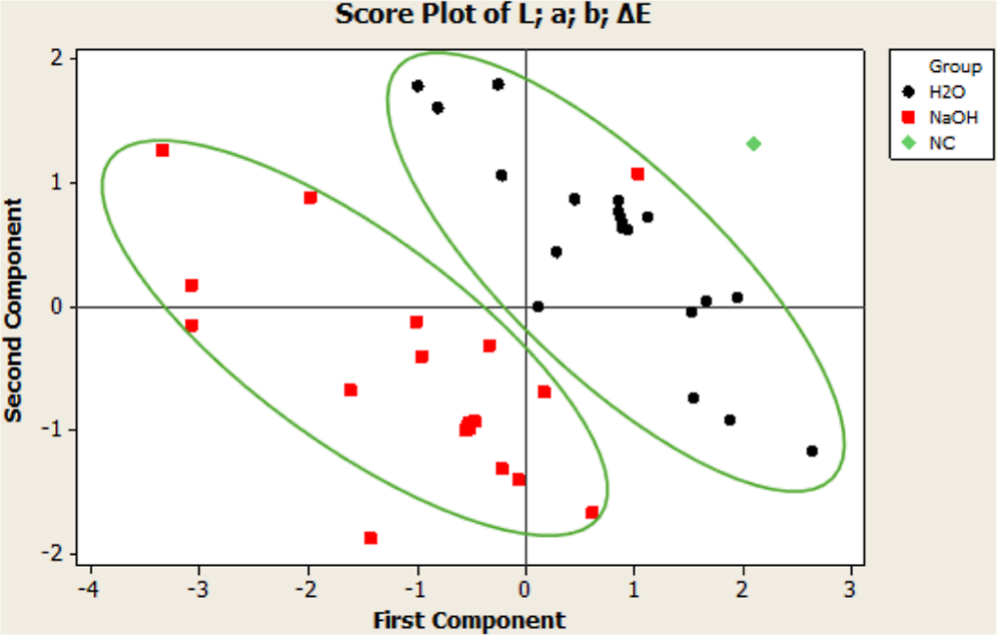
PCA plot of L*, a*, b*, and Δ*E* values of H_2_O-treated and NaOH-treated cocoa powder samples.

The H_2_O ratio was observed to have a significant effect only on the L* value, while the NaOH ratio affected the L*, a*, and b* values. The reactions that are responsible for color change were influenced by changes in pH and temperature.^7^ There was a precise interaction between the alkalizing solution type and L* values in such a way that a 6.6–22.1% decrease in L* values was observed in H_2_O-treated samples, whereas the L* values of NaOH-treated samples decreased by 11.9–45.4%. PCA plots studied with L*, a*, b*, and Δ*E* values aim to identify clusters of samples that are close to each other (Figure 3). The eigenvalue analysis of the correlation matrix in PCA reveals that the first two principal components (PC1 and PC2) account for 82.2% of the total variance, indicating that much of the data set can be represented by these two components. PC1 is significantly influenced by variables L* and b*, with loadings of 0.637 and 0.628, respectively, while PC2 is particularly influenced by variable a*, with a loading of −0.861. PC3 and PC4 explain only a small portion of the total variance (16.3 and 1.5%, respectively), with lower contributions. This analysis demonstrates the strong influence of the L* and b* variables on PC1 and the significant effect of the a* variable on PC2. Consequently, utilizing only the first two principal components is sufficient to comprehend and interpret the fundamental patterns in the data set, thereby simplifying the analysis and modeling process.

The color change is also linked to the oxidation reactions which involve proteins, phenolics, and some minor compounds.^13^ It was reported that an increase in pH affects the enzyme activities in alkalization. Polyphenol oxidase (PPO) which is responsible for brownish color development was indicated to be affected by pH and showed optimal activity at pH 8.0.^28^ Thus, it can be indicated that a higher decrease in L* values was found at higher pH values observed in the NaOH-treated cocoa powder samples.

Nonenzymatic browning reactions also occur during alkalization. In the case of H_2_O alkalization, pH did not increase sufficiently for PPO activation; therefore, the color change was due to nonenzymatic reactions, such as caramelization and Maillard reactions. On the other hand, it was reported that the optimum pH for PPO is 6.5 and it is stable between pH 5–11.^29^ Therefore, it can be concluded that PPO activity contributes to color development for the H_2_O-treated cocoa powder samples. It was indicated that heat treatment applied to the food matrix positively influences the nonenzymatic browning reactions.^30^ It was reported that there was no significant correlation between water activity and color change.^26^ A positive weak correlation was present between the H_2_O ratio and L* values, whereas a significant negative correlation was found between temperature and L* values in H_2_O-treated samples (*p* < 0.05).

**Figure 4:**
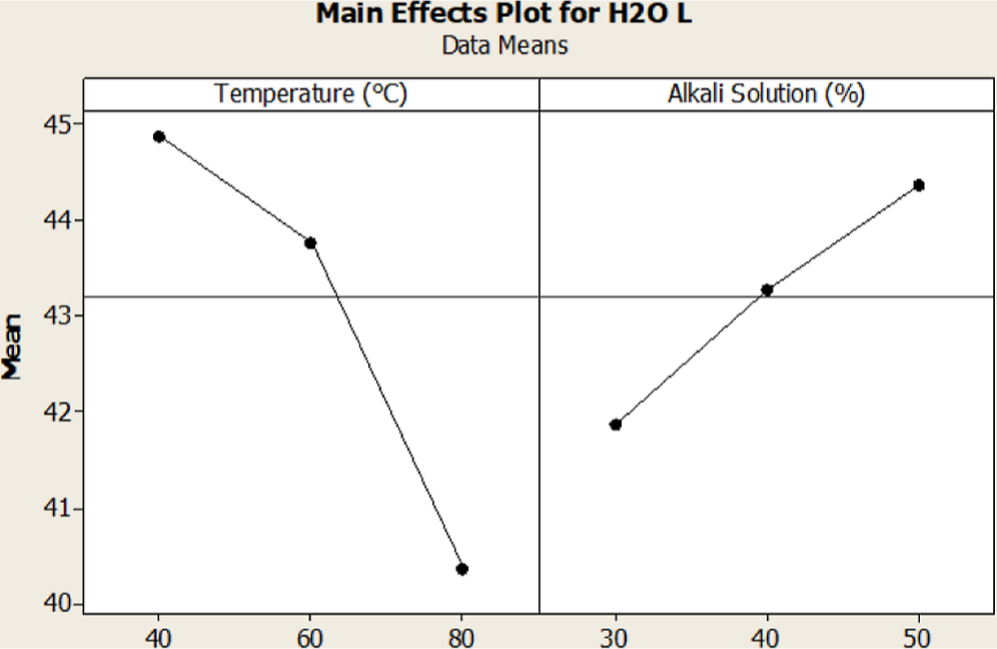
Main effect plot for H_2_O-treated cocoa powder samples in terms of the L* values.

When the temperature and time were constant, a higher free water content provided a suitable matrix for PPO activity. However, the dilution of the matrix also caused a decrease in enzymatic and nonenzymatic browning reactions. Increasing the temperature reduced the L* values, resulting in darker cocoa powder. The deactivation temperature for PPO is indicated to start at 65 °C and can change depending on the process time.^31^ While the increase in temperature decreases PPO activity, it contributes to nonenzymatic reactions. In this context, the changes in L* values reveal that the nonenzymatic browning reactions had the main effect on color change for H_2_O-treated samples at higher temperatures. It was observed that the effect of PPO activities on color change had more influence compared to the nonenzymatic browning (Figure 4).

### Antioxidant Capacity, TPC, and Phenolic Profile Analysis

The antioxidant activity of cocoa powders, as assessed by the DPPH and CUPRAC assays, varied within a wide range for natural, H_2_O-treated, and NaOH-treated cocoa powders. Natural cocoa powder’s antioxidant capacity was found as 311.6 ± 2.6 TEAC mg/100 g according to the DPPH method. In the case of the alkalized cocoa powder samples, the DPPH antioxidant capacity ranged between 295.5 and 317.7 TEAC mg/100 g and between 256.6 and 306.2 TEAC mg/100 g for the H_2_O-treated and NaOH-treated samples, respectively. The antioxidant capacity of the natural cocoa powder determined by the CUPRAC method was 1370 TEAC mg/100 g. In the case of the alkalized cocoa powder samples, the CUPRAC antioxidant capacity changed between 835 and 1544 TEAC mg/100 g and between 148 and 849 TEAC mg/100 g for the H_2_O-treated and NaOH-treated samples, respectively. Following H_2_O alkalization, antioxidant activity was not significantly impacted, as demonstrated by both DPPH and CUPRAC tests, indicating that this mild alkalization process may not have a significant impact on the antioxidant capacity of cocoa powder (*p* > 0.05). This can be explained by the preservation of a significant number of phenolic compounds responsible for antioxidant activity during H_2_O alkalization. In contrast, a significant decrease in antioxidant activity was observed after NaOH alkalization, as shown by both the DPPH and CUPRAC assays, indicating that more intensive NaOH alkalization had a pronounced effect on cocoa powder’s antioxidant properties (*p* < 0.05). The alkalization of cocoa powder with NaOH may contribute to the degradation or alteration of phenolic compounds, resulting in a reduction in antioxidant activity.

In line with previous studies, these findings indicated that alkalization significantly impacts the TPC and antioxidant activities of cocoa products. Alkalization of cocoa powder, particularly with NaOH, has been associated with a decrease in particular phenolic compounds and a reduction in antioxidant capacity, possibly explaining the significant reduction in antioxidant activity observed in the alkalized cocoa powder.^16−32^ Accordingly, the results indicate that the degree of alkalization, with NaOH exerting greater pronounced effects compared with H_2_O alkalization, plays a crucial role in determining the antioxidant activity of cocoa powders. It is critical to consider the impacts of the alkalization process in the manufacture of cocoa products to preserve their phenolic content and related health benefits.

DPPH and CUPRAC methods provided varying results for the antioxidant capacity of the samples, where relatively higher values were obtained with the CUPRAC method compared to the DPPH method (Table 3). Several factors related to the measurement principles of the methods may contribute to these differences, including the diversity of reaction conditions, wavelengths, and durations of the measurements.^33,34^ Previously published studies have shown that the chromogens of the CUPRAC method are well soluble in both organic and aqueous solvent systems,^35^ which may explain the relatively higher values obtained with this method. The differences in the measurement results obtained with different methods are mainly related to the nonstandardized assay techniques, different radicals that are generated, the duration of the reaction, or the mechanisms involved. Therefore, it has been indicated that two or more analysis procedures should be applied for a complete evaluation of the antioxidant activity.^32−37^

**Table 3:** Antioxidant Capacity and TPC of H_2_O- and NaOH-Treated Cocoa Powder Samples^a^

	H_2_O-treated samples			NaOH-treated samples		
run order	DPPH (mgTEAC/100 g)	CUPRAC (mgTEAC/100 g)	total phenolic (mgGAE/100 g)	DPPH (mgTEAC/100 g)	CUPRAC (mgTEAC/100 g)	total phenolic (mgGAE/100 g)
1	316.3 ± 2.6^abA^	1014 ± 113^ghiA^	297.8 ± 4.3^bcdA^	284.4 ± 4.9^defB^	836 ± 109^aB^	194.4 ± 22.7^cB^
2	312.1 ± 3.2^abcdeA^	1519 ± 200^bcA^	297.8 ± 3.3^bcdA^	271.5 ± 3.2^gB^	394 ± 43^efB^	95.9 ± 14.2^hB^
3	317.7 ± 9.7^aA^	1126 ± 84^defghA^	287.5 ± 15.5^bcdA^	302.7 ± 1.7^abB^	467 ± 52^cdefB^	169.5 ± 12.3^cdB^
4	303.7 ± 5.3^defA^	1542 ± 134^bA^	321.6 ± 15.5^aA^	274.5 ± 5.6^fgB^	709 ± 117^bB^	268.7 ± 20.4^abB^
5	313.9 ± 1.3^abcA^	1073 ± 158^fghA^	292.4 ± 17.8^bcdA^	282.1 ± 7.3^efB^	849 ± 44^aB^	177.5 ± 18.5^cdB^
6	302.3 ± 0.2^efB^	1297 ± 130^cdefA^	293.3 ± 20.8^bcdA^	306.2 ± 1.7^aA^	584 ± 92^cB^	290.2 ± 26.6^abA^
7	316.4 ± 5.6^abA^	909 ± 91^hiA^	281.3 ± 9.6^dA^	286.2 ± 8.3^cdeB^	841 ± 54^aA^	175.6 ± 18.7^cdB^
8	315.9 ± 1.5^abA^	1084 ± 88^efghA^	291.4 ± 13.7^bcdA^	283.6 ± 9.7^defB^	837 ± 69^aB^	185.1 ± 20.8^cB^
9	316.4 ± 5.6^abA^	1809 ± 189^aA^	296.7 ± 9.4^bcdA^	295.0 ± 7.4^bcB^	369 ± 37^fgB^	165.1 ± 12.9^cdeB^
10	316.8 ± 5.6^abA^	1467 ± 105^bcA^	298.5 ± 5.2^bcdA^	297.7 ± 5.0^abB^	445 ± 59^defB^	146.9 ± 22.10^defB^
11	310.0 ± 0.0^abcdeA^	1100 ± 77^defghA^	296.5 ± 13.9^bcdA^	303.9 ± 3.9^abB^	547 ± 67^cdB^	259.6 ± 24.1^bB^
12	312.5 ± 4.2^abcdA^	1188 ± 69^defgA^	301.9 ± 4.4^abcdA^	256.6 ± 5.3^hB^	261 ± 23^ghB^	100.0 ± 18.1^hB^
13	302.3 ± 0.2^efA^	1314 ± 185^bcdeA^	297.7 ± 8.06^bcdA^	280.2 ± 6.4^efgB^	171 ± 22^hB^	111.4 ± 17.1^ghB^
14	295.5 ± 4.5^fA^	1321 ± 105^bcdA^	307.0 ± 5.2^abA^	281.1 ± 4.8^efgB^	190 ± 32^hB^	125.8 ± 21.1^fghB^
15	307.1 ± 5.7^bcdeA^	1468 ± 207^bcA^	301.9 ± 10.7^abcdA^	293.8 ± 6.3^bcdB^	414 ± 47^efB^	298.6 ± 17.2^aA^
16	310.6 ± 6.0^abcdeA^	997 ± 59^ghiA^	292.3 ± 16.7^bcdA^	286.4 ± 7.2^cdeB^	834 ± 55^aB^	164.1 ± 19.2^cdeB^
17	302.7 ± 1.2^defA^	972 ± 167^ghiA^	305.2 ± 10.1^abcA^	284.5 ± 6.1^defB^	828 ± 68^aB^	168.8 ± 14.2^cdeB^
18	305.2 ± 5.5^cdefA^	1544 ± 249^bA^	288.3 ± 9.7^bcdA^	258.3 ± 8.7^hB^	263 ± 30^ghB^	136.1 ± 19.2^efgB^
19	305.6 ± 5.5^cdeA^	963 ± 147^ghiA^	284.4 ± 15.7^cdA^	281.9 ± 8.5^efB^	507 ± 61^cdeB^	272.4 ± 19.2^abA^
20	310.4 ± 0.0^abcdeA^	835 ± 157^iA^	284.9 ± 11.5^bcdA^	297.8 ± 2.9^abB^	848 ± 152^aA^	280.7 ± 25.2^abA^

a Values in the same column followed by different lowercase letters are significantly different (*p* < 0.05). Values in the same row followed by different uppercase letters are significantly different (*p* < 0.05).

The phenolic compounds present in cocoa powder are mainly responsible for color development through alkalization and roasting processes. Our results revealed that the color and phenolic content can be adjusted via alkalization process parameters (Table 3). Natural cocoa powder’s TPC was found as 363.8 ± 45.3 mg GAE/100 g sample. A significant reduction in TPC was observed after alkalization of cocoa powders by H_2_O (281.3–321.6 mg GAE/100 g; *p* < 0.05). Still, a more pronounced reduction was observed after the alkalization by NaOH (95.9–298.6 mg GAE/100 g; *p* < 0.05), suggesting that the impact of different alkalization processes on the phenolic content of the cocoa powder varies.

Despite the fact that H_2_O alkalization decreased the phenolic content of cocoa powder, the effect was less severe than that caused by NaOH alkalization. This milder reduction in the phenolic content may be attributed to the milder conditions of H_2_O alkalization compared to NaOH alkalization. The moderate decline in TPC is likely to result from H_2_O alkalization, which preserves a relatively higher proportion of phenolic compounds in the cocoa powder. On the other hand, more substantial reductions observed after NaOH alkalization indicate more significant degradation or alteration of phenolic compounds as a result of the stronger alkalizing agent. These findings support previous studies which indicate that alkalization processes using NaOH result in reductions in the phenolic content of cocoa products.^16−39^ As a result of these findings, the effects of different alkalization methods on the phenolic content of cocoa products should be monitored closely in order to preserve their antioxidant properties and associated health benefits. A significant correlation was found between the DPPH antioxidant capacity and TPC of NaOH-treated cocoa powders (*r* = 0.542, *p* < 0.05). Moreover, a strong positive correlation was observed between the CUPRAC antioxidant capacity and phenolic profile of NaOH-treated samples (*r* = 0.749, *p* < 0.05). In the case of H_2_O-treated samples, the antioxidant capacities in terms of both DPPH and CUPRAC methods were observed to be correlated with the phenolic profile. Moreover, a significant correlation was found between the TPC and phenolic profile of H_2_O-treated samples (*r* = 0.480, *p* < 0.05). In the correlation study, the phenolic amounts detected by phenolic profile analysis were calculated and the study was carried out on the total value.

Individual phenolic profiles differed between NaOH-treated and H_2_O-treated cocoa powders, suggesting that the alkalization process impacted phenolic compounds differently (Table 4). In comparison to the H_2_O-treated cocoa powder, the amounts of protocatechuic acid, epigallocatechin gallate, vanillic acid, *p*-coumaric acid, *trans*-resveratrol, quercetin-3-*O*-galactoside, and quercetin-3-*O*-glucoside were significantly higher in NaOH-treated cocoa powder (*p* < 0.05), thereby suggesting that NaOH alkalization preserved or enhanced these specific phenolic compounds. In contrast, the higher levels of catechin, epicatechin, caffeic acid, ferulic acid, and quercetin found in H_2_O-treated cocoa powder suggest that these compounds have been retained or generated more during the H_2_O alkalization process than with NaOH (*p* < 0.05). Chemical reactions and conditions involved in each process may contribute to the differences in phenolic compound profiles among the two alkalization methods applied. Accordingly, these findings were in accordance with the complex nature of phenolic compounds and how they respond to different processing conditions.^40^ In some cases, alkalization, especially with NaOH, may lead to the degradation of certain phenolic compounds, whereas in others, the process may lead to increased stability or even enhancement of the compounds. Based on the results, it is important to consider the specific impacts of different alkalization methods on the different phenolic compounds in cocoa powder. An understanding of these variations is necessary for optimizing processing techniques in order to increase the levels of beneficial phenolic compounds in cocoa products.

**Table 4:** Phenolic Profile of H_2_O- and NaOH-Treated Cocoa Powder Samples (Milligrams of Compound per 100 g of Cocoa Powder)^a^

RO	alkaline type	protocatechuic acid	catechin	(−)-epicatechin and derivatives	(−)-epigallocatechin gallate and derivatives	vanillic acid	caffeic acid	p-coumaric acid	ferulic acid	trans-resveratrol	quercetin-3-*O*-galactoside	quercetin-3-*O*-glucoside	quercetin
1	H_2_O	5.90^k***B***^	13.62^ghi***A***^	89.05^hi***A***^	116.7^gh***B***^	0.27^h***B***^	12.82^ij***A***^	2.52^gh***B***^	1.30^hıj***A***^	1.27^hi***B***^	0.38^ef***B***^	0.08^cde***B***^	0.42^def***A***^
	NaOH	13.42^BCD***A***^	10.55^EF***B***^	52.1^EF***B***^	196.9^D***A***^	0.66^D***A***^	11.56^EF***B***^	3.95^A***A***^	0.97^FG***B***^	2.25^A***A***^	0.44^ABC***A***^	0.15^A***A***^	0.16^CDE***B***^
2	H_2_O	12.20^cd***A***^	16.57^ef***A***^	92.12^ghi***A***^	132.7^de***B***^	0.41^efg***B***^	14.64^fgh***A***^	3.01^ef***A***^	1.70^def***A***^	1.64^de***A***^	0.41^de***A***^	0.08^cdef***A***^	0.42^def***A***^
	NaOH	13.55^ABCD***A***^	8.53^GH***B***^	40.7^J***B***^	178.6^E***A***^	0.59^FG***A***^	4.63^N***B***^	2.89^DE***B***^	0.59^J***B***^	1.58^DEF***B***^	0.22^GH***B***^	0.07^FGH***A***^	0.05^E***B***^
3	H_2_O	10.70^def***B***^	13.69^ghi***A***^	74.81^kl***A***^	114.5^gh***B***^	0.37^efgh***B***^	12.15^jkl***A***^	2.53^gh***A***^	1.40^ghi***A***^	1.35^gh***B***^	0.26^h***B***^	0.05^g***B***^	0.40^def***A***^
	NaOH	12.33^E***A***^	8.75^GH***B***^	47.9^FGHI***B***^	163.8^H***A***^	0.52^I***A***^	9.91^G***B***^	2.56^FGH***A***^	1.08^D***B***^	1.65^CDEF***A***^	0.33^EF***A***^	0.08^DEFG***A***^	0.21^BCD***B***^
4	H_2_O	14.70^b***A***^	24.13^a***A***^	154.0^b***A***^	180.4^b***B***^	1.20^a***A***^	20.78^ab***A***^	4.17^b***A***^	2.45^a***A***^	2.24^b***B***^	0.50^bc***A***^	0.12^b***A***^	0.54^bc***A***^
	NaOH	13.10^CD***B***^	10.72^E***B***^	56.5^DE***B***^	202.4^AB***A***^	0.73^A***B***^	12.04^C***B***^	3.62^A***B***^	1.02^E***B***^	2.34^A***A***^	0.45^ABC***B***^	0.09^BCDEF***B***^	0.14^CDE***B***^
5	H_2_O	10.90^de***B***^	16.88^def***A***^	104.2^ef***A***^	127.6^efg***B***^	0.37^fgh***B***^	14.33^ghi***A***^	2.89^f***A***^	1.70^def***A***^	1.56^ef***B***^	0.36^efg***B***^	0.08^cdef***B***^	0.42^def***A***^
	NaOH	13.15^BCD***A***^	10.28^F***B***^	51.0^FG***B***^	199.8^C***A***^	0.67^BC***A***^	11.73^D***B***^	2.49^GH***B***^	0.99^F***B***^	1.79^BCDE***A***^	0.49^AB***A***^	0.15^A***A***^	0.17^CDE***B***^
6	H_2_O	12.60^c***A***^	21.75^b***A***^	170.6^a***A***^	162.9^c***A***^	0.84^c***A***^	18.81^cd***A***^	3.75^c***A***^	2.22^b***A***^	2.03^c***A***^	0.45^cd***B***^	0.09^cd***A***^	0.60^b***A***^
	NaOH	6.31^I***B***^	11.31^D***B***^	58.2^D***B***^	110.2^L***B***^	0.31^N***B***^	8.59^J***B***^	2.07^I***B***^	0.97^FG***B***^	1.27^F***B***^	0.53^A***A***^	0.05^H***B***^	0.20^A***B***^
7	H_2_O	6.76^jk***B***^	11.38^jk***A***^	79.5^jk***A***^	96.1^i***B***^	0.30^fgh***B***^	10.33^m***B***^	2.14^i***B***^	1.16^j***A***^	1.16^i***B***^	0.31^gh***B***^	0.06^efg***B***^	0.31^gh***A***^
	NaOH	13.65^ABC***A***^	10.36^EF***B***^	47.1^GHI***B***^	203.7^A***A***^	0.68^B***A***^	11.60^E***A***^	2.43^H***A***^	0.95^G***B***^	2.31^A***A***^	0.52^A***A***^	0.16^A***A***^	0.17^CDE***B***^
8	H_2_O	7.77^hij***B***^	10.07^k***B***^	74.7^kl***A***^	111.5^h***B***^	0.34^fgh***B***^	12.66^ijk***A***^	2.57^gh***B***^	1.44^gh***A***^	1.36^gh***B***^	0.34^fg***B***^	0.07^cdefg***B***^	0.34^fgh***A***^
	NaOH	13.44^BCD***A***^	10.40^EF***A***^	49.2^FGH***B***^	201.3^ABC***A***^	0.67^BC***A***^	11.61^E***B***^	2.70^EFG***A***^	0.96^FG***B***^	2.29^A***A***^	0.50^AB***A***^	0.15^A***A***^	0.17^CDE***B***^
9	H_2_O	8.33^hi***B***^	13.29^hij***A***^	94.4^ghi***A***^	118.4^fgh***A***^	0.37^fgh***B***^	13.48^ghij***A***^	2.72^fg***B***^	1.60^efg***A***^	1.45^fg***B***^	0.41^de***A***^	0.08^cde***A***^	0.39^efgh***A***^
	NaOH	11.36^F***A***^	6.83^K***B***^	45.8^HI***B***^	166.2^H***B***^	0.58^G***A***^	8.74^I***B***^	2.98^CD***A***^	0.62^I***B***^	1.86^BCD***A***^	0.38^CDE***B***^	0.09^BCD***A***^	0.10^DE***B***^
10	H_2_O	9.06^gh***B***^	11.99^ijk***A***^	84.8^ij***A***^	116.5^gh***B***^	0.38^efgh***B***^	13.30^hij***A***^	2.73^fg***B***^	1.52^fg***A***^	1.45^fg***B***^	0.37^efg***A***^	0.07^cdefg***A***^	0.40^efg***A***^
	NaOH	14.05^A***A***^	8.43^H***B***^	31.0^K***B***^	170.4^G***A***^	0.60^EF***A***^	9.52^H***B***^	2.91^DE***A***^	0.69^H***B***^	1.85^BCD***A***^	0.33^EF***B***^	0.08^CDEFG***A***^	0.10^DE***B***^
11	H_2_O	7.26^ijk***B***^	10.42^k***B***^	70.0^l***A***^	96.9^i***B***^	0.31^fgh***B***^	10.64^lm***B***^	2.19^i***B***^	1.22^ij***B***^	1.16^i***B***^	0.31^gh***B***^	0.05^fg***B***^	0.31^gh***A***^
	NaOH	13.04^D***A***^	12.72^B***A***^	63.6^C***B***^	148.3^K***A***^	0.46^J***A***^	11.66^DE***A***^	2.71^EFG***A***^	1.31^C***A***^	1.65^CDEF***A***^	0.37^CDE***A***^	0.10^B***A***^	0.27^ABC***B***^
12	H_2_O	8.91^gh***B***^	14.26^gh***A***^	99.1^fg***A***^	126.6^efg***B***^	0.41^ef***B***^	13.91^ghi***A***^	2.79^fg***B***^	1.66^def***A***^	1.53^ef***B***^	0.38^ef***A***^	0.07^cdefg***A***^	0.41^def***A***^
	NaOH	12.26^E***A***^	7.338^IJ***B***^	45.5^HI***B***^	196.9^D***A***^	0.61^E***A***^	5.26^M***B***^	3.24^B***A***^	0.09^K***B***^	2.09^ab***A***^	0.32^EF***B***^	0.08^BCDEFG***A***^	0.09^DE***B***^
13	H_2_O	10.00^efg***A***^	15.48^fg***A***^	111.0^de***A***^	137.8^de***B***^	0.61^d***A***^	16.05^ef***A***^	3.22^de***A***^	1.84^cd***A***^	1.68^de***A***^	0.45^cd***A***^	0.09^cd***A***^	0.44^de***A***^
	NaOH	3.875^J***B***^	7.42^I***B***^	11.9^L***B***^	156.8^J***A***^	0.45^K***B***^	1.65^P***B***^	2.10^I***B***^	0.04^M***B***^	1.54^DEF***B***^	0.26^FGH***B***^	0.07^EFG***B***^	0.08^DE***B***^
14	H_2_O	11.60^cd***A***^	18.94^cd***A***^	137.4^c***A***^	162.9^c***A***^	0.84^c***A***^	19.51^bc***A***^	3.86^c***A***^	2.24^b***A***^	1.96^c***A***^	0.42^de***A***^	0.07^cdefg***A***^	0.55^bc***A***^
	NaOH	3.61^J***B***^	7.00^JK***B***^	29.7^K***B***^	148.1^K***B***^	0.42^L***B***^	1.99^o***B***^	2.03^I***B***^	0.11^K***B***^	1.46^EF***B***^	0.17^H***B***^	0.08^BCDEFG***A***^	0.08^DE***B***^
15	H_2_O	9.23^fgh***A***^	16.77^ef***A***^	114.1^d***A***^	131.1^ef***B***^	0.57^d***A***^	15.11^fg***A***^	3.00^ef***A***^	1.74^cde***A***^	1.58^def***B***^	0.43^de***A***^	0.08^cdef***A***^	0.44^de***A***^
	NaOH	9.21^G***A***^	11.70^C***B***^	95.7^A***B***^	148.6^K***A***^	0.39^M***B***^	12.2^B***B***^	2.74^EF***B***^	1.50^B***B***^	1.70^CDE***A***^	0.43^BCD***A***^	0.09^BCDE***A***^	0.31^AB***B***^
16	H_2_O	11.70^cd***B***^	18.22^cde***A***^	109.4^de***A***^	145.4^d***B***^	0.50^de***B***^	17.15^de***A***^	3.36^d***A***^	1.93^c***A***^	1.74^d***B***^	0.42^de***B***^	0.09^c***B***^	0.49^cd***A***^
	NaOH	13.37^BCD***A***^	10.43^EF***B***^	49.4^FGH***B***^	200.3^BC***A***^	0.67^BC***A***^	11.64^DE***B***^	2.72^EF***B***^	0.97^FG***B***^	2.29^A***A***^	0.50^AB***A***^	0.15^A***A***^	0.17^CDE***B***^
17	H_2_O	11.90^cd***B***^	19.31^c***A***^	114.0^d***A***^	160.7^c***B***^	0.84^c***A***^	19.13^bc***A***^	3.78^c***A***^	2.19^b***A***^	1.96^c***B***^	0.53^b***A***^	0.08^cde***B***^	0.56^bc***A***^
	NaOH	13.20^BCD***A***^	10.34^F***B***^	48.9^FGH***B***^	197.0^D***A***^	0.66^CD***B***^	11.47^F***B***^	2.87^DE***B***^	0.95^G***B***^	2.25^A***A***^	0.49^AB***B***^	0.15^A***A***^	0.17^CDE***B***^
18	H_2_O	16.70^a***A***^	24.17^a***A***^	160.8^b***A***^	198.4^a***A***^	1.03^b***A***^	22.32^a***A***^	4.54^a***A***^	2.62^a***A***^	2.44^a***A***^	0.80^a***A***^	0.23^a***A***^	0.80^a***A***^
	NaOH	12.18^E***B***^	6.26^L***B***^	43.5^IJ***B***^	176.7^EF***B***^	0.58^G***B***^	6.27^L***B***^	2.88^DE***B***^	0.18^K***B***^	1.85^BCD***B***^	0.29^EFG***B***^	0.07^GH***B***^	0.08^DE***B***^
19	H_2_O	6.84^ijk***B***^	12.06^ijk***A***^	91.7^ghi***A***^	107.1^hi***B***^	0.30^fgh***B***^	11.07^klm***A***^	2.32^hi***B***^	1.24^hij***A***^	1.28^hı***iB***^	0.26^h***B***^	0.06^defg***B***^	0.36^efgh***A***^
	NaOH	7.89^H***A***^	8.82^G***B***^	40.7^J***B***^	160.5^I***A***^	0.55^H***A***^	7.54^K***B***^	2.92^DE***A***^	0.63^I***B***^	1.82^BCDE***A***^	0.34^DEF***A***^	0.09^BC***B***^	0.12^DE***B***^
20	H_2_O	6.59^jk***B***^	10.47^k***B***^	78.0^jkl***B***^	95.7^i***B***^	0.28^gh***B***^	9.67^m***B***^	2.07^i***B***^	1.10^j***B***^	1.15^i***B***^	0.31^gh***B***^	0.07^cdefg***A***^	0.31^h***A***^
	NaOH	13.70^AB***A***^	14.77^A***A***^	86.7^B***A***^	174.6^F***A***^	0.52^I***A***^	13.16^A***A***^	3.16^BC***A***^	1.57^A***A***^	2.03^ABC***A***^	0.45^ABC***A***^	0.08^BCDEFG***A***^	0.32^AB***A***^

a H_2_O-treated samples lettered with lowercase whereas NaOH-treated samples lettered with uppercase are significantly different in each column (*p* < 0.05). Italic and bold uppercases define that there is a significant difference between H_2_O-treated and NaOH-treated samples (*p* < 0.05).

A strong positive correlation between the antioxidant capacity, TPC, and L*, a*, and b* values for NaOH-treated samples was observed, whereas only DPPH results of H_2_O-treated samples were negatively correlated with the L*, a*, and b* values (*p* < 0.05). Thus, it can be concluded that color-related phenolic content can be interpreted with the DPPH method for both reagent types.

Cocoa powder with antioxidant properties has significant implications for producers and consumers in terms of product quality and health benefits. As a rich source of polyphenols and antioxidants, cocoa powder is associated with a variety of health benefits. There are numerous benefits associated with these bioactive compounds in cocoa, including preventing allergies, cancers, oxidative injuries, inflammatory conditions, anxiety, hyperglycemia, and insulin resistance.^41^ It has been shown that the processing of cocoa, including alkalization, can affect the levels of polyphenols and flavonoids, which are important antioxidant properties of cocoa products.^42,43^ The alkalization process has been shown to impair the flavanol content and antioxidant activity of cocoa powders, as well as the levels of proanthocyanidins and flavonoids.^39−44^ A number of factors, such as particle size, fat content, and alkaloid composition, can affect cocoa powder’s antioxidant properties, which can affect the overall quality and health benefits of cocoa-based products.^36−46^ Similarly, there are several factors that can affect the bioavailability of phenolic compounds in cocoa products, including soluble fibers, sugar content, and processing methods.^47,48^ To optimize the antioxidant properties of cocoa products, producers should consider factors such as fat content, processing methods, and the presence of other ingredients that could affect their bioavailability and stability.^49,50^ It is possible for consumers to take advantage of cocoa powder’s antioxidant properties by incorporating it into their diet. This could potentially help maintain cardiovascular health, reduce oxidative stress, and provide the overall health benefits associated with antioxidant-rich foods.^51,52^

### Aroma Profile Analysis

The final aroma compounds of the cocoa powder are formed during alkalization. It has been determined that 600 different substances, including alcohols, esters, aldehydes, ketones, carboxylic acids, and pyrazines, are odor-active substances.^53^ The pyrazine molecules make up approximately 40% of the cocoa powder aroma profile.^54^ An indicator ratio was determined for the effect of cocoa roasting on the aromatic composition. Trimethylpyrazine (TrMP) is associated with burnt, woody, nutty, and caramel flavors, and tetramethylpyrazine (TMP) is responsible for the cocoa, coffee, green, mocha, and roast flavor. It was reported that the cocoa roasting indicator is explained as the TrMP/TMP ratio in the range of 0.400–0.667.^26^ The TrMP/TMP values of samples were measured between 0.782 and 1.290 for H_2_O-treated cocoa powder samples and 0.250–1.179 for NaOH-treated cocoa powder samples (Table 5). The temperature was found to have a significant effect on the TrMP/TMP ratio in H_2_O-treated cocoa powder samples, and a positive correlation was found between these two parameters (*r* = 0.459; *p* < 0.05). This can be explained by the fact that the pyrazine compound formation is mainly driven by temperature-related reactions since there is no significant pH change during alkalization. For NaOH-treated cocoa powder samples, the interaction between temperature and the alkali solution ratio was significant (*p* < 0.05). Furthermore, it was reported that the correlations of moisture content and water activity with TrMP/TMP may be linked with the presence of these parameters among the important environmental conditions.^26^ No matter if they are neutral, acidic, or nitrogen- and sulfur-containing compounds, high- and low-volatility flavor compounds can be vulnerable to chemical alterations brought on by a variety of interactions, such as oxidation, hydrolysis, thermal degradation, photooxidation, polymerization of unsaturated compounds, and interactions with protein in food systems.^55^ On the other hand, the alkalization of pyrazine-containing compounds leads to a higher concentration of volatile compounds, which may be further amplified by heating and basic conditions, potentially leading to a Maillard reaction in cocoa powders.^56^

**Table 5:** TrMP/TMP Results of H_2_O- and NaOH-Alkalized Samples

H_2_O			NaOH		
powder TrMP/TMP	distillate TrMP/TMP	powder/total pyrazine^a^ (%)	powder TrMP/TMP	distillate TrMP/TMP	powder/total pyrazine^a^ (%)
1.18	1.66	21.6	1.18	1.12	4.6
1.18	1.84	12.8	0.98	1.05	3.3
1.18	1.70	19.8	0.43	0.97	5.2
1.19	1.96	56.1	1.00	1.11	5.9
0.88	1.77	6.6	0.96	1.14	5.4
0.91	1.75	8.9	0.92	1.28	7.1
0.95	2.04	8.9	1.06	1.13	4.8
0.89	2.05	7.0	1.00	1.14	4.9
1.00	1.97	10.5	0.33	1.15	5.7
1.00	1.73	6.2	0.67	1.88	3.6
1.29	1.80	11.5	1.00	1.05	3.5
0.85	1.76	10.4	0.68	1.20	5.1
0.78	1.95	8.6	0.25	0.99	9.6
1.04	2.11	11.7	0.25	1.30	6.3
1.00	2.17	9.7	1.15	1.13	7.1
0.94	2.01	10.0	1.12	1.12	4.9
0.94	2.07	7.8	1.05	1.15	5.0
0.85	1.91	5.6	0.75	0.94	7.2
0.88	2.05	12.1	0.75	1.40	4.0
0.95	1.92	10.2	0.43	1.62	6.0

a Total pyrazine: alkalized cocoa powder pyrazine + distillate pyrazine.

TrMP/TMP of natural cocoa powder was measured as 1.14. When the TrMP/TMP ratios were compared, it was observed that H_2_O-treated samples had results higher than those of NaOH-treated samples. It is expected that H_2_O-treated samples had a more burnt, woody, nutty, caramel flavor, whereas the NaOH-treated samples had more cocoa, coffee, green, mocha, and roast flavor.

Alkali solution and temperature interaction boosted the reactions that ended with TMP, which showed a decrease in TrMP/TMP. Higher pH conditions in the matrix provided a suitable environment for TMP formation, whereas TrMP formation was supported by the temperature. In general, when the distillate TrMP/TMP ratios were compared, H_2_O-treated samples resulted in higher ratios compared to the NaOH-treated samples. The main output of the aroma profile analysis was the ratio of total pyrazine of alkalized cocoa powder/total pyrazine (alkalized cocoa powder + distillate). The aroma released from the food matrix depends on several factors such as chemical interactions (hydrogen, hydrophobic, ionic, or covalent bonding) or food composition may lead to adsorption, entrapment, and diffusion limitation of the aroma compounds.^57^ Phenolic compounds are important compounds that contribute to the sensory properties of food such as flavor, color, and taste.^58^ Since these compounds have numerous hydroxyl functional groups and aromatic rings, they interact with flavoring substances and change their volatility and release from food.^59^ Weak interactions and hydrophobic forces, such as π–π stacks stabilized by hydrogen bonds between aroma substances and the galloyl ring of phenolic compounds, affect aroma release.^60,61^ The results showed that when H_2_O was applied for alkalization, the proportion of total pyrazine in alkalized cocoa powder was retained at a higher rate than in NaOH-alkalized samples (Table 5). This could be related to phenolic content, which was found higher in H_2_O-alkalized samples than in NaOH-alkalized samples. On the other hand, these results also showed that a significant proportion of total pyrazine was lost during vacuum processing for both alkalizations.

### Optimization of the Alkalization Process

The cocoa powder alkalization process was further optimized through the RSM approach in terms of L*, a*, and b* color parameters. One of the main purposes of this study was to reveal the potential of H_2_O as an alkalizing agent and an alternative to NaOH for improving the color of natural cocoa powder. For this purpose, the optimum conditions were selected for L*, a*, and b* values for both alkalization conditions to discover if similar color characteristics can be achievable.

The optimum process conditions achieved desirability values of 0.696 for H_2_O alkalization and 0.989 for NaOH alkalization. The optimized parameters of the H_2_O alkalization process were identified as 80 °C, a 30.4% alkalizing solution ratio, and 33.6 min of process time. Predicted values for H_2_O-treated cocoa powder samples are L*: 38.9, a*: 11.58, and b*: 19.13. In the case of the NaOH alkalization process, the optimized parameters were identified as 40 °C, a 41.04% alkali solution ratio, and 31.2 min of process time. The predicted values for NaOH-treated cocoa powder samples are L*: 38.5, a*: 12.07, and b*: 20.00.

**Figure 5:**
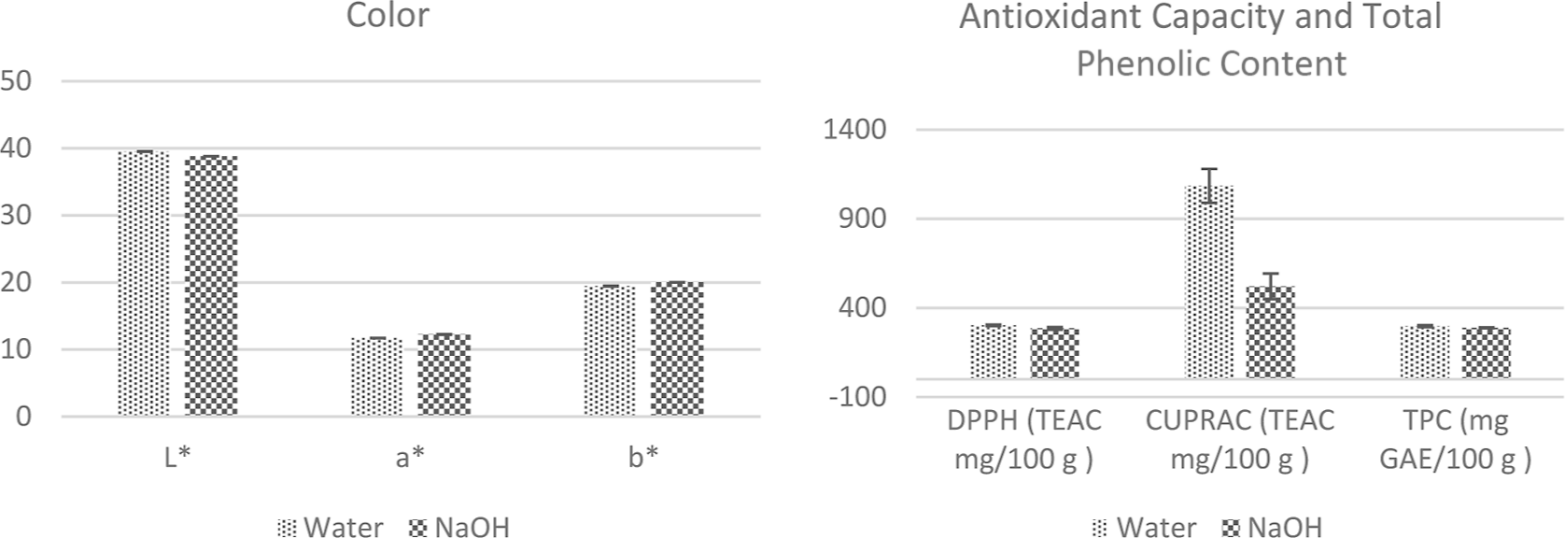
Color, antioxidant, and TPC results of H_2_O-treated and NaOH-treated cocoa powders at optimized process parameters.

The experimental color values obtained were in agreement with those predicted by the model (Figure 5). The Δ*E* value calculated using the predicted color values was 0.94, whereas the Δ*E* value of the alkalized cocoa powders obtained using the optimized parameters was found as 0.92 which means that there is no color difference according to Δ*E* < 3 industrial standards. This result shows that the color results obtained with the NaOH-treated cocoa powder samples can also be obtained with H_2_O. Antioxidant and TPC analyses were performed with optimized-condition cocoa powders. H_2_O-treated cocoa powders’ antioxidant capacity and TPC analysis results were found to be significantly higher than those of NaOH-treated cocoa powders, in line with the previous experimental study (Figure 5).

The extractable pH of natural cocoa powders ranged from 5.2 to 5.6, and alkalized cocoa powder was classified into three groups: lightly treated (pH 6.50–7.20), medium-treated (pH 7.21–7.60), and heavily treated (pH 7.61 or higher).^39^ The color of lightly treated cocoa powder is relatively lighter than those of medium- and heavy-treated cocoa powder. Although the pH values of cocoa powder alkalized with H_2_O do not fall into this classification, it is predicted that the process with H_2_O alone will be sufficient for producing lightly treated alkalized cocoa powder in terms of color parameters.

## Conclusions

Research on cocoa powder alkalization has primarily focused on the use of alkali agents and their effects on the quality parameters of the cocoa powder. Our study aimed to present an alternative alkalization method based on green chemistry principles and investigate the interactions and correlations between the alkalization process parameters and the aroma profile, antioxidant capacity, TPC, phenolic profile, color, and pH of cocoa powders.

We found that alkalization with water positively affected the antioxidant capacity and phenolic content of the cocoa powders. The optimization study revealed that color improvement can also be achieved with H_2_O-treated samples, obtaining similar color values to those treated with sodium hydroxide. Although NaOH significantly improved the color, as reported in the literature, it also led to a significant decrease in the antioxidant capacity and phenolic content of the cocoa powders.

Significant differences were observed in the aroma profiles of cocoa powder samples alkalized with H_2_O and NaOH. The trimethylpyrazine/tetramethylpyrazine (TrMP/TMP) ratios indicated that cocoa powders with different tastes and aromas can be produced by altering processing parameters that affect the aroma component composition. Notably, the retention of the TrMP/TMP ratio was higher in H_2_O-treated cocoa powders than in those treated with NaOH. This finding suggests that the odor generated during the removal of excess moisture can be reduced when treated with H_2_O, which is an important environmental benefit for cocoa-processing facilities. However, further studies are required to investigate the sensory profile of the final products and the individual aroma compounds of alkalized cocoa powders to better understand the aroma profile.

In summary, the findings of this study indicate that H_2_O can be used as an alternative to NaOH for alkalization in the chocolate industry, particularly for lightly treated alkalized cocoa powders with high antioxidant activity. The production of H_2_O-treated cocoa powder can be easily implemented in existing production methods without significant changes in the process. However, further research is necessary to gain a deeper understanding of the aroma recovery, yield, and quality. Overall, H_2_O can replace NaOH used in lightly or medium-alkalized cocoa powders, potentially lowering emissions, reducing costs, and creating a more sustainable and manageable process for the industry.

## Supporting Information Available

RSM contour plots, ANOVA coefficient tables, homoscedasticity test results, and correlation study results (PDF)
