# Supplementary material for: Comparison of Physicochemical Properties, Antioxidants, and Aroma Profiles of Water- and Sodium-Hydroxide-Treated Natural Cocoa Powder
Source: ACS Omega. 2024 Aug 5;9(33):35730–43. doi: 10.1021/acsomega.4c04173 (PMC11339982; doi:10.1021/acsomega.4c04173)
Supplement: Supplementary file 1 — ao4c04173_si_001.pdf [file ao4c04173_si_001.pdf]

SUPPORTING INFORMATION

Title:

Comparison of Physicochemical Properties, Antioxidants and Aroma Profiles of Water and Sodium-Hydroxide Treated Natural Cocoa Powder

Authors: Ertan Sahin[a], Fatma Duygu Ceylan[a], Aslı Barla Demirkoz[bc], Aslı Can Karaca[a], Esra Capanoglu[a*]

RSM Contour Plots

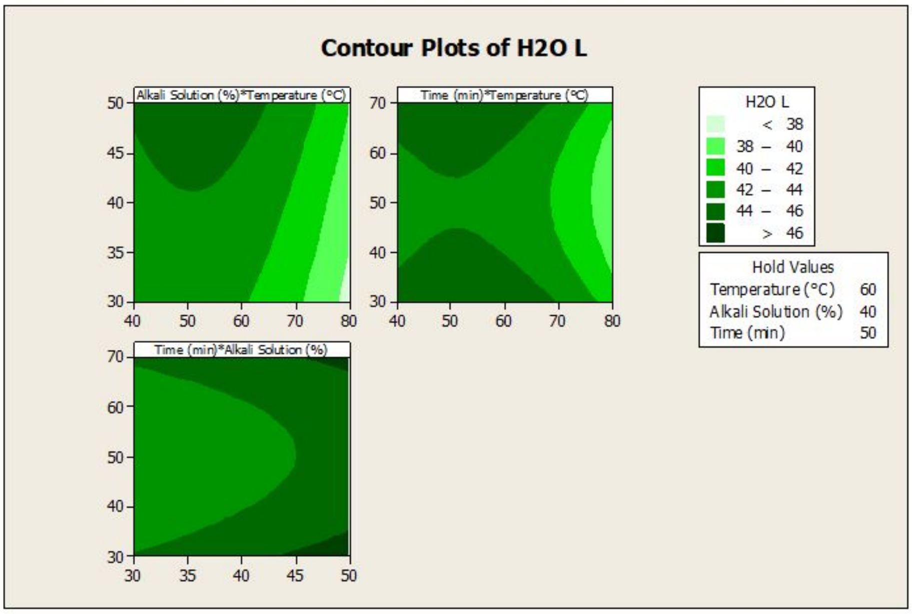

Figure 1S. H_2O-treated cocoa powder L* values contour plots according to process parameters

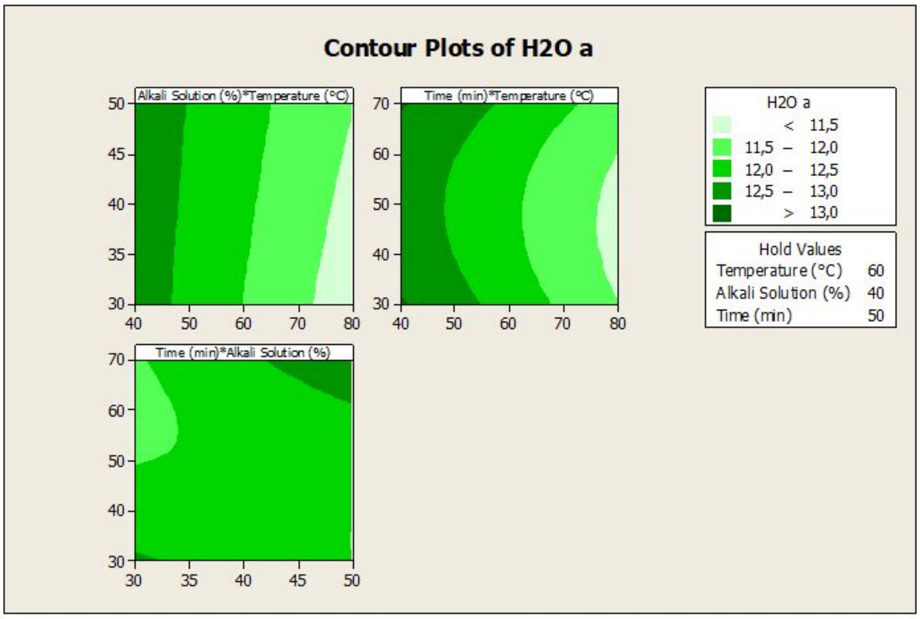

Figure 2S. H_2O-treated cocoa powder a* values contour plots according to process parameters

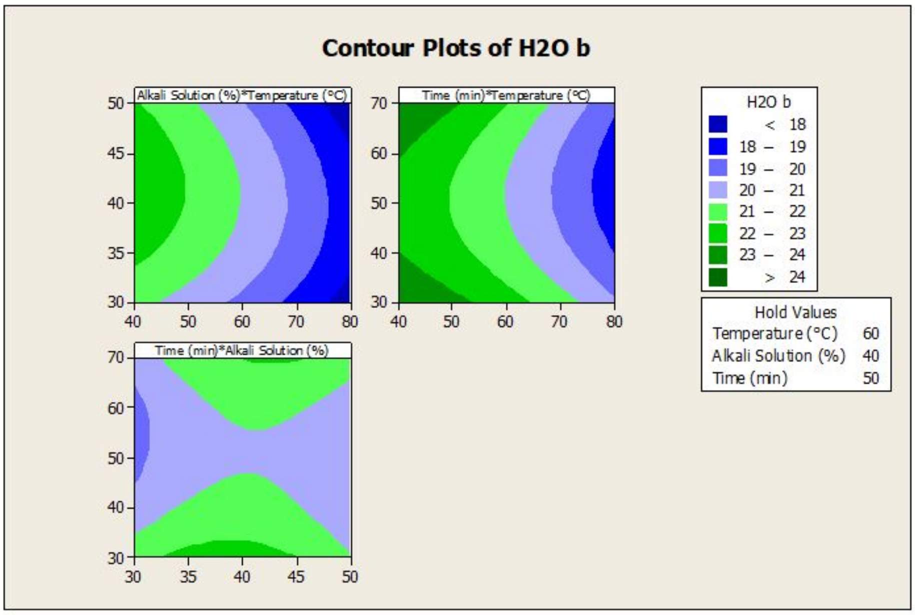

Figure 3S. H_2O-treated cocoa powder b* values contour plots according to process parameters

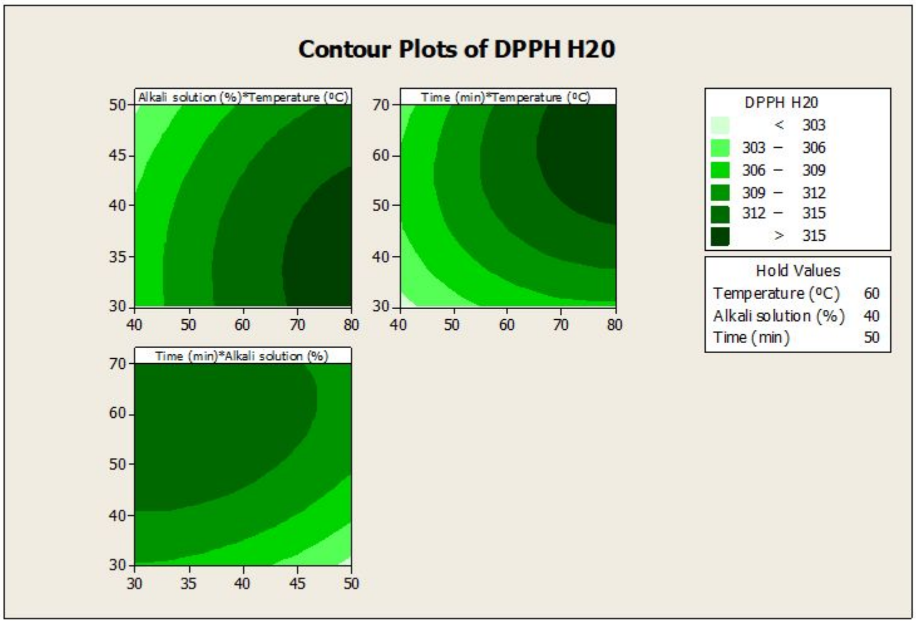

Figure 4S. H_2O-treated cocoa powder DPPH results contour plots according to process parameters

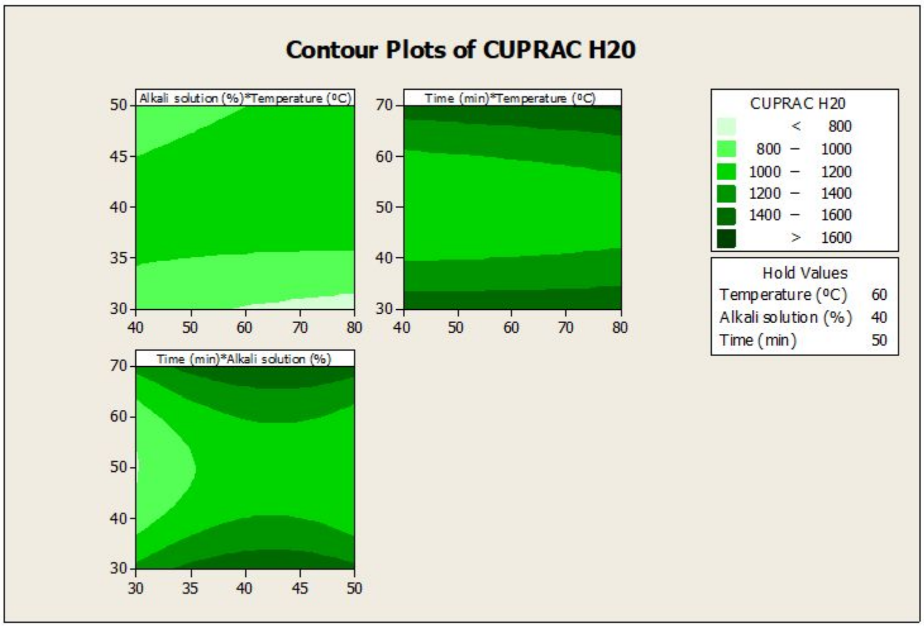

Figure 5S. H_2O-treated cocoa powder CUPRAC results contour plots according to process parameters

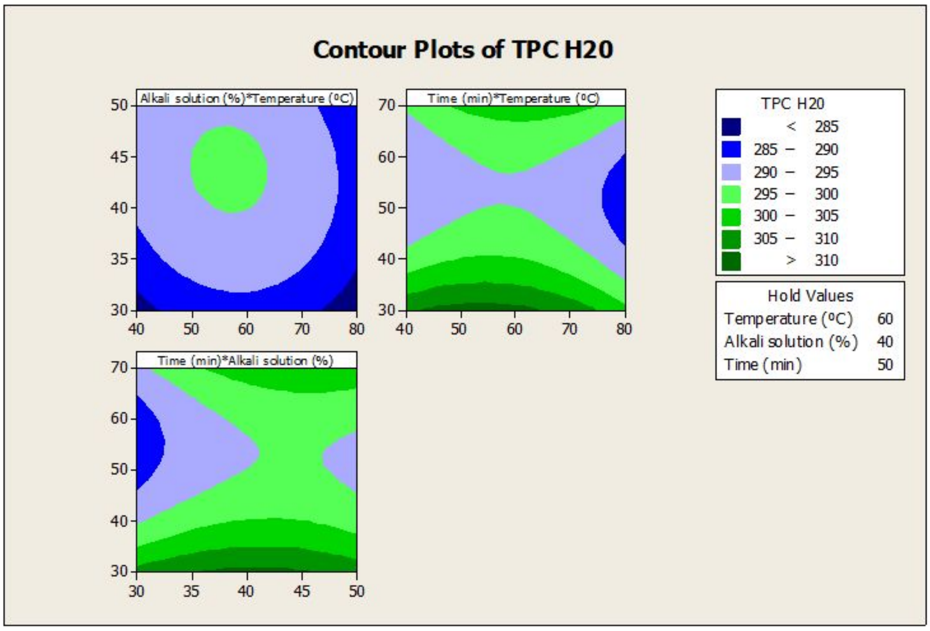

Figure 6S. H_2O-treated cocoa powder TPC results contour plots according to process parameters

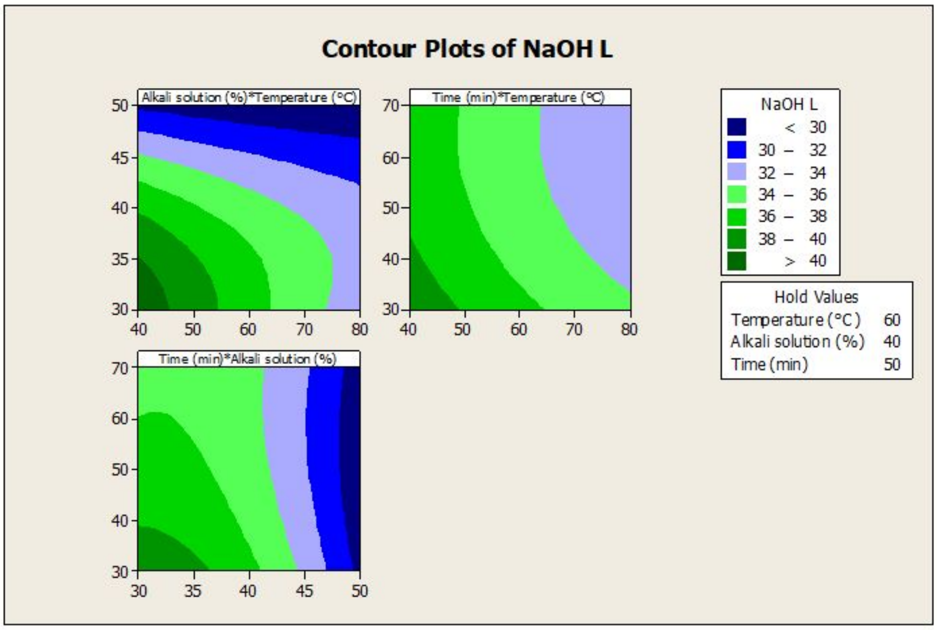

Figure 7S. NaOH-treated cocoa powder L* values contour plots according to process parameters

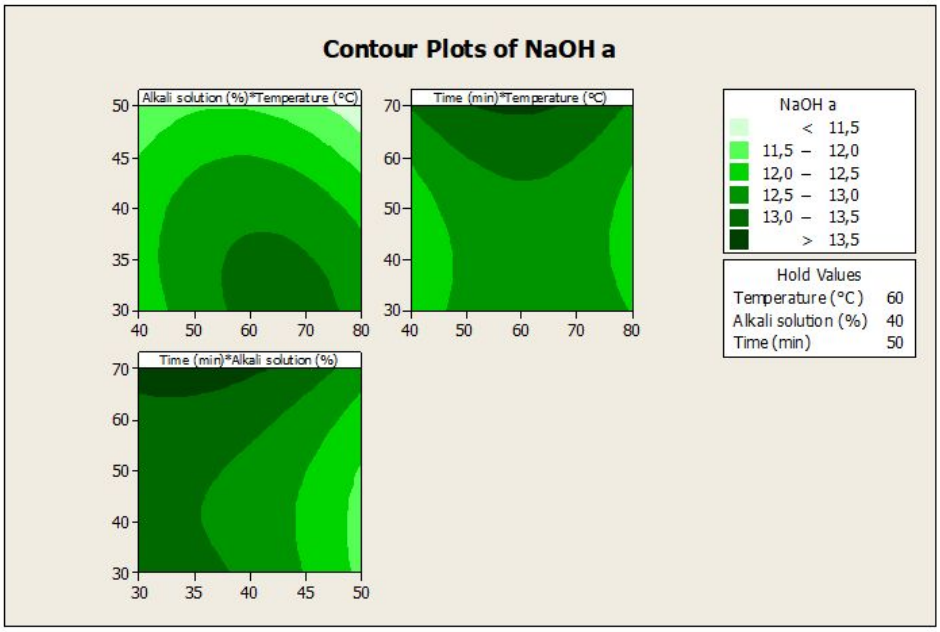

Figure 8S. NaOH-treated cocoa powder a* values contour plots according to process parameters

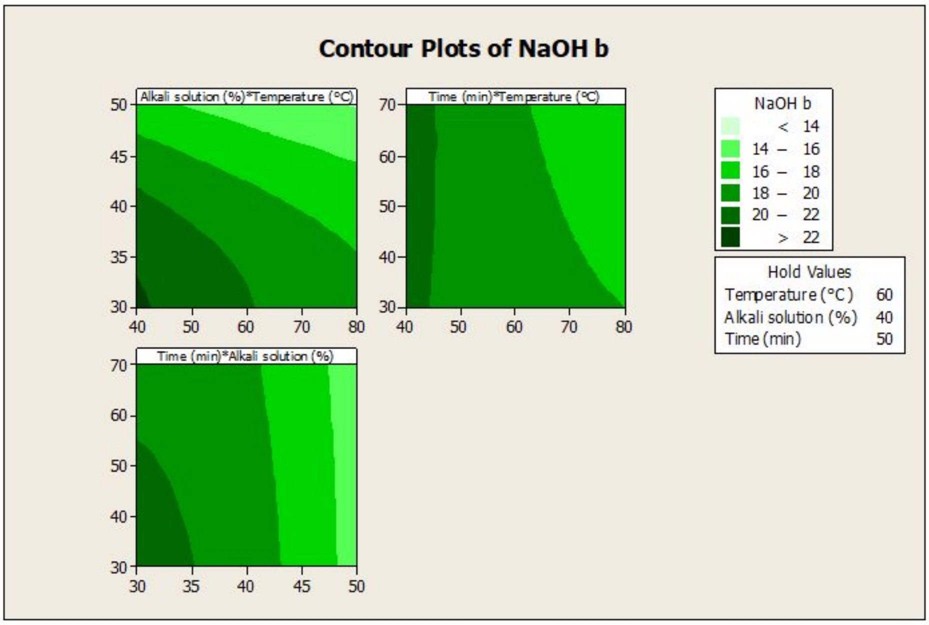

Figure 9S. NaOH-treated cocoa powder b* values contour plots according to process parameters

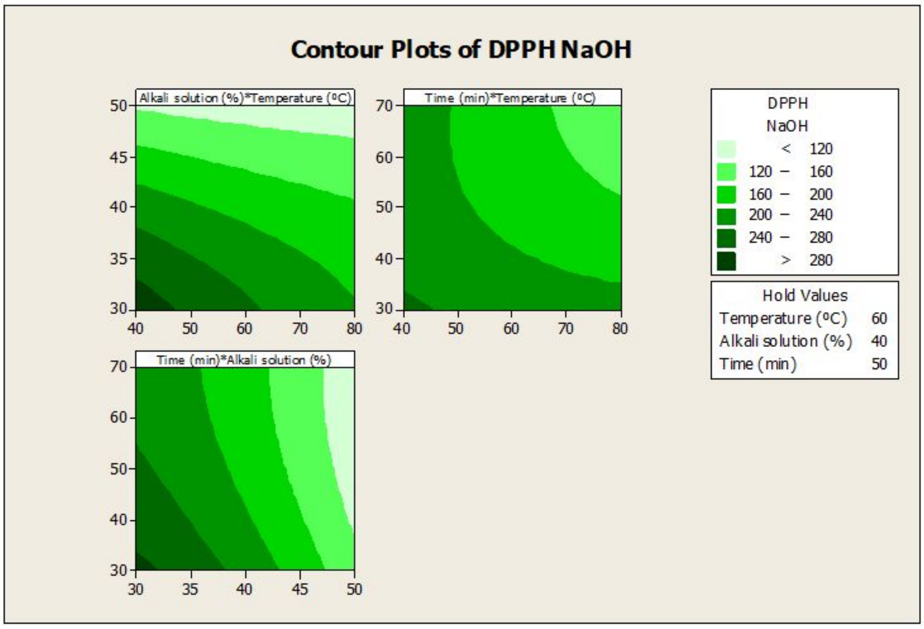

Figure 10S. NaOH-treated cocoa powder DPPH results contour plots according to process parameters

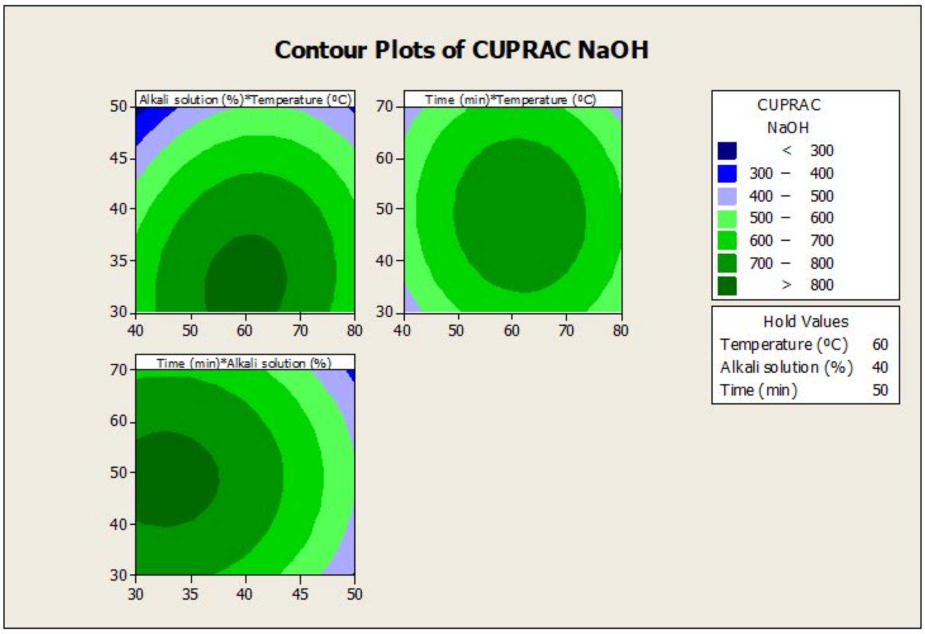

Figure 11S. NaOH-treated cocoa powder CUPRAC results contour plots according to process parameters

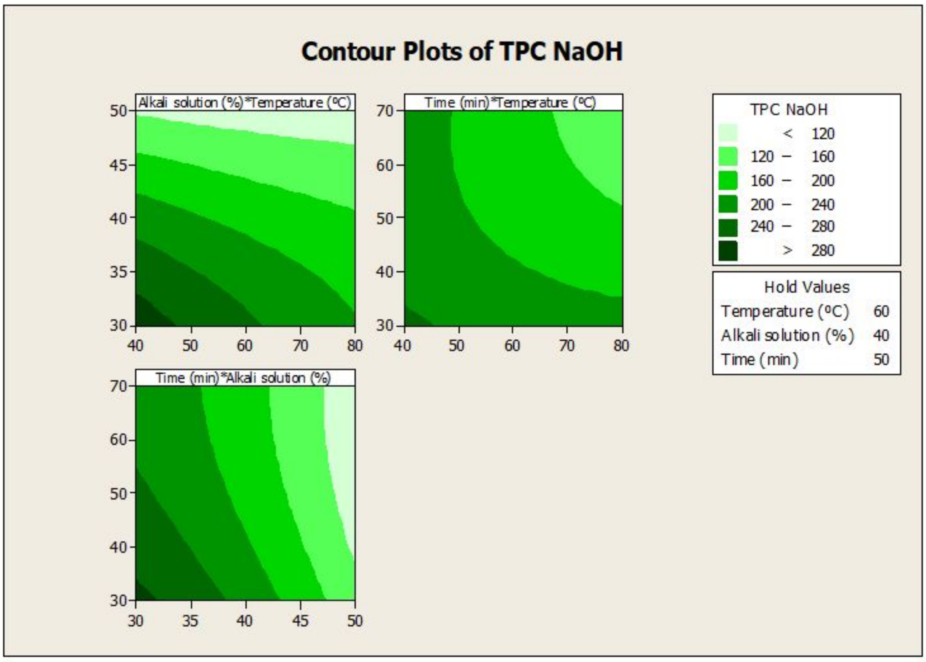

Figure 12S. NaOH-treated cocoa powder TPC results contour plots according to process parameters

ESTIMATED REGRESSION COEFFICIENTS

Table 1S. Estimated regression coefficients H_2O-treated cocoa powder L* results using coded units.

Term	Coef	SE Coef	T	P
Constant	43,3391	0,1643	263,705	0,000
Temperature (°C)	-2,2490	0,1512	-14,877	0,000
Alkali Solution (%)	1,2470	0,1512	8,249	0,000
Time (min)	-0,0760	0,1512	-0,503	0,626
Temperature (°C) *Temperature (°C)	-2,4177	0,2883	-8,387	0,000
Alkali Solution (%) *Alkali Solution (%)	0,0523	0,2883	0,181	0,860
Time (min)*Time (min)	2,0773	0,2883	7,206	0,000
Temperature (°C) *Alkali Solution (%)	0,1313	0,1690	0,777	0,455
Temperature (°C) *Time (min)	-0,1987	0,1690	-1,176	0,267
Alkali Solution (%) *Time (min)	-0,1787	0,1690	-1,058	0,315

S = 0,478065   PRESS = 31,2566
R-Sq = 97,45% R-Sq(pred) = 65,18% R-Sq(adj) = 95,16%

Table 2S. Estimated regression coefficients H_2O-treated cocoa powder L* results using data in uncoded units.

Term	Coef
Constant	35,6447
Temperature (°C)	0,611462
Alkali Solution (%)	0,0881943
Time (min)	-0,457556
Temperature (°C) *Temperature (°C)	-0,00604432
Alkali Solution (%) *Alkali Solution (%)	0,000522727
Time (min)*Time (min)	0,00519318
Temperature (°C) *Alkali Solution (%)	0,000656250
Temperature (°C) *Time (min)	-4,96875E-04
Alkali Solution (%) *Time (min)	-8,93750E-04

Table 3S. Estimated regression coefficients H_2O-treated cocoa powder a* results using coded units.

Term	Coef	SE Coef	T	P
Constant	12,0764	0,07323	164,906	0,000
Temperature (°C)	-0,7000	0,06736	-10,391	0,000
Alkali Solution (%)	0,0880	0,06736	1,306	0,221
Time (min)	0,0600	0,06736	0,891	0,394
Temperature (°C) *Temperature (°C)	-0,0109	0,12846	-0,085	0,934
Alkali Solution (%) *Alkali Solution (%)	-0,0009	0,12846	-0,007	0,994
Time (min)*Time (min)	0,2691	0,12846	2,095	0,063
Temperature (°C) *Alkali Solution (%)	0,0650	0,07531	0,863	0,408
Temperature (°C) *Time (min)	0,0625	0,07531	0,830	0,426
Alkali Solution (%) *Time (min)	0,3650	0,07531	4,846	0,001

S = 0,213022   PRESS = 3,43127
R-Sq = 93,46% R-Sq(pred) = 50,57% R-Sq(adj) = 87,58%

Table 4S. Estimated regression coefficients H_2O-treated cocoa powder a* results using data in uncoded units.

Term	Coef
Constant	20,1422
Temperature (°C)	-0,0525398
Alkali Solution (%)	-0,101223
Time (min)	-0,146648
Temperature (°C) *Temperature (°C)	-2,72727E-05
Alkali Solution (%) *Alkali Solution (%)	-9,09091E-06
Time (min)*Time (min)	0,000672727
Temperature (°C) *Alkali Solution (%)	0,000325000
Temperature (°C) *Time (min)	0,000156250
Alkali Solution (%) *Time (min)	0,00182500

Table 5S. Estimated regression coefficients H_2O-treated cocoa powder b* results using coded units.

Term	Coef	SE Coef	T	P
Constant	20,9384	0,1929	108,552	0,000
Temperature (°C)	-2,1370	0,1774	-12,044	0,000
Alkali Solution (%)	0,1450	0,1774	0,817	0,433
Time (min)	-0,1990	0,1774	-1,122	0,288
Temperature (°C) *Temperature (°C)	-0,3759	0,3383	-1,111	0,293
Alkali Solution (%) * Alkali Solution (%)	-1,0559	0,3383	-3,121	0,011
Time (min)*Time (min)	1,2741	0,3383	3,766	0,004
Temperature (°C) *Alkali Solution (%)	-0,2800	0,1984	-1,411	0,188
Temperature (°C) *Time (min)	-0,2500	0,1984	-1,260	0,236
Alkali Solution (%) *Time (min)	0,4200	0,1984	2,117	0,060

S = 0,561088   PRESS = 24,1224
R-Sq = 94,55% R-Sq(pred) = 58,26% R-Sq(adj) = 89,65%

Table 6S Estimated regression coefficients H_2O-treated cocoa powder b* results using data in uncoded units.

Term	Coef
Constant	13,9172
Temperature (°C)	0,0931727
Alkali Solution (%)	0,838227
Time (min)	-0,374973
Temperature (°C) *Temperature (°C)	-9,39773E-04
Alkali Solution (%) *Alkali Solution (%)	-0,0105591
Time (min)*Time (min)	0,00318523
Temperature (°C) *Alkali Solution (%)	-0,00140000
Temperature (°C) *Time (min)	-6,25000E-04
Alkali Solution (%) *Time (min)	0,00210000

Table 7S. Estimated regression coefficients NaOH-treated cocoa powder L* results using coded units.

Term	Coef	SE Coef	T	P
Constant	34,8058	0,3967	87,742	0,000
Temperature (°C)	-2,5270	0,3649	-6,925	0,000
Alkali solution (%)	-4,1360	0,3649	-11,335	0,000
Time (min)	-1,0270	0,3649	-2,815	0,018
Temperature (°C) *Temperature (°C)	0,3405	0,6958	0,489	0,635
Alkali solution (%) *Alkali solution (%)	-2,1845	0,6958	-3,139	0,011
Time (min)*Time (min)	0,6905	0,6958	0,992	0,344
Temperature (°C) *Alkali solution (%)	1,7350	0,4080	4,253	0,002
Temperature (°C) *Time (min)	-0,0425	0,4080	-0,104	0,919
Alkali solution (%) *Time (min)	0,8000	0,4080	1,961	0,078

S = 1,15390 PRESS = 95,1427
R-Sq = 95,60% R-Sq(pred) = 68,57% R-Sq(adj) = 91,64%

Table 8S. Estimated regression coefficients NaOH-treated cocoa powder L* results using data in uncoded units.

Term	Coef
Constant	62,4263
Temperature (°C)	-0,570174
Alkali solution (%)	0,613536
Time (min)	-0,377589
Temperature (°C) *Temperature (°C)	0,000851136
Alkali solution (%) * Alkali solution (%)	-0,0218455
Time (min)*Time (min)	0,00172614
Temperature (°C) *Alkali solution (%)	0,00867500
Temperature (°C) *Time (min)	-1,06250E-04
Alkali solution (%) *Time (min)	0,00400000

Table 9S. Estimated regression coefficients NaOH-treated cocoa powder a* results using coded units.

Term	Coef	SE Coef	T	P
Constant	12,8792	0,07317	176,019	0,000
Temperature (°C)	0,0570	0,06731	0,847	0,417
Alkali solution (%)	-0,5710	0,06731	-8,484	0,000
Time (min)	0,3400	0,06731	5,052	0,000
Temperature (°C) *Temperature (°C)	-0,6055	0,12835	-4,717	0,001
Alkali solution (%) * Alkali solution (%)	-0,3555	0,12835	-2,769	0,020
Time (min)*Time (min)	0,3595	0,12835	2,801	0,019
Temperature (°C) *Alkali solution (%)	-0,3037	0,07525	-4,037	0,002
Temperature (°C) *Time (min)	-0,1138	0,07525	-1,512	0,162
Alkali solution (%) *Time (min)	0,0887	0,07525	1,179	0,266

S = 0,212840 PRESS = 4,38418
R-Sq = 94,43% R-Sq(pred) = 46,11% R-Sq(adj) = 89,42%

Table 10S. Estimated regression coefficients NaOH-treated cocoa powder a* results using data in uncoded units.

Term	Coef
Constant	1,64235
Temperature (°C)	0,259455
Alkali solution (%)	0,296201
Time (min)	-0,0735739
Temperature (°C) *Temperature (°C)	-0,00151364
Alkali solution (%) * Alkali solution (%)	-0,00355455
Time (min)*Time (min)	0,000898864
Temperature (°C) *Alkali solution (%)	-0,00151875
Temperature (°C) *Time (min)	-2,84375E-04
Alkali solution (%) *Time (min)	0,000443750

Table 11S. Estimated regression coefficients NaOH-treated cocoa powder b* results using coded units.

Term	Coef	SE Coef	T	P
Constant	18,6542	0,2512	74,266	0,000
Temperature (°C)	-1,6750	0,2311	-7,249	0,000
Alkali solution (%)	-2,5300	0,2311	-10,950	0,000
Time (min)	-0,3270	0,2311	-1,415	0,187
Temperature (°C) *Temperature (°C)	0,2095	0,4406	0,476	0,645
Alkali solution (%) *Alkali solution (%)	-1,0455	0,4406	-2,373	0,039
Time (min)*Time (min)	-0,0405	0,4406	-0,092	0,929
Temperature (°C) *Alkali solution (%)	0,2612	0,2583	1,011	0,336
Temperature (°C) *Time (min)	-0,5187	0,2583	-2,008	0,072
Alkali solution (%) *Time (min)	0,2038	0,2583	0,789	0,449

S = 0,730652 PRESS = 68,9197
R-Sq = 94,97% R-Sq(pred) = 35,03% R-Sq(adj) = 90,44%

Table 12S. Estimated regression coefficients NaOH-treated cocoa powder b* results using data in uncoded units.

Term	Coef
Constant	20,8044
Temperature (°C)	-0,134020
Alkali solution (%)	0,454051
Time (min)	0,0308261
Temperature (°C) *Temperature (°C)	0,000523864
Alkali solution (%) * Alkali solution (%)	-0,0104545
Time (min)*Time (min)	-1,01136E-04
Temperature (°C) *Alkali solution (%)	0,00130625
Temperature (°C) *Time (min)	-0,00129687
Alkali solution (%) *Time (min)	0,00101875

Table 13S. Estimated regression coefficients H_2O-treated cocoa powder TPC results using coded units.

Term	Coef	SE Coef	T	P	
Constant	295,037	3,153	93,578	0,000	
Temperature (^{0}C)		-1,568	2,900	-0,541	0,601
Alkali solution (%)	2,856	2,900	0,985	0,348	
Time (min)	-4,228	2,900	-1,458	0,176	
Temperature (^{0}C) *Temperature (^{0}C)	-5,924	5,530	-1,071	0,309	
Alkali solution (%) *Alkali solution (%)	-3,977	5,530	-0,719	0,489	
Time (min)*Time (min)	11,780	5,530	2,130	0,059	
Temperature (^{0}C) *Alkali solution (%)	-0,927	3,243	-0,286	0,781	

Temperature (^{0}C) *Time (min)	2,311	3,243	0,713	0,492
Alkali solution (%) *Time (min)	2,210	3,243	0,682	0,511

S = 9,17127 PRESS = 5059,76
R-Sq = 47,58% R-Sq(pred) = 0,00% R-Sq(adj) = 0,41%

Table 14S. Estimated regression coefficients H_2O-treated cocoa powder TPC results using data in uncoded units.

Term	Coef
Constant	283,886
Temperature (^{0}C)	1,59511
Alkali solution (%)	3,19254
Time (min)	-3,94510
Temperature (^{0}C) *Temperature (^{0}C)	-0,0148088
Alkali solution (%) *Alkali solution (%)	-0,0397698
Time (min)*Time (min)	0,0294500
Temperature (^{0}C) *Alkali solution (%)	-0,00463265
Temperature (^{0}C) *Time (min)	0,00577732
Alkali solution (%) *Time (min)	0,0110512

Table 15S. Estimated regression coefficients NaOH-treated cocoa powder TPC results using coded units.

Term	Coef	SE Coef	T	P
Constant	188,616	10,202	18,488	0,000
Temperature (^{0}C)	-29,044	9,384	-3,095	0,011
Alkali solution (%)	-72,944	9,384	-7,773	0,000
Time (min)	-25,674	9,384	-2,736	0,021
Temperature (^{0}C) *Temperature (^{0}C)	4,485	17,896	0,251	0,807
Alkali solution (%) *Alkali solution (%)	-14,815	17,896	-0,828	0,427
Time (min)*Time (min)	11,735	17,896	0,656	0,527
Temperature (^{0}C) *Alkali solution (%)	19,307	10,492	1,840	0,096
Temperature (^{0}C) *Time (min)	-13,443	10,492	-1,281	0,229
Alkali solution (%) *Time (min)	5,483	10,492	0,523	0,613

S = 29,6764 PRESS = 61547,2
R-Sq = 89,32% R-Sq(pred) = 25,37% R-Sq(adj) = 79,71%

Table 16S. Estimated regression coefficients NaOH-treated cocoa powder TPC results using data in uncoded units.

Term	Coef
Constant	694,060
Temperature (^{0}C)	-4,97875
Alkali solution (%)	-2,60491
Time (min)	-3,29746
Temperature (^{0}C) *Temperature (^{0}C)	0,0112114
Alkali solution (%) *Alkali solution (%)	-0,148155
Time (min)*Time (min)	0,0293364
Temperature (^{0}C) *Alkali solution (%)	0,0965375
Temperature (^{0}C) *Time (min)	-0,0336063
Alkali solution (%) *Time (min)	0,0274125

Table 17S. Estimated regression coefficients H_2O-treated cocoa powder CUPRAC results using coded units.

Term	Coef	SE Coef	T	P
Constant	1088,11	61,79	17,610	0,000
Temperature (C)	39,26	56,84	0,691	0,505
Alkali solution (%)	106,14	56,84	1,867	0,091
Time (min)	8,98	56,84	0,158	0,878
Temperature (C)*Temperature (C)	7,18	108,39	0,066	0,949
Alkali solution (%) *Alkali solution (%)	-196,31	108,39	-1,811	0,100
Time (min)*Time (min)	467,14	108,39	4,310	0,002
Temperature (C) *Alkali solution (%)	120,84	63,55	1,902	0,086
Temperature (C) *Time (min)	22,70	63,55	0,357	0,728
Alkali solution (%) *Time (min)	14,20	63,55	0,223	0,828

S = 179,738 PRESS = 1988723
R-Sq = 75,53% R-Sq(pred) = 0,00% R-Sq(adj) = 53,50%

Table 18S. Estimated regression coefficients H_2O-treated cocoa powder CUPRAC results using data in uncoded units.

Term	Coef
Constant	2128,90
Temperature (C)	-27,1956
Alkali solution (%)	127,861
Time (min)	-122,581
Temperature (C)*Temperature (C)	0,0179458
Alkali solution (%) *Alkali solution (%)	-1,96310
Time (min)*Time (min)	1,16786
Temperature (C) *Alkali solution (%)	0,604191
Temperature (C) *Time (min)	0,0567474
Alkali solution (%) *Time (min)	0,0709973

Table 19S. Estimated regression coefficients NaOH-treated cocoa powder CUPRAC results using coded units.

Term	Coef	SE Coef	T	P
Constant	777,377	51,49	15,097	0,000
Temperature (C)	30,854	47,37	0,651	0,529
Alkali solution (%)	-158,502	47,37	-3,346	0,007
Time (min)	-17,992	47,37	-0,380	0,712
Temperature (C)*Temperature (C)	-178,348	90,32	-1,975	0,077
Alkali solution (%) * Alkali solution (%)	-115,389	90,32	-1,278	0,230
Time (min)*Time (min)	-132,647	90,32	-1,469	0,173
Temperature (C)*Alkali solution (%)	33,099	52,96	0,625	0,546
Temperature (C)*Time (min)	-9,200	52,96	-0,174	0,866
Alkali solution (%) *Time (min)	7,654	52,96	0,145	0,888

S = 149,783 PRESS = 1692236
R-Sq = 80,81% R-Sq(pred) = 0,00% R-Sq(adj) = 63,54%

Table 20S. Estimated regression coefficients NaOH-treated cocoa powder CUPRAC results using data in uncoded units.

Term	Coef
Constant	-2511,87
Temperature (C)	49,5772
Alkali solution (%)	64,6181
Time (min)	32,1113
Temperature (C)*Temperature (C)	-0,445870
Alkali solution (%) *Alkali solution (%)	-1,15389
Time (min)*Time (min)	-0,331617
Temperature (C) *Alkali solution (%)	0,165496
Temperature (C) *Time (min)	-0,0229990
Alkali solution (%) *Time (min)	0,0382694

Table 21S. Estimated regression coefficients H_2O-treated cocoa powder DPPH results using coded units.

Term	Coef	SE Coef	T	P
Constant	312,813	1,594	196,265	0,000
Temperature (C)	4,887	1,466	3,333	0,008
Alkali Solution (%)	-2,002	1,466	-1,365	0,202
Time (min)	2,926	1,466	1,996	0,074
Temperature (C)*Temperature (C)	-1,750	2,796	-0,626	0,545
Alkali Solution (%) *Alkali Solution (%)	-1,493	2,796	-0,534	0,605
Time (min)*Time (min)	-2,931	2,796	-1,048	0,319
Temperature (C) *Alkali Solution (%)	0,141	1,639	0,086	0,933
Temperature (C) *Time (min)	1,578	1,639	0,963	0,358
Alkali Solution (%) *Time (min)	1,347	1,639	0,822	0,430

S = 4,63625   PRESS = 1051,63
R-Sq = 71,57% R-Sq(pred) = 0,00% R-Sq(adj) = 45,99%

Table 22S. Estimated regression coefficients H_2O-treated cocoa powder DPPH results using data in uncoded units.

Term	Coef
Constant	267,895
Temperature (C)	0,543776
Alkali Solution (%)	0,615214
Time (min)	0,372675
Temperature (C)*Temperature (C)	-0,00437480
Alkali Solution (%) *Alkali Solution (%)	-0,0149327
Time (min)*Time (min)	-0,00732634
Temperature (C) *Alkali Solution (%)	0,000705801
Temperature (C) *Time (min)	0,00394607
Alkali Solution (%) *Time (min)	0,00673720

Table 23S. Estimated regression coefficients NaOH-treated cocoa powder DPPH results using data in coded units.

Term	Coef	SE Coef	T	P
Constant	283,929	2,324	122,165	0,000
Temperature (C)	-0,914	2,138	-0,427	0,678
Alkali Solution (%)	-15,662	2,138	-7,326	0,000
Time (min)	4,425	2,138	2,070	0,065
Temperature (C) *Temperature (C)	6,905	4,077	1,694	0,121
Alkali Solution (%) *Alkali Solution (%)	-5,690	4,077	-1,396	0,193
Time (min) *Time (min)	1,875	4,077	0,460	0,655
Temperature (C) *Alkali Solution (%)	-4,761	2,390	-1,992	0,074
Temperature (C) *Time (min)	0,064	2,390	0,027	0,979
Alkali Solution (%) *Time (min)	0,141	2,390	0,059	0,954

S = 6,76065 PRESS = 4920,55
R-Sq = 86,91% R-Sq(pred) = 0,00% R-Sq(adj) = 75,13%

Table 24S. Estimated regression coefficients NaOH-treated cocoa powder DPPH results using data in uncoded units.

Term	Coef
Constant	265,846
Temperature (C)	-1,17312
Alkali Solution (%)	4,37875
Time (min)	-0,285349
Temperature (C) *Temperature (C)	0,0172634
Alkali Solution (%) *Alkali Solution (%)	-0,0568999
Time (min)*Time (min)	0,00468729
Temperature (C) *Alkali Solution (%)	-0,0238048
Temperature (C) *Time (min)	0,000160409
Alkali Solution (%) *Time (min)	0,000705801

Table 25S. Estimated regression coefficients H_2O-treated cocoa powder TrMP/TMP results using coded units.

Term	Coef	SE Coef	T	P
Constant	0,95935	0,03732	25,703	0,000
Temperature (C)	0,08870	0,03433	2,583	0,027
Alkali Solution (%)	-0,06351	0,03433	-1,850	0,094
Time (min)	-0,03178	0,03433	-0,926	0,376
Temperature (C)*Temperature (C)	-0,01634	0,06547	-0,250	0,808
Alkali Solution (%) *Alkali Solution (%)	-0,05708	0,06547	-0,872	0,404
Time (min)*Time (min)	0,14047	0,06547	2,145	0,058
Temperature (C)*Alkali Solution (%)	-0,04501	0,03839	-1,172	0,268
Temperature (C)*Time (min)	0,07132	0,03839	1,858	0,093
Alkali Solution (%) *Time (min)	0,03456	0,03839	0,900	0,389

S = 0,108574 PRESS = 1,25816
R-Sq = 68,43% R-Sq(pred) = 0,00% R-Sq(adj) = 40,02%

Table 26S. Estimated regression coefficients H_2O-treated cocoa powder TrMP/TMP results using data in uncoded units.

Term	Coef
Constant	1,18481
Temperature (C)	0,00942265
Alkali Solution (%)	0,0441717
Time (min)	-0,0543151
Temperature (C) *Temperature (C)	-4,08407E-05
Alkali Solution (%) *Alkali Solution (%)	-5,70751E-04
Time (min)*Time (min)	0,000351172
Temperature (C) *Alkali Solution (%)	-2,25035E-04
Temperature (C) *Time (min)	0,000178292
Alkali Solution (%) *Time (min)	0,000172786

Table 27S. Estimated regression coefficients NaOH-treated cocoa powder TrMP/TMP results using coded units.

Term	Coef	SE Coef	T	P
Constant	0,92913	0,09036	10,283	0,000
Temperature (C)	0,05108	0,08312	0,615	0,553
Alkali Solution (%)	-0,10244	0,08312	-1,232	0,246
Time (min)	-0,12272	0,08312	-1,476	0,171
Temperature (C) *Temperature (C)	-0,01775	0,15850	-0,112	0,913
Alkali Solution (%) * Alkali Solution (%)	-0,17753	0,15850	-1,120	0,289
Time (min)*Time (min)	-0,06523	0,15850	-0,412	0,689
Temperature (C) *Alkali Solution (%)	0,23442	0,09293	2,523	0,030
Temperature (C) *Time (min)	-0,01298	0,09293	-0,140	0,892
Alkali Solution (%) *Time (min)	0,12850	0,09293	1,383	0,197

S = 0,262835 PRESS = 4,51796
R-Sq = 62,07% R-Sq(pred) = 0,00% R-Sq(adj) = 27,93%

Table 28S. Estimated regression coefficients NaOH-treated cocoa powder TrMP/TMP results using data in uncoded units.

Term	Coef
Constant	2,08526
Temperature (C)	-0,0373806
Alkali Solution (%)	0,0293257
Time (min)	-0,0135826
Temperature (C) *Temperature (C)	-4,43835E-05
Alkali Solution (%) * Alkali Solution (%)	-0,00177525
Time (min)*Time (min)	-1,63063E-04
Temperature (C) *Alkali Solution (%)	0,00117209
Temperature (C) *Time (min)	-3,24557E-05
Alkali Solution (%) *Time (min)	0,000642506

HOMOSCEDASTICITY TEST RESULTS

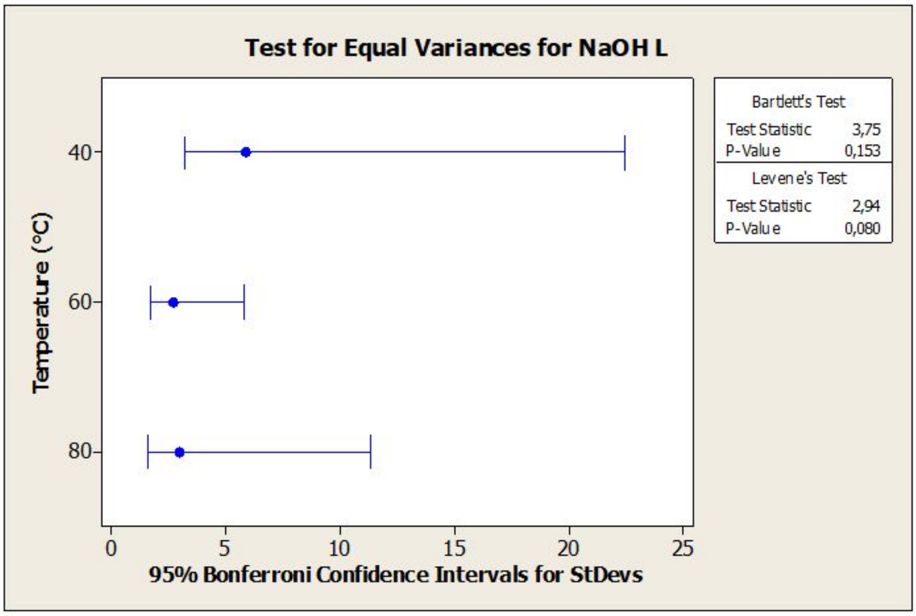

Figure 13S. Test for equal variances: NaOH L* versus temperature (°C)

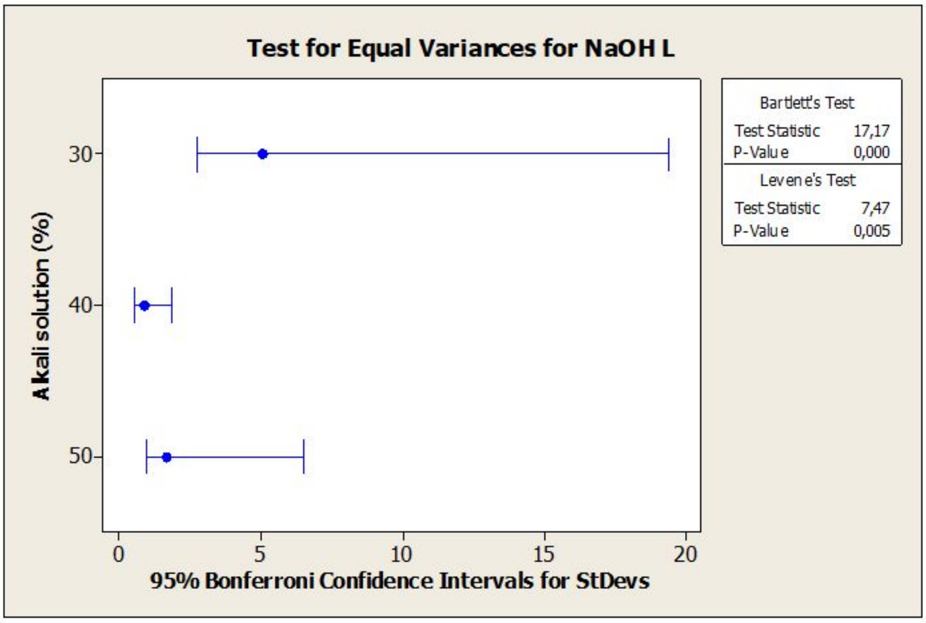

Figure 14S. Test for equal variances: NaOH L* versus alkali solution (%)

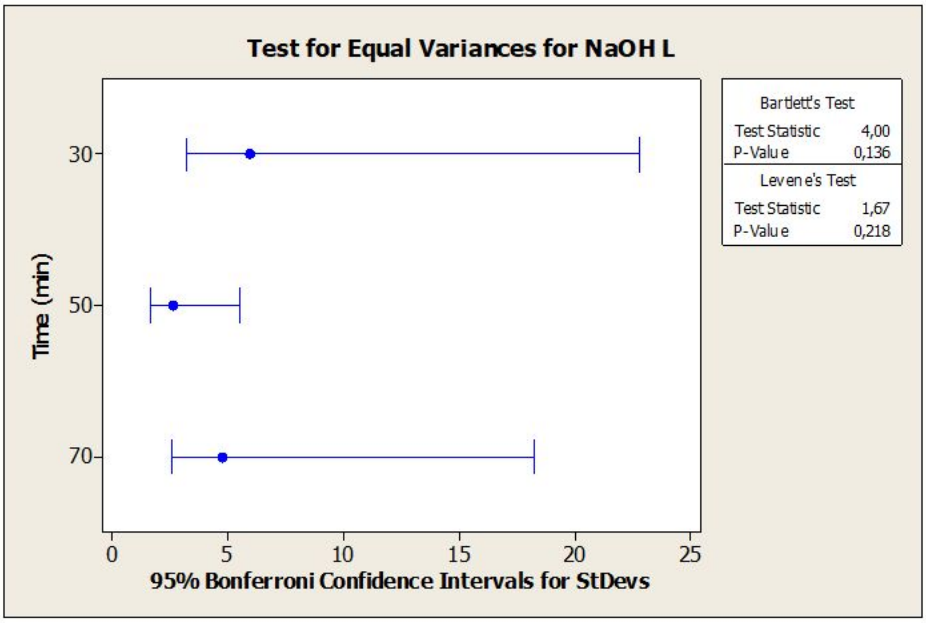

Figure 15S. Test for equal variances: NaOH L* versus time (min)

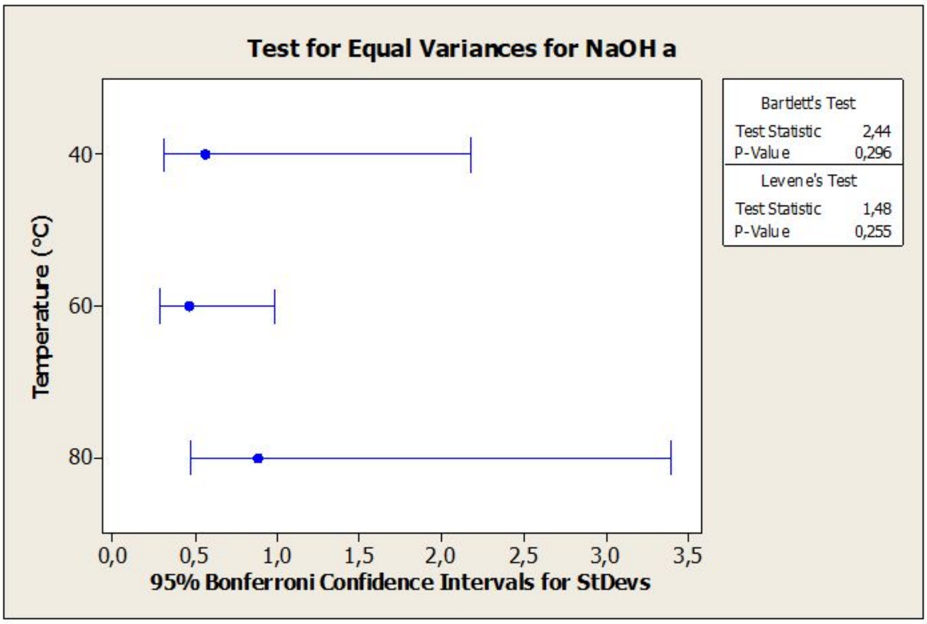

Figure 16S. Test for equal variances: NaOH a* versus temperature (°C)

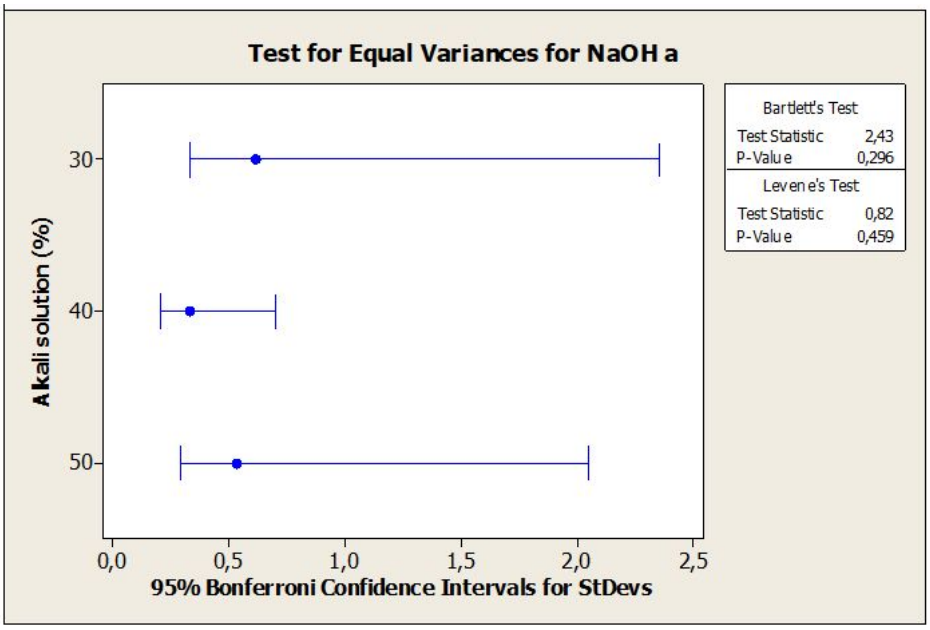

Figure 17S. Test for equal variances: NaOH a* versus alkali solution (%)

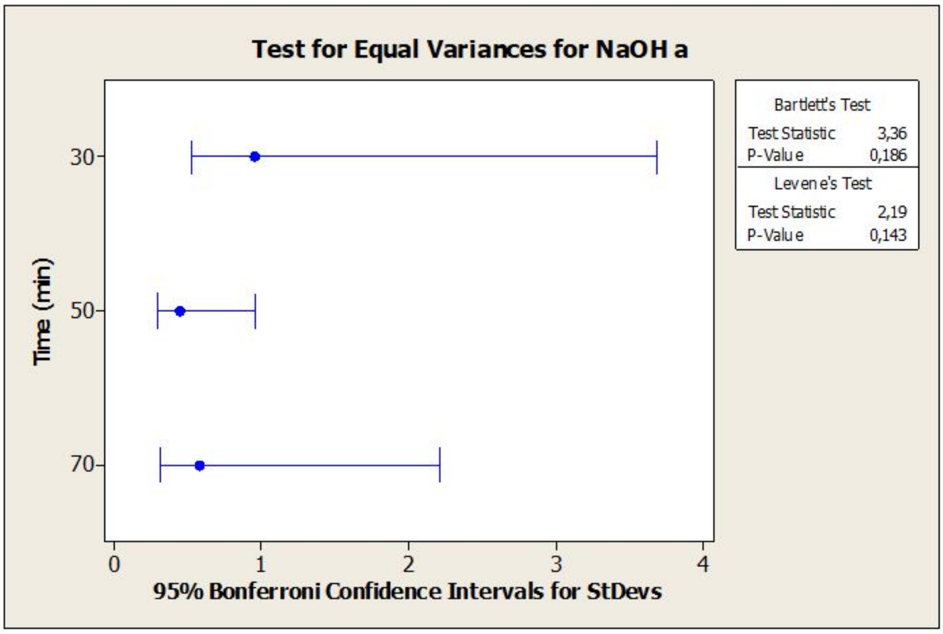

Figure 18S. Test for equal variances: NaOH a* versus time (min)

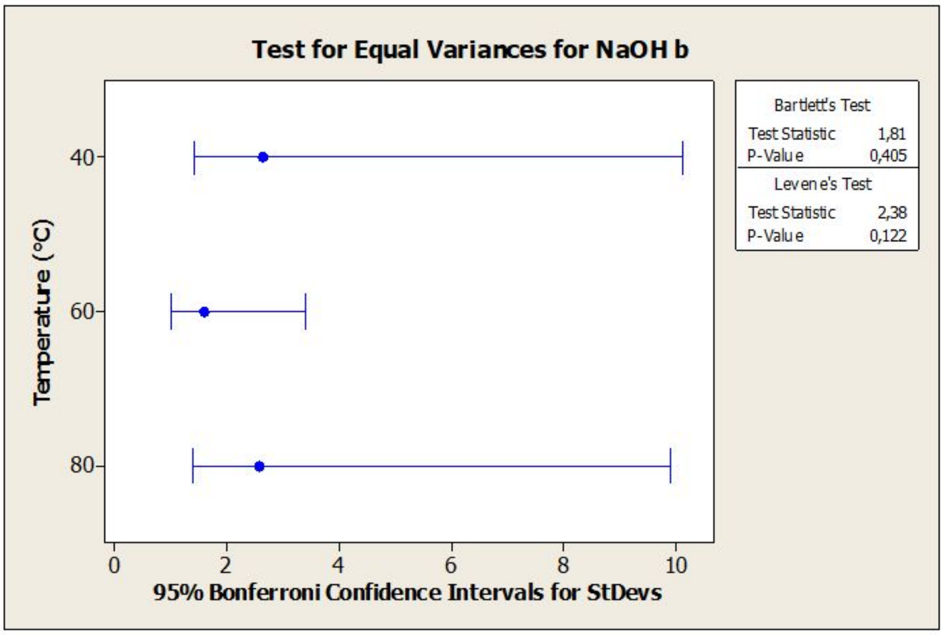

Figure 19S. Test for equal variances: NaOH b* versus temperature (°C)

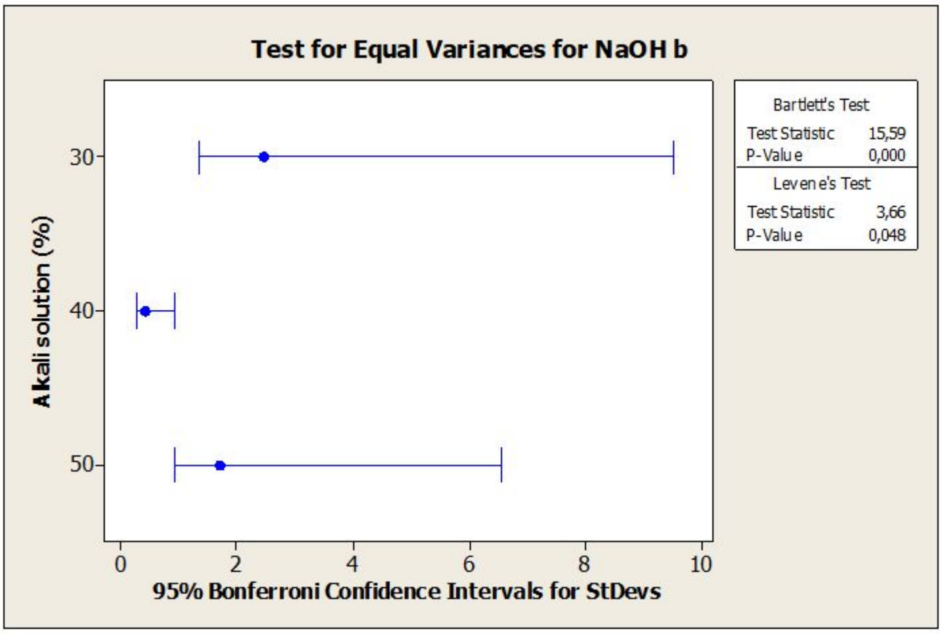

Figure 20S. Test for equal variances: NaOH b* versus alkali solution (%)

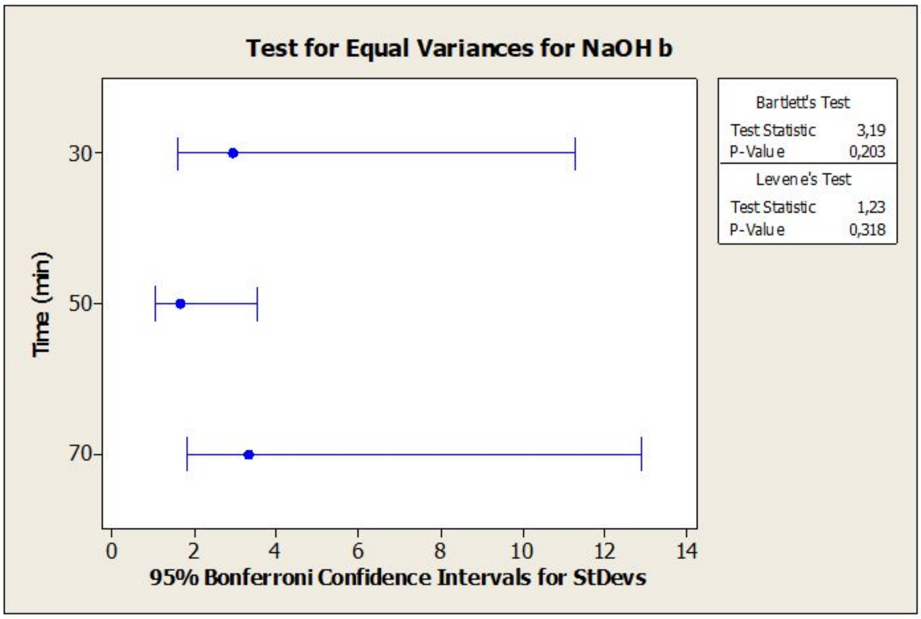

Figure 21S. Test for equal variances: NaOH b* versus time (min)

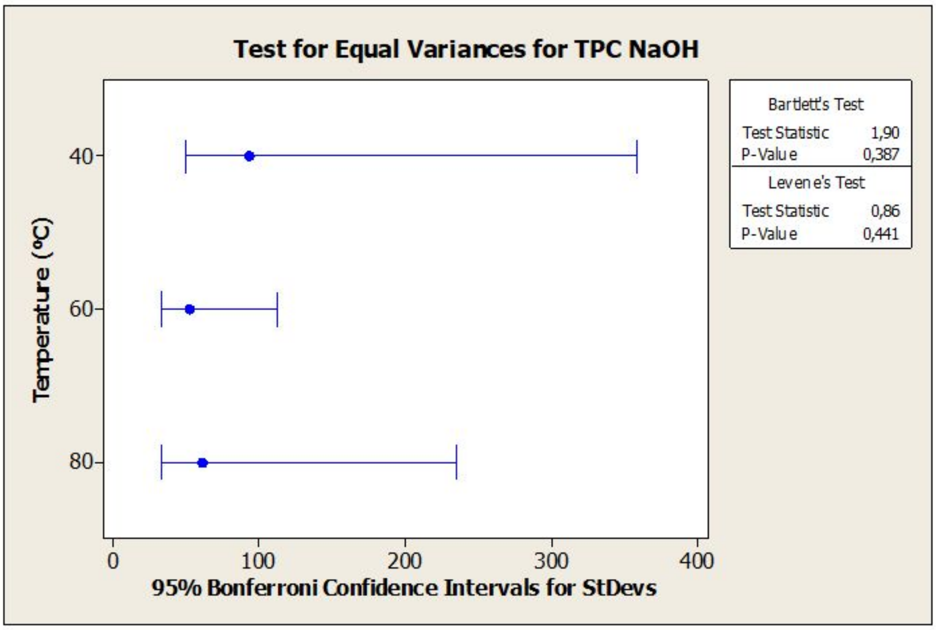

Figure 22S. Test for equal variances: TPC NaOH versus temperature (^{0}C)

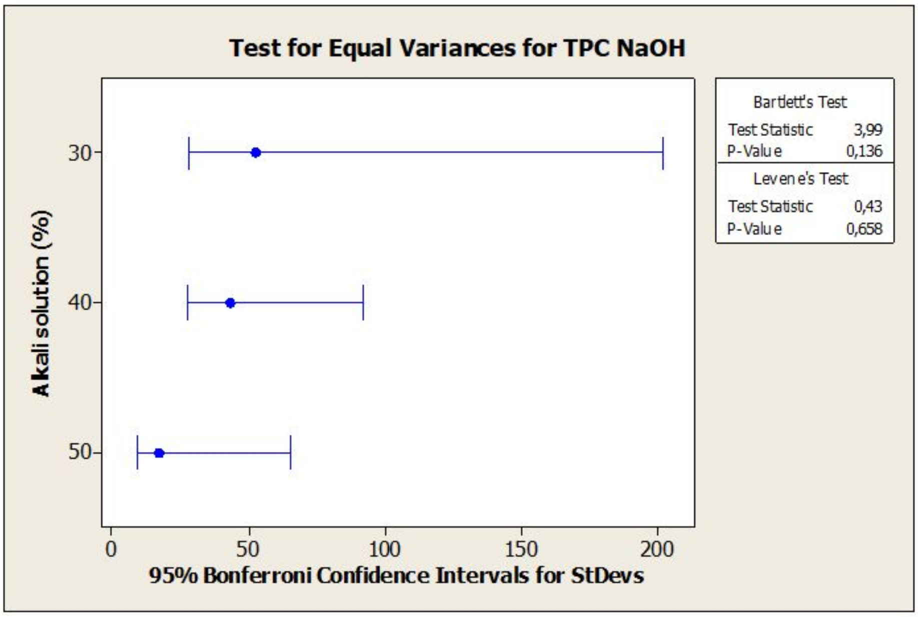

Figure 23S. Test for equal variances: TPC NaOH versus alkali solution (%)

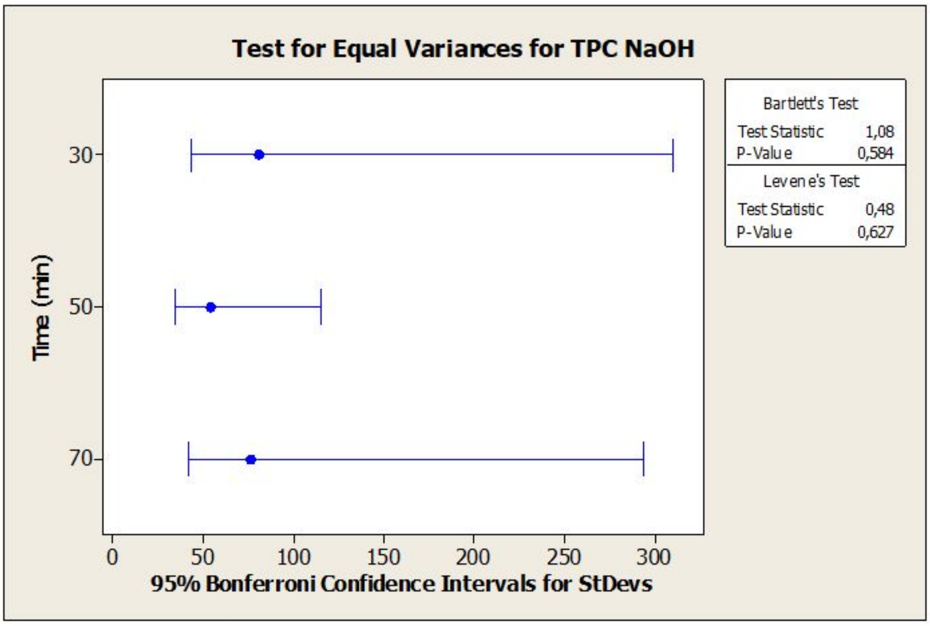

Figure 24S. Test for equal variances: TPC NaOH versus time (min)

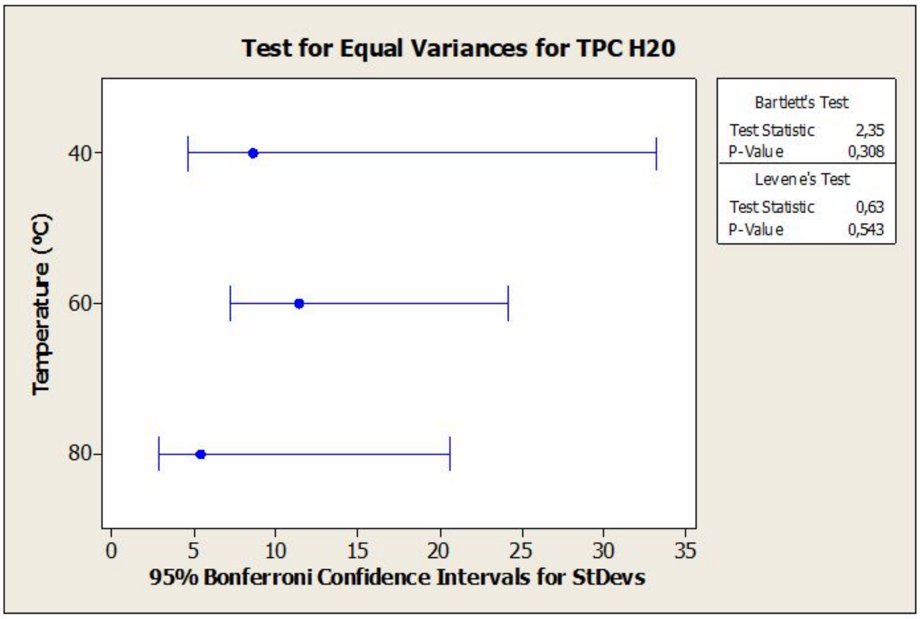

Figure 25S. Test for equal variances: TPC H_2O versus temperature (^{0}C)

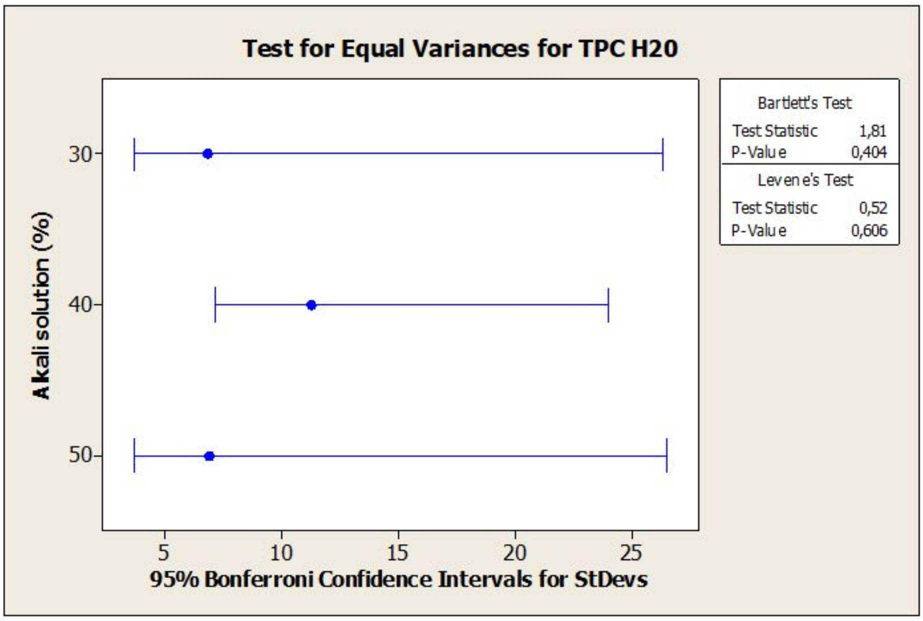

Figure 26S. Test for equal variances: TPC H_2O versus alkali solution (%)

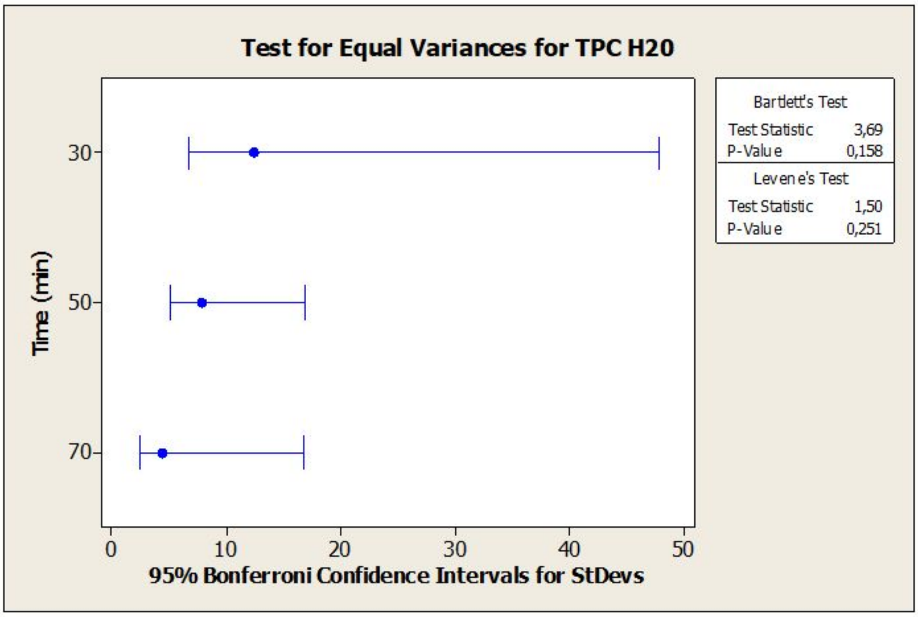

Figure 27S. Test for equal variances: TPC H_2O versus time (min)

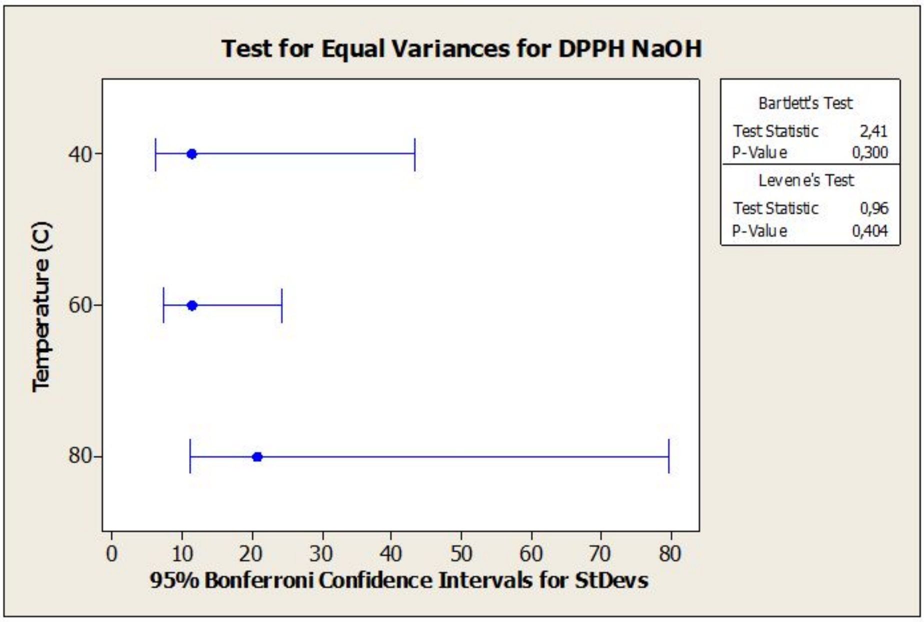

Figure 28S. Test for equal variances: DPPH NaOH versus temperature (C)

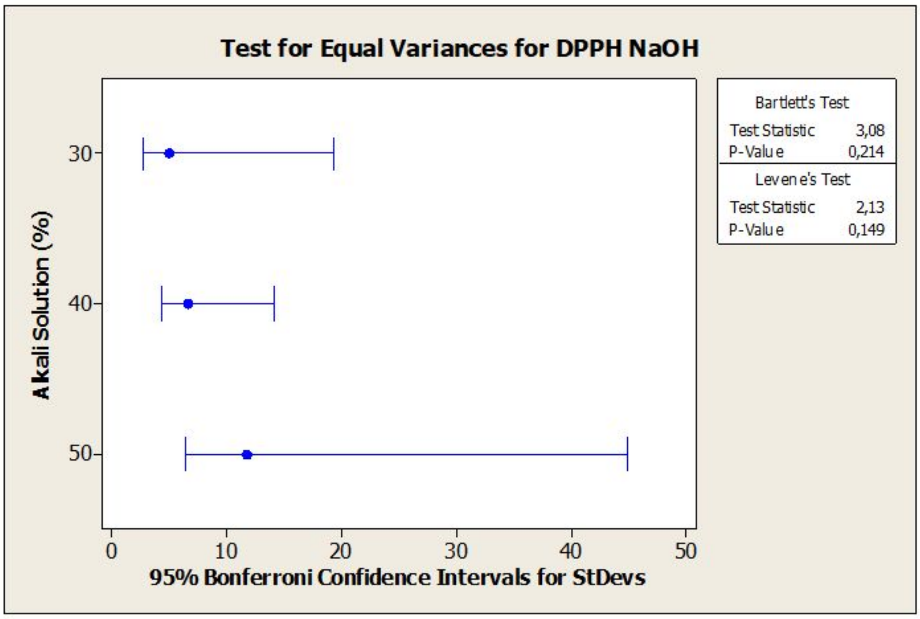

Figure 29S. Test for equal variances: DPPH NaOH versus alkali solution (%)

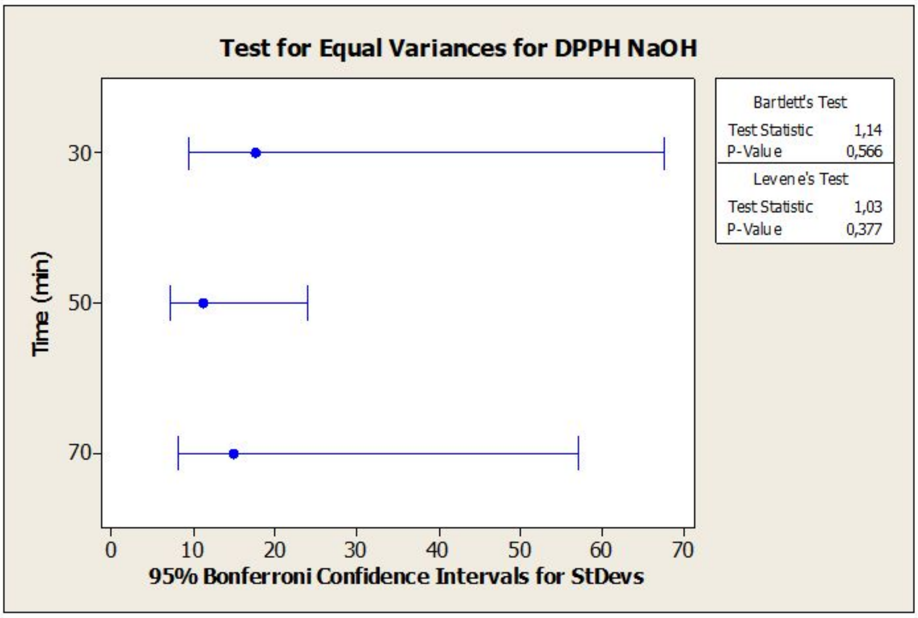

Figure 30S. Test for equal variances: DPPH NaOH versus time (min)

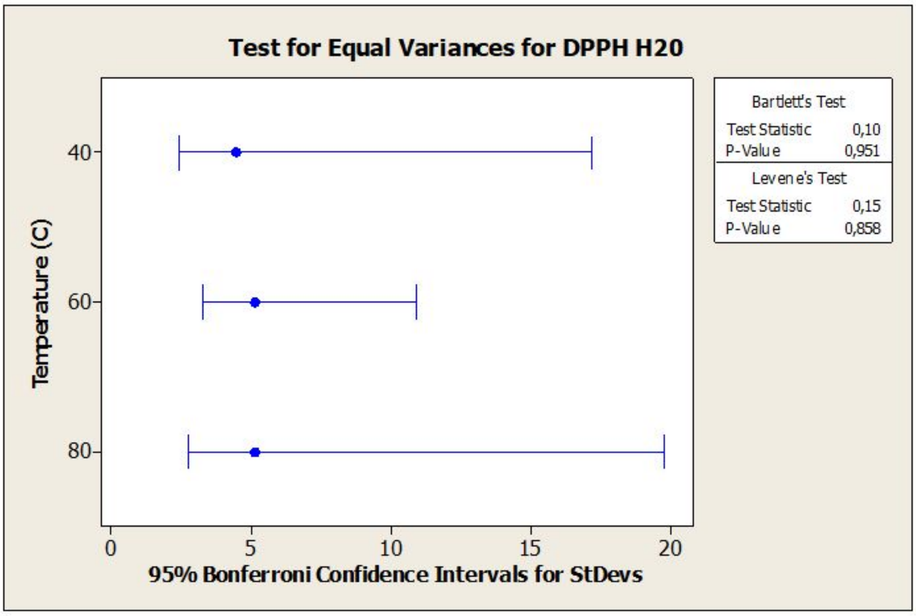

Figure 31S. Test for equal variances: DPPH H_2O versus temperature (C)

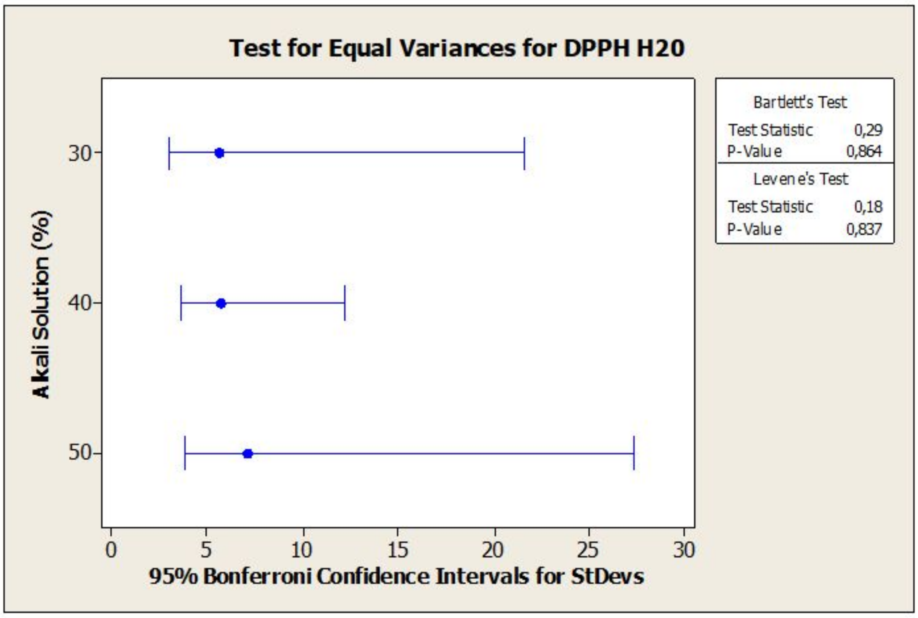

Figure 32. Test for equal variances: DPPH H_2O versus alkali solution (%)

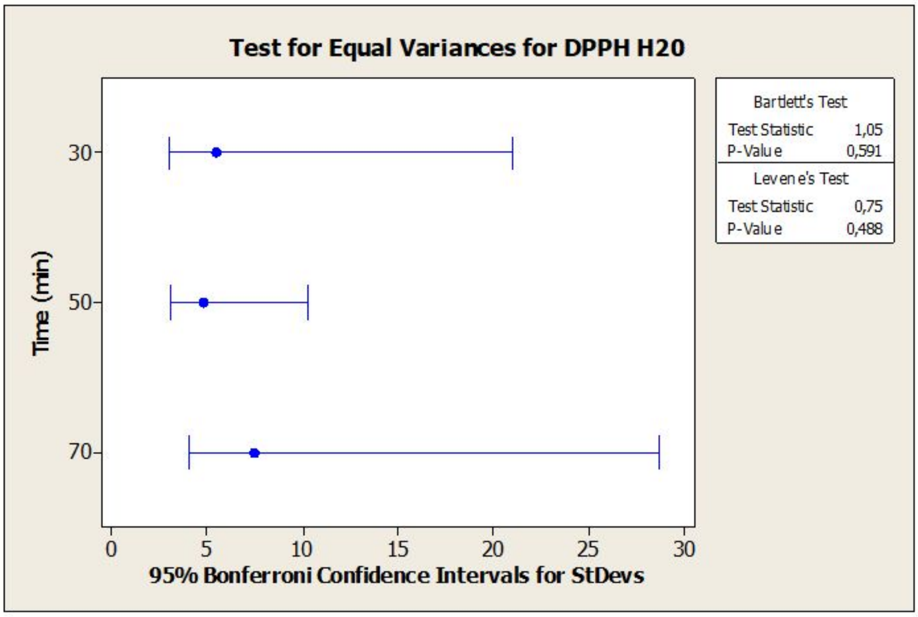

Figure 33S. Test for equal variances: DPPH H_2O versus time (min)

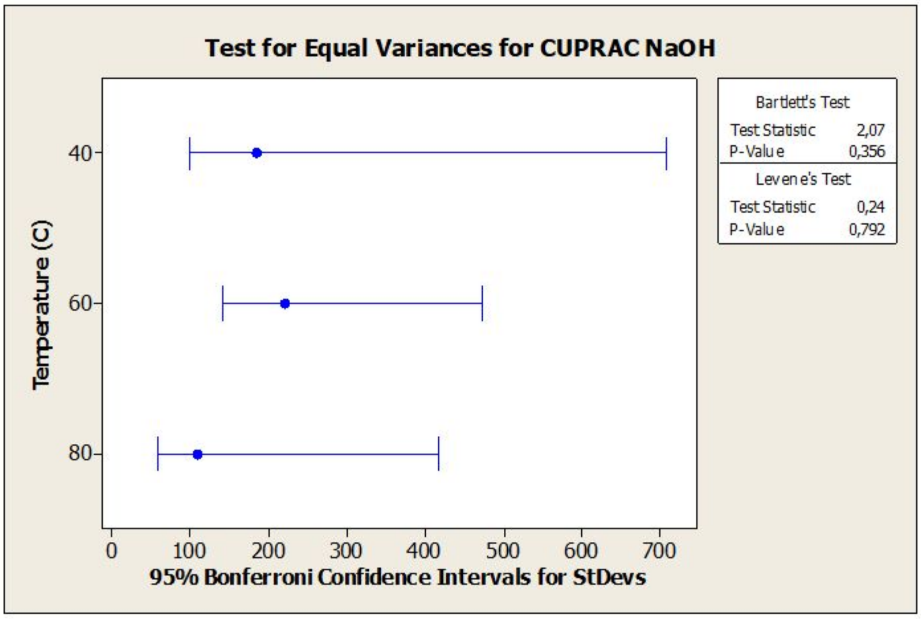

Figure 34S. Test for equal variances: CUPRAC NaOH versus temperature (C)

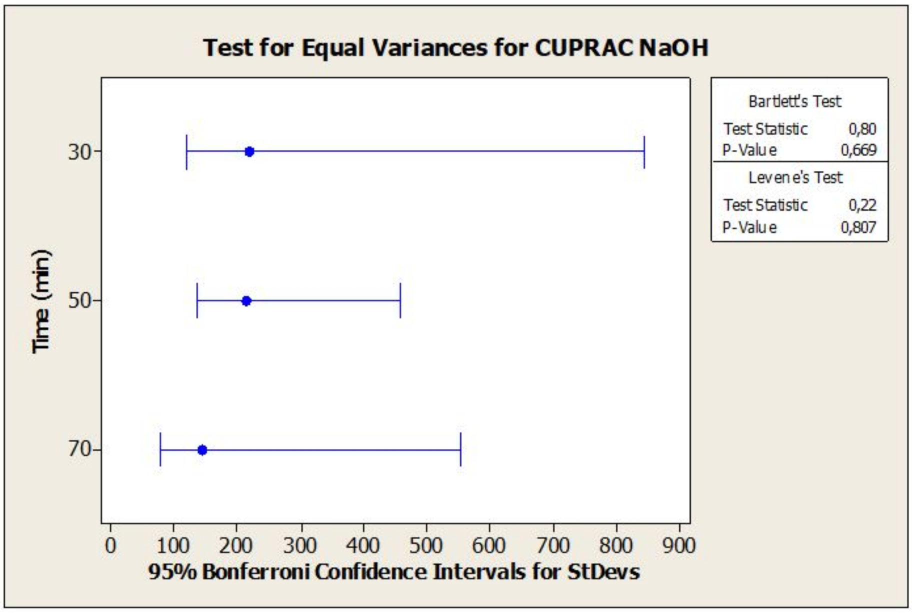

Figure 35S. Test for equal variances: CUPRAC NaOH versus alkali solution (%)

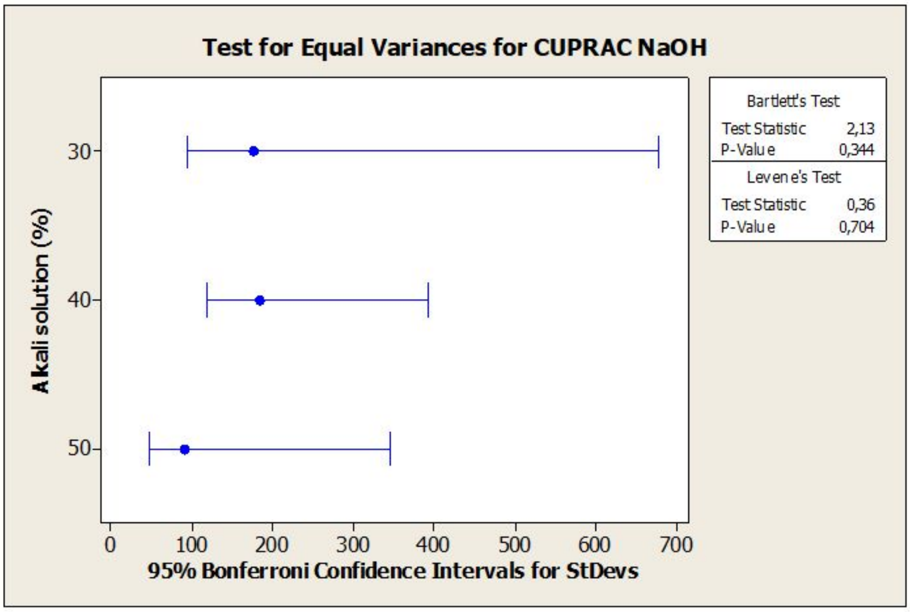

Figure 36S. Test for equal variances: CUPRAC NaOH versus time (min)

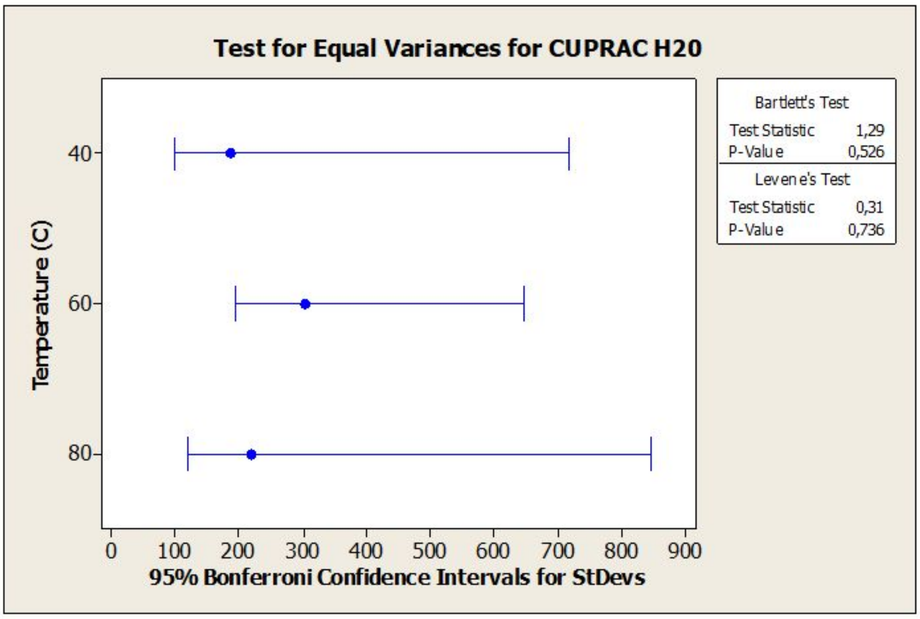

Figure 37S. Test for equal variances: CUPRAC H_2O versus temperature (C)

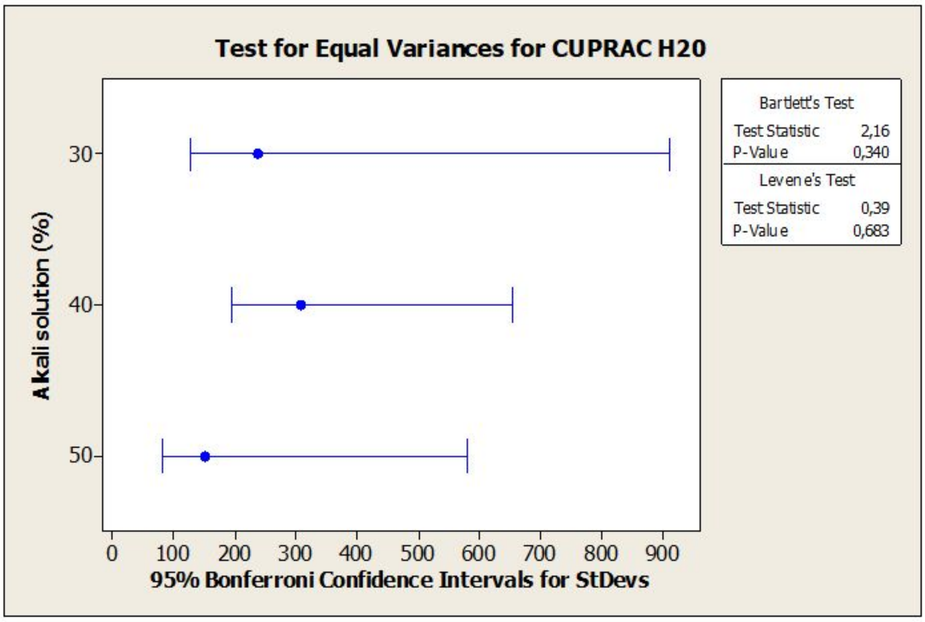

Figure 38S. Test for equal variances: CUPRAC H_2O versus alkali solution (%)

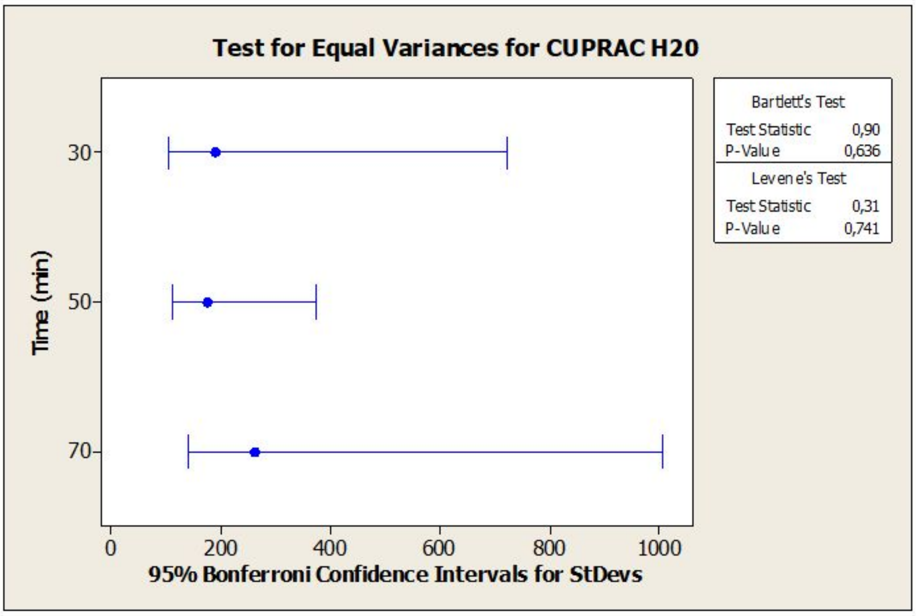

Figure 39S. Test for equal variances: CUPRAC H_2O versus time (min)

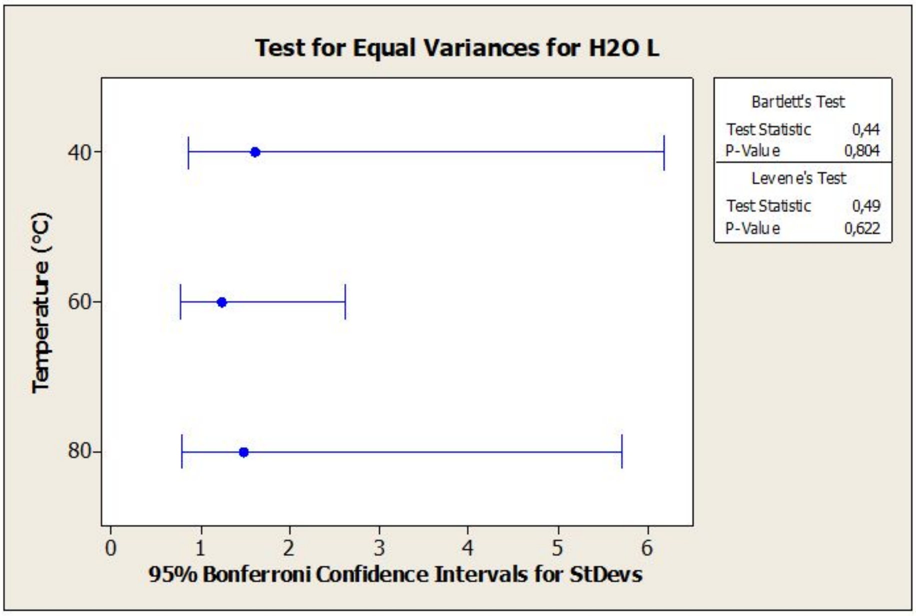

Figure 40S. Test for equal variances: H_2O L* versus temperature (°C)

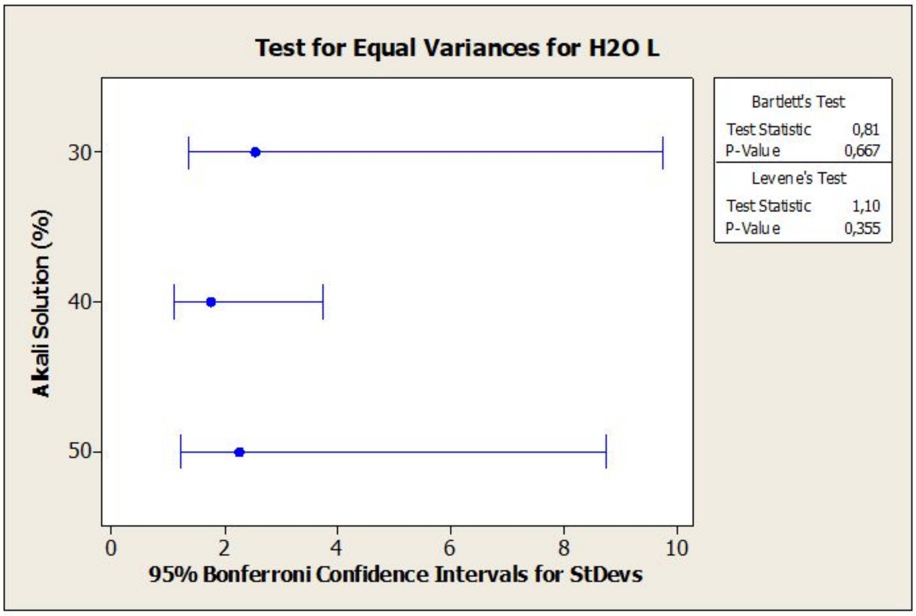

Figure 41S. Test for equal variances: H_2O L* versus alkali solution (%)

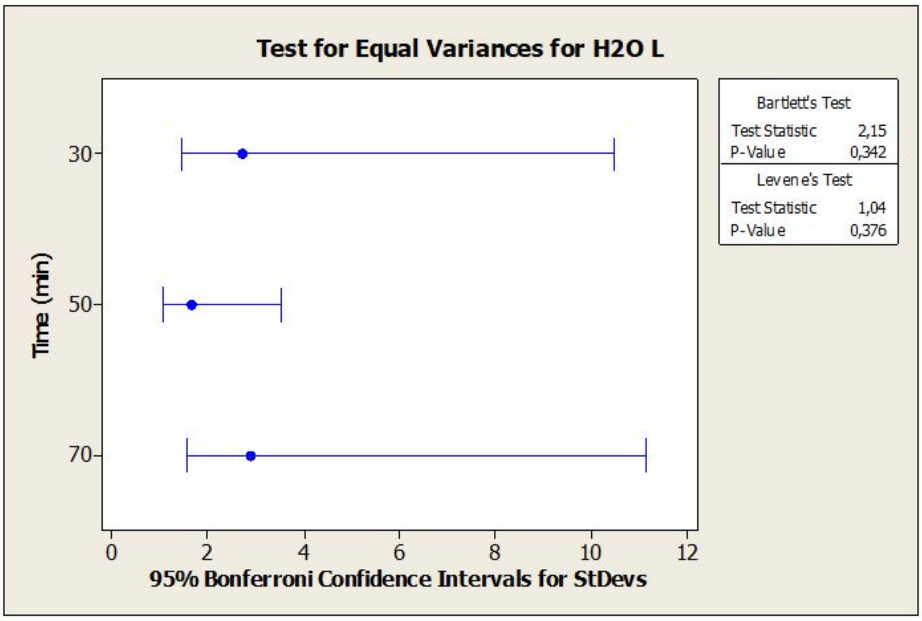

Figure 42S. Test for equal variances: H_2O L* versus time (min)

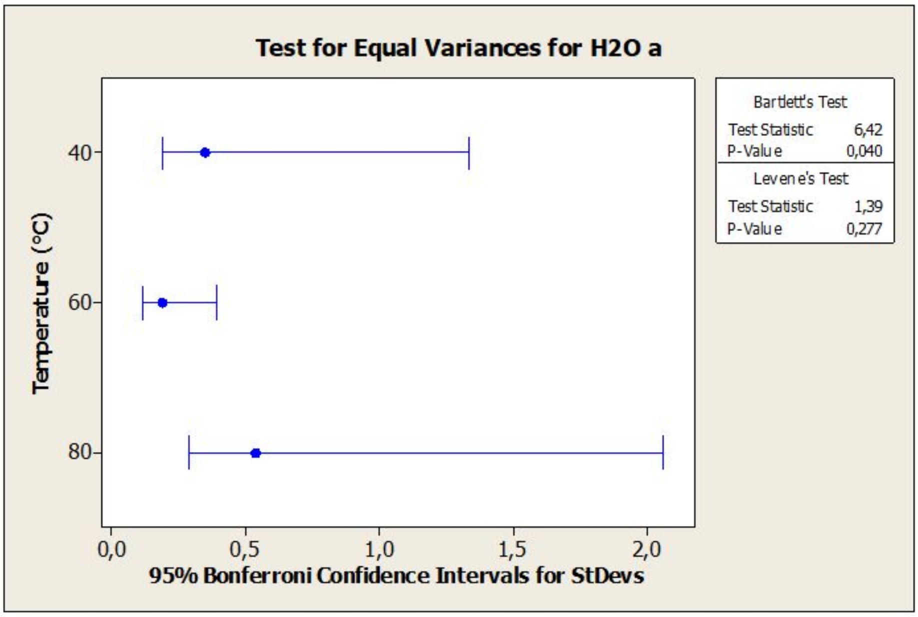

Figure 43S. Test for equal variances: H_2O a* versus temperature (°C)

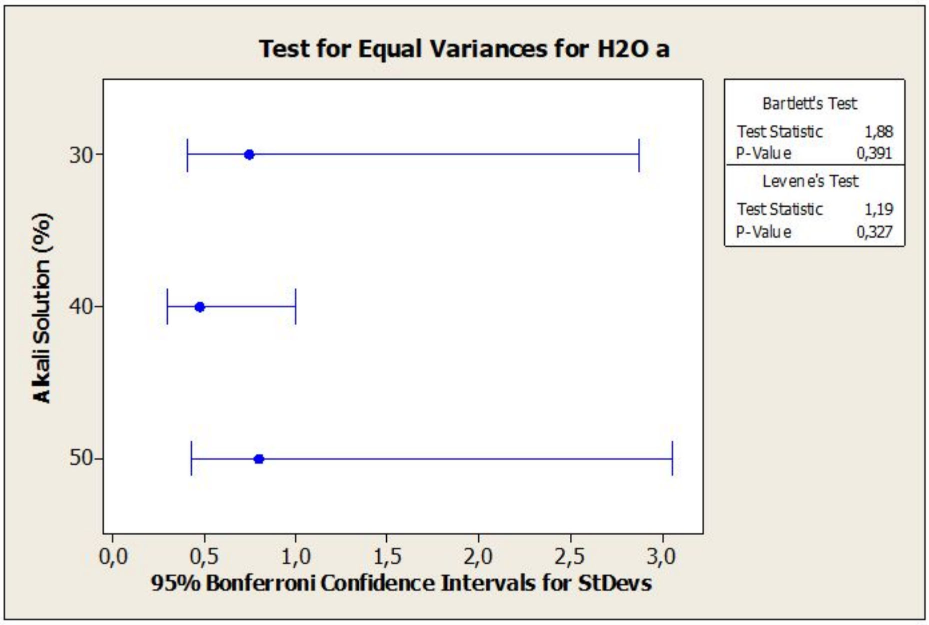

Figure 44S. Test for equal variances: H_2O a* versus alkali solution (%)

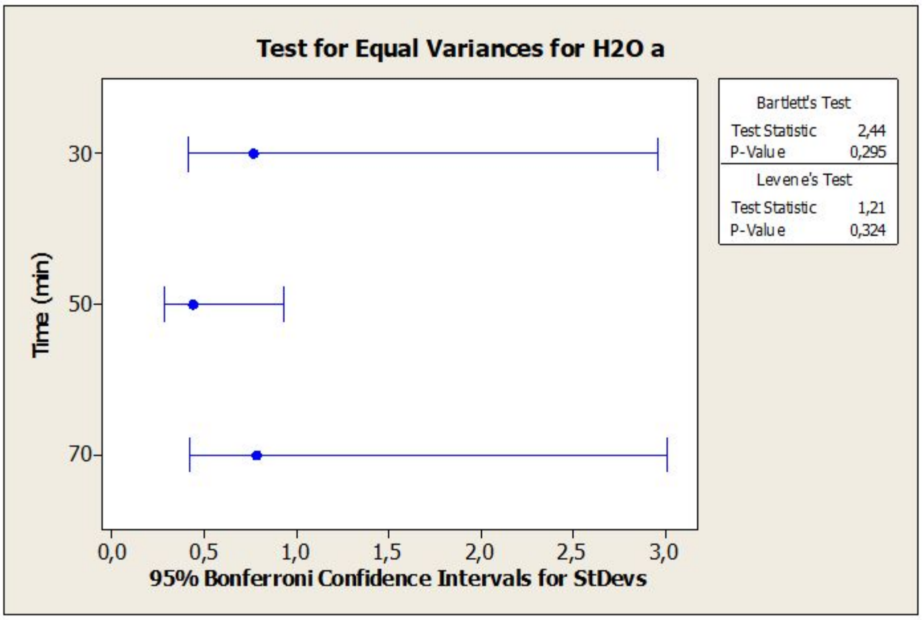

Figure 45S. Test for equal variances: H_2O a* versus time (min)

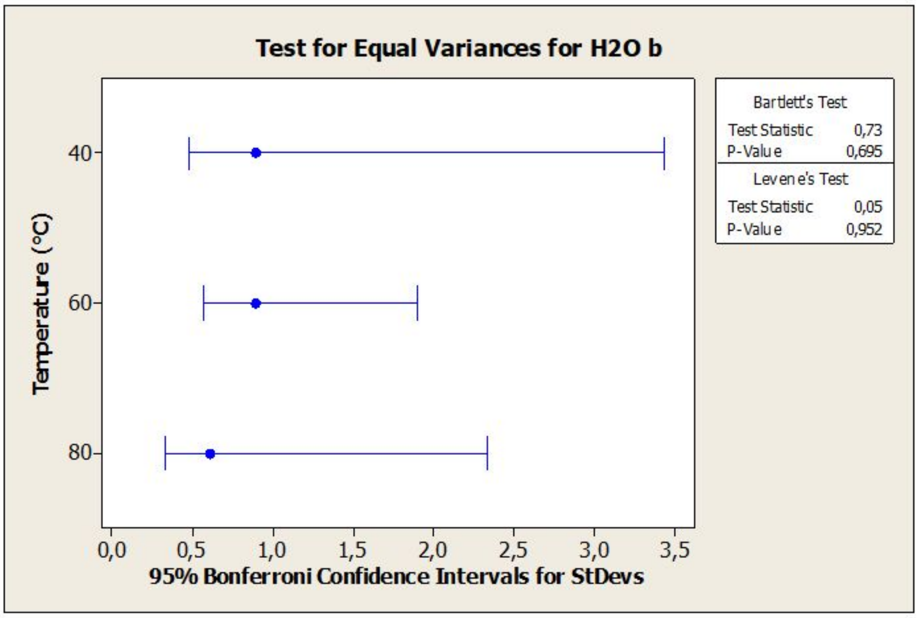

Figure 46S. Test for equal variances: H_2O b* versus temperature (°C)

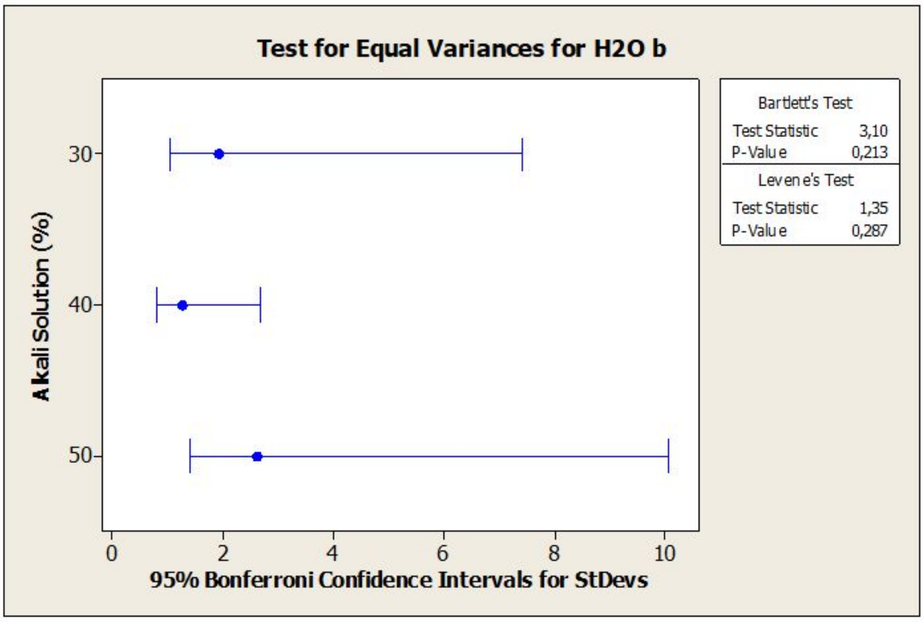

Figure 47S. Test for equal variances: H_2O b* versus alkali solution (%)

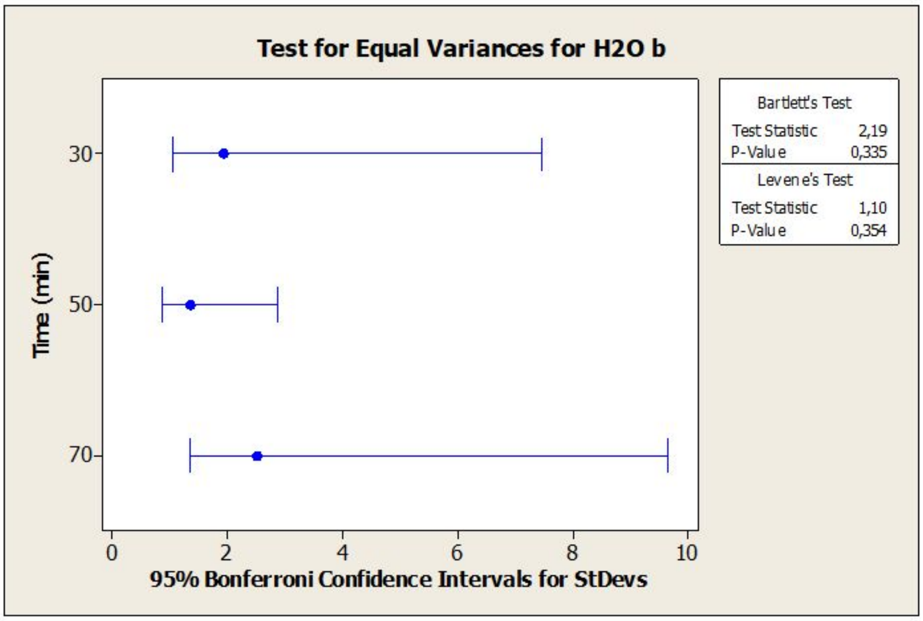

Figure 48S. Test for equal variances: H_2O b* versus time (min)

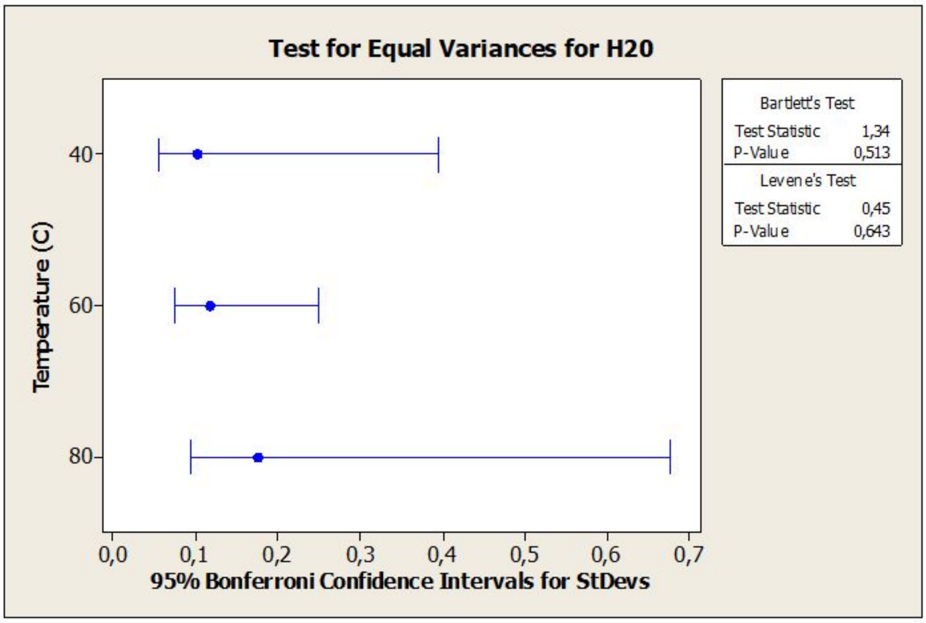

Figure 49S. Test for equal variances: H_2O TrMP/TMP versus temperature (C)

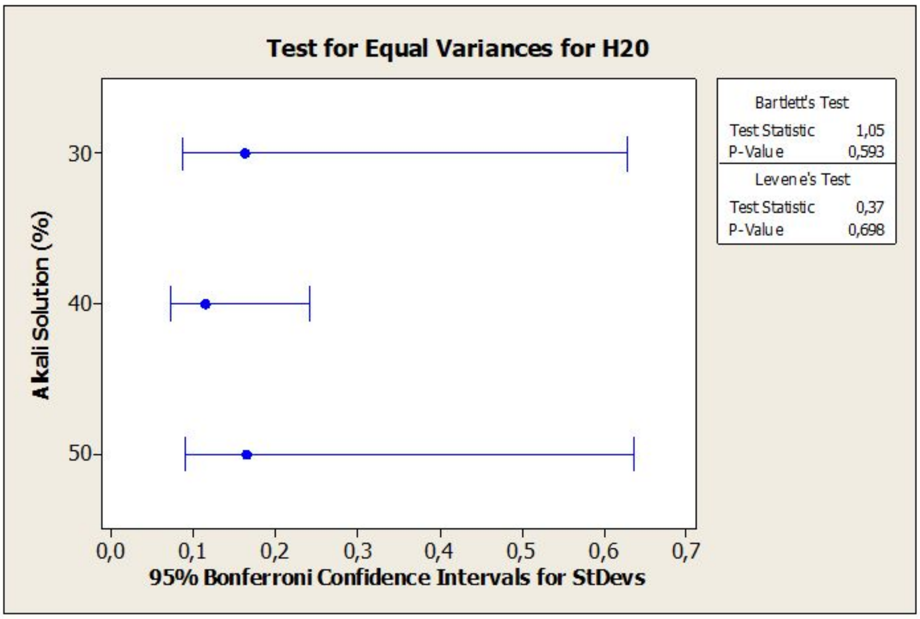

Figure 50S. Test for equal variances: H_2O TrMP/TMP versus alkali solution (%)

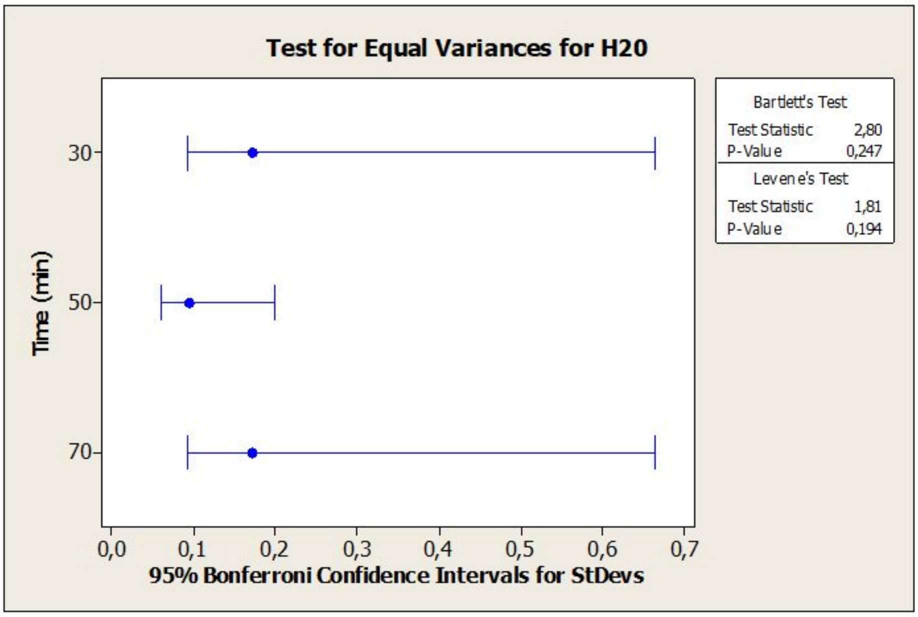

Figure 51S. Test for equal variances: H_2O TrMP/TMP versus time (min)

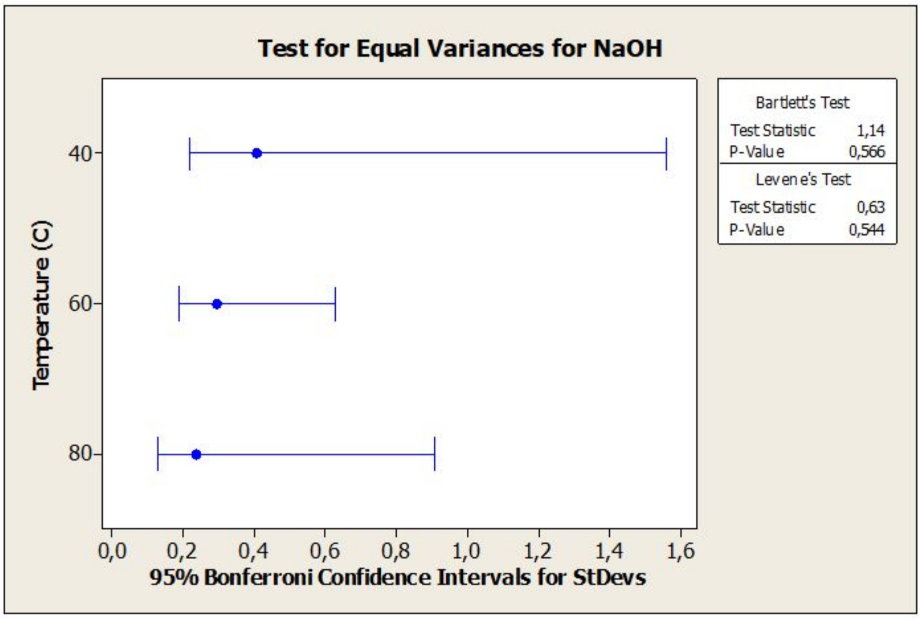

Figure 52S. Test for equal variances: NaOH TrMP/TMP versus temperature (C)

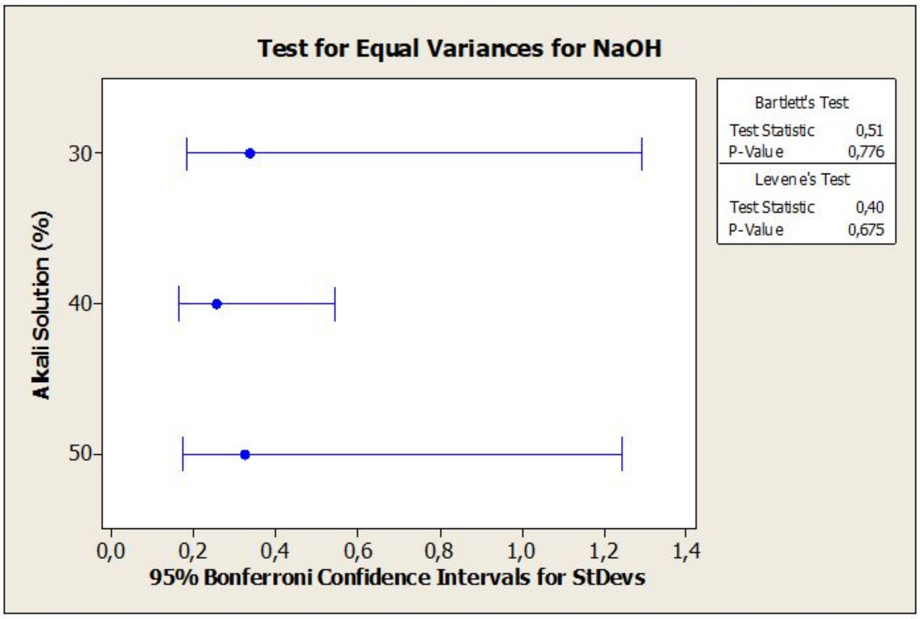

Figure 53S. Test for equal variances: NaOH TrMP/TMP versus alkali solution (%)

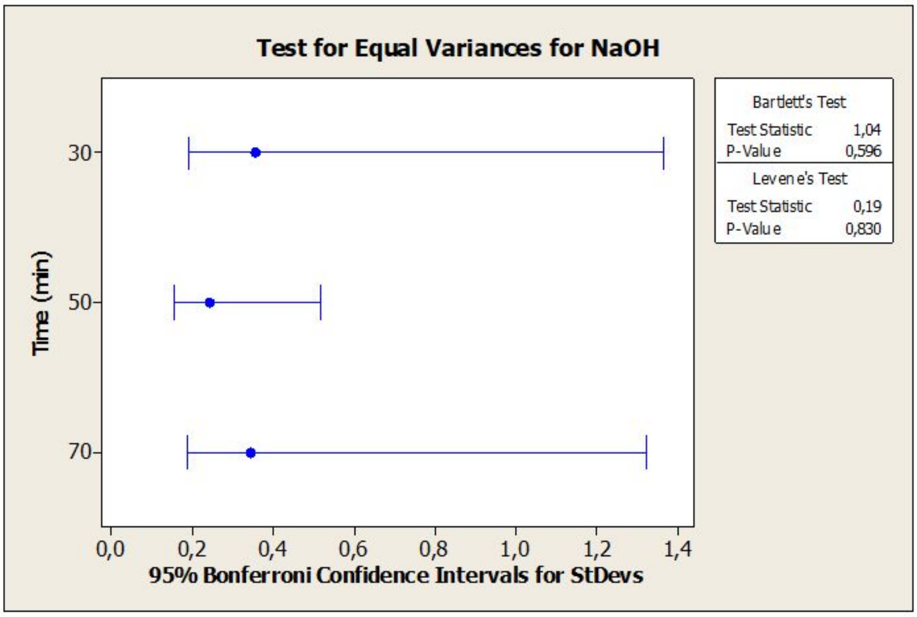

Figure 54S. Test for equal variances: NaOH TrMP/TMP versus time (min)

CORRELATION STUDY RESULTS

Table 29S. The correlations between the antioxidant capacity, TPC and phenolic profile of H_2O- and NaOH-treated cocoa powders.

	NaOH			H_2O		
	DPPH	CUPRAC	TPC	DPPH	CUPRAC	TPC
CUPRAC	0.275			-0.121		
	0.240			*0.612*		
TPC	0.542	0.401		-0.424	0.469	
	*0.014**	*0.080*		*0.063*	*0.037**	
PP	-0.100	0.749	0.241	-0.701	0.448	0.480
	0.675	*0.000**	*0.306*	*0.001**	*0.047**	*0.032**

Cell content; Pearson correlation coefficient and *p-value*. "*" a significant correlation coefficient (p<0.05).

Table 30S. The correlations between DPPH CUPRAC, TPC results and color (*L*, a*, b**) parameters.

	NaOH-treated samples			H_2O-treated samples		
	DPPH	CUPRAC	TPC	DPPH	CUPRAC	TPC
*L**	0.610	0.475	0.850	-0.538	0.209	0.439
	*0.004**	*0.034**	*0.000**	*0.014**	*0.376*	*0.053*
*a**	0.624	0.636	0.422	-0.566	0.060	0.260
	*0.003**	*0.003**	*0.064*	*0.009**	*0.803*	*0.269*
*b**	0.715	0.473	0.862	-0.557	-0.024	0.244
	*0.000**	*0.035**	*0.000**	*0.011**	*0.919*	*0.300*

Cell content; Pearson correlation coefficient and *p-value*. "*" a significant correlation coefficient (p<0.05).
